# Metabolic pathway-based subtypes associate glycan biosynthesis and treatment response in head and neck cancer

**DOI:** 10.1038/s41698-024-00602-0

**Published:** 2024-05-23

**Authors:** Benedek Dankó, Julia Hess, Kristian Unger, Daniel Samaga, Christoph Walz, Axel Walch, Na Sun, Philipp Baumeister, Peter Y. F. Zeng, Franziska Walter, Sebastian Marschner, Richard Späth, Olivier Gires, Timm Herkommer, Ramin Dazeh, Thaina Matos, Lisa Kreutzer, Johann Matschke, Katharina Eul, Frederick Klauschen, Ulrike Pflugradt, Martin Canis, Ute Ganswindt, Joe S. Mymryk, Barbara Wollenberg, Anthony C. Nichols, Claus Belka, Horst Zitzelsberger, Kirsten Lauber, Martin Selmansberger

**Affiliations:** 1 Research Unit Translational Metabolic Oncology, Institute for Diabetes and Cancer, Helmholtz Zentrum München Deutsches Forschungszentrum für Gesundheit und Umwelt (GmbH), Neuherberg, Germany; 2 Clinical Cooperation Group “Personalized Radiotherapy in Head and Neck Cancer, ” Helmholtz Zentrum München Deutsches Forschungszentrum für Gesundheit und Umwelt (GmbH), Neuherberg, Germany; 3 Department of Radiation Oncology, University Hospital, LMU Munich, Munich, Germany; 4 Bavarian Cancer Research Center (BZKF), Munich, Germany; 5 German Cancer Consortium (DKTK), Partner Site Munich, German Cancer Research Center (DKFZ), Heidelberg, Germany; 6 Institute of Pathology, Faculty of Medicine, LMU Munich, Munich, Germany; 7 Research Unit Analytical Pathology, Helmholtz Zentrum München Deutsches Forschungszentrum für Gesundheit und Umwelt (GmbH), Neuherberg, Germany; 8 Department of Otorhinolaryngology, Head and Neck Surgery, LMU University Hospital, LMU Munich, Munich, Germany; 9 Comprehensive Cancer Center, Munich, Germany; 10 Department of Pathology and Laboratory Medicine, University of Western Ontario, London, ON Canada; 11 Department of Otolaryngology - Head and Neck Surgery, University of Western Ontario, London, ON Canada; 12 Institute of Cell Biology (Cancer Research), University Hospital Essen, University of Duisburg-Essen, Essen, Germany; 13 German Cancer Consortium (DKTK) partner site Essen a partnership between DKFZ and University Hospital, Essen, Germany; 14 Department of Radiation Oncology, Innsbruck Medical University, Innsbruck, Austria; 15 Comprehensive Cancer Center Innsbruck, Innsbruck, Austria; 16 Department of Microbiology & Immunology, University of Western Ontario, London, ON Canada; 17 Department of Oncology, University of Western Ontario, London, ON Canada; 18 Clinic of Otorhinolaryngology, Klinikum rechts der Isar, Technical University Munich, Munich, Germany; 19 Department of Otorhinolaryngology, University of Luebeck, Luebeck, Germany

**Keywords:** Head and neck cancer, Head and neck cancer

## Abstract

Head and Neck Squamous Cell Carcinoma (HNSCC) is a heterogeneous malignancy that remains a significant challenge in clinical management due to frequent treatment failures and pronounced therapy resistance. While metabolic dysregulation appears to be a critical factor in this scenario, comprehensive analyses of the metabolic HNSCC landscape and its impact on clinical outcomes are lacking. This study utilized transcriptomic data from four independent clinical cohorts to investigate metabolic heterogeneity in HNSCC and define metabolic pathway-based subtypes (MPS). In HPV-negative HNSCCs, MPS1 and MPS2 were identified, while MPS3 was enriched in HPV-positive cases. MPS classification was associated with clinical outcome post adjuvant radio(chemo)therapy, with MPS1 consistently exhibiting the highest risk of therapeutic failure. MPS1 was uniquely characterized by upregulation of glycan (particularly chondroitin/dermatan sulfate) metabolism genes. Immunohistochemistry and pilot mass spectrometry imaging analyses confirmed this at metabolite level. The histological context and single-cell RNA sequencing data identified the malignant cells as key contributors. Globally, MPS1 was distinguished by a unique transcriptomic landscape associated with increased disease aggressiveness, featuring motifs related to epithelial-mesenchymal transition, immune signaling, cancer stemness, tumor microenvironment assembly, and oncogenic signaling. This translated into a distinct histological appearance marked by extensive extracellular matrix remodeling, abundant spindle-shaped cancer-associated fibroblasts, and intimately intertwined populations of malignant and stromal cells. Proof-of-concept data from orthotopic xenotransplants replicated the MPS phenotypes on the histological and transcriptome levels. In summary, this study introduces a metabolic pathway-based classification of HNSCC, pinpointing glycan metabolism-enriched MPS1 as the most challenging subgroup that necessitates alternative therapeutic strategies.

## Introduction

Head and neck squamous cell carcinomas (HNSCCs) derive from the mucosal epithelium and manifest in the oral cavity, pharynx, and larynx. The main risk factors are tobacco and alcohol consumption and human papillomavirus (HPV) infection^1^. Due to the divergent etiology, HNSCCs reveal a high degree of heterogeneity on all molecular levels^2,3^. Clinical management of HNCC is essentially dependent on the disease stage. Most cases require multimodality approaches, including surgery, radiation and/or chemotherapy. For recurrent or metastatic disease, targeted therapies and immunotherapies are available. Despite combined multimodality treatment approaches, the five-year survival rate for locally advanced cases remains mediocre, ranging from 50 to 60%^1^.

A major determinant of therapeutic failure is the intrinsic and/or acquired resistance of HNSCC cells to irradiation and/or chemotherapy. While HPV-associated tumors show a generally favorable clinical response compared to HPV-negative HNSCC, two intrinsically different subtypes of HPV-positive tumors were recently reported, including a subgroup of tumors with increased NFκB signaling and increased radiosensitivity^4^. Additionally, the composition of the tumor microenvironment (TME), including endothelial cells, cancer-associated fibroblasts (CAFs), and immune cells, as well as non-cellular characteristics, such as components of the extracellular matrix, oxygen and nutrient supply, pH-value, and others, strongly influences the treatment outcome^5^. In this scenario, hypoxia, immune infiltration, inflammatory signaling, and tumor-stroma interactions (e.g. with CAFs) confer metabolic reprogramming of the tumor with relevant implications for the response towards therapy^6,7^. Accordingly, understanding metabolic heterogeneity and characterizing metabolic phenotypes of HNSCCs is of pivotal importance to gain insights into the mechanisms of treatment resistance and to provide a basis for the development of alternative therapeutic approaches for high-risk cases.

Currently, only a constrained body of research investigating metabolic pathway dysregulations and metabolic heterogeneity for the purpose of diagnostic stratification is available from other cancer entities^8^. In HNSCC, transcriptome and genome analyses revealed molecular heterogeneity within tumors and between matched primary-recurrent tumor pairs. The transcriptome-based identification of molecular subtypes exhibited distinct biological characteristics^9,10^, and identified intratumoral heterogeneity as a relevant factor for clinical treatment planning and treatment success^11,12^. Nevertheless, comprehensive analyses of metabolic phenotypes based on transcriptional profiling in HNSCC are missing.

The present study was designed to examine metabolic heterogeneity and to identify metabolic subtypes derived from transcriptomic data of clinical HNSCC samples. However, HPV-positive tumors were included into the study to provide a comprehensive picture of the diverse entity of HNSCC. Starting with HPV-negative cases of four independent clinical cohorts, we were able to determine metabolic pathway-based subtypes (MPS) with distinct transcriptional profiles and association to clinical outcome. Adding HPV-positive cases in the next step provided an even more comprehensive picture of metabolic diversity in HNSCC. Beyond metabolism, the MPS classes revealed clear differences in their transcriptomic landscapes and their expression levels of cancer-relevant gene signatures. Immunohistochemical and histopathologic analyses as well as pilot MALDI mass spectrometric imaging (MALDI-MSI) experiments confirmed the transcriptional MPS classification on the metabolite level. Finally, proof-of-concept data from established HNSCC cell lines and orthotopic xenografts demonstrated the replicability of the metabolic HNSCC phenotypes both in vitro and in preclinical animal models, thus enabling future mechanistic perturbation studies and preclinical trials of novel therapeutic strategies.

Furthermore, in conjunction with this study, we have made novel transcriptomic data from our in-house HNSCC clinical collective publicly available, as well as an R library (*MetabolicExpressR*) for standardized and reproducible metabolic subtyping in cancer transcriptomic data sets.

## Results

To explore metabolic heterogeneity and the existence of distinct metabolic phenotypes in HNSCC, we performed transcriptome analyses in four different patient cohorts. As a first step, we investigated how metabolic dysregulation occurs in HNSCC on the transcriptome and proteome level. To this end, we compared metabolic gene and protein expression levels between normal and tumor tissues and analyzed the congruence of mRNA and protein data. The observed divergence in metabolic gene expression across different cases prompted us to hypothesize that distinct metabolic HNSCC phenotypes exist and that they can be assigned on the transcriptome level. For a clear definition of these metabolic pathway-based subtypes (MPS), we made use of the KEGG metabolic pathways collection, gene set variation analysis (GSVA), and k-means clustering. The metabolic phenotypes were compared to established transcriptomic HNSCC subtype classifications, their general transcriptomic landscape was explored, their association with clinical prognosis was examined, and validation on the metabolite level was achieved by immunohistochemical analysis of a key metabolite as well as pilot MALDI mass spectrometry imaging (MALDI-MSI) experiments. The metabolic phenotype with particularly impaired clinical outcome was further investigated by in-depth molecular characterization and computational deconvolution-based and histopathologic analysis of the tumor microenvironment (TME). Finally, we provide proof-of-concept evidence that MPS phenotypes as well as their molecular characteristics can be replicated in HNSCC cell lines in vitro and in orthotopic xenotransplants derived thereof, thus opening the perspective of future mechanistic analyses and preclinical trials.

### Metabolic dysregulation in HNSCC at the gene and protein expression level

In this study, we selected 95 Kyoto Encyclopedia of Genes and Genomes (KEGG) metabolic pathways consisting of 1659 unique genes to analyze the metabolic characteristics of HNSCC on the transcriptome level. Using publicly available data of the CPTAC-HNSCC cohort^13^, we observed a significant positive correlation between mRNA and protein expression for the majority of metabolic genes (94%, 1042 out of 1107 available metabolic genes/proteins, median Spearman correlation *r* = 0.592, Supplementary Fig. 1a). These findings demonstrate that mRNA expression of metabolic genes can reliably represent protein abundance and bears the potential to provide meaningful insights into metabolic processes based on transcriptomic data. Furthermore, differential gene and protein expression analysis between matched tumor and normal samples revealed dysregulation of manifold metabolic genes and proteins (*P*. adj<0.05) pointing towards tumor-specific metabolic processes (Supplementary Fig. 1d, e). Principal component analysis (PCA) of primary tumor and normal tissue samples incorporating metabolic gene/protein expression data revealed a) a clear separation of tumor and normal samples and b) a pronounced level of variance in metabolic gene expression across tumor samples suggesting the existence of different metabolic phenotypes in HNSCC (Supplementary Fig. 1b, c).

### Identification of metabolic pathway-based subtypes (MPS) in four independent HNSCC cohorts

**Fig. 1:**
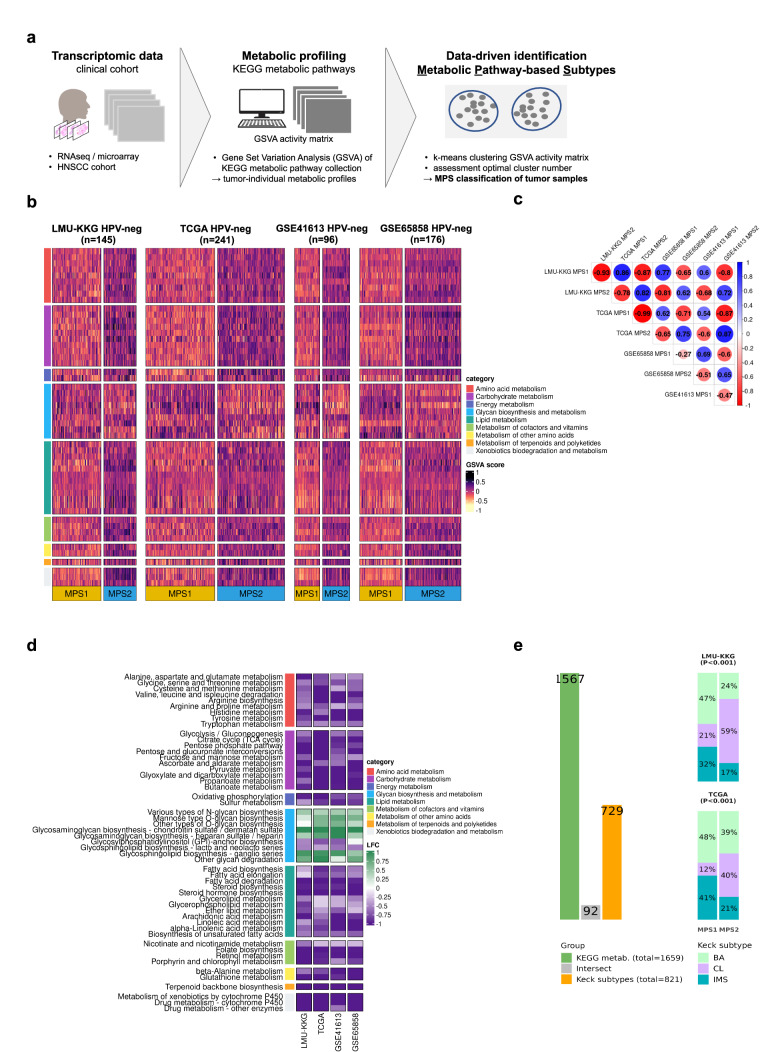
Metabolic pathway-based subtypes identified in four independent gene expression cohorts. Transcriptomic data from clinical cohorts were used for KEGG metabolic pathway enrichment quantification by GSVA. GSVA metabolic enrichment matrices were subjected to k-means clustering (with optimal *k* = 2) for unsupervised metabolic subtype identification (**a**). Heatmaps of KEGG metabolic pathways enrichment scores, according to the k-means clustering (k = 2) for the LMU-KKG (*n* = 145), TCGA-HNSC (*n* = 241), GSE41613 (*n* = 96), and GSE65858 (*n* = 176) cohorts, respectively (HPV-neg. only). MPS1 and MPS2 were independently delineated in the four cohorts. 52 metabolic pathways with significant (P adj.<0.05) differences in at least three cohorts between MPS1 and MPS2 are visualized (**b**). Correlation plot including Pearson’s coefficients of MPS-specific centroids between the four cohorts (**c**). MPS1 vs. MPS2 log2 fold changes (LFC) of GSVA enrichment scores for the 52 metabolic pathways in the four cohorts (**d**). Comparison of gene sets used for MPS and “Keck classification”, respectively. MPS1/2 with significantly different Keck subtype frequencies (consistent in LMU-KKG and TCGA) (**e**). MPS1 is enriched in IMS and BA cases, while CL cases are overrepresented in MPS2. Fisher’s exact test *P*-value on MPS and Keck subtype <0.001 for both cohorts. BA basal, CL classical, IMS inflamed/mesenchymal, NT matched normal, TP primary tumor, NS non-significant.

To infer metabolic heterogeneity in HNSCC, we determined enrichment scores for 95 KEGG metabolic pathway gene sets in the HPV-negative subsets of four independent clinical HNSCC cohorts (LMU-KKG (*n* = 145), TCGA (*n* = 241), GSE65858 (*n* = 176), and GSE41613 (*n* = 96)) using Gene Set Variation Analysis (GSVA). The resulting GSVA matrices were subjected to k-means clustering, revealing an optimum of *k* = 2 clusters and, accordingly, two distinct MPS subtypes in each of the four independent cohorts (Fig. 1a): MPS1, characterized by unique upregulation of glycosaminoglycan (GAG) metabolism pathways, and MPS2, associated with broad-spectrum upregulation of multiple pathways from different metabolic categories, including amino acid and lipid metabolism (Fig. 1b, d). These findings illustrate the metabolic diversity within the subgroup of HPV-negative HNSCCs and substantiate our initial hypothesis that distinct metabolic phenotypes exist across independent clinical cohorts. To validate the presence and consistency of the MPS classes and their specific metabolic gene expression profiles, we further applied two complementary approaches. First, correlation analyses of GSVA metabolic pathway score profiles for MPS1 and MPS2 revealed a high degree of congruence in MPS classification as obtained independently in the four data sets (Fig. 1c). Second, the methodologically alternative approach of nearest shrunken centroids (NSC) classification with classifiers trained on one cohort and applied to the other cohort, showed good agreement with k-means clustering-based classification for LMU-KKG and TCGA (classification consistency rates of 0.897 and 0.880, Supplementary Fig. 2a, b).

The observation that different methodological approaches applied to four independent clinical cohorts precipitated in robust and congruent classification of two MPS subtypes supported the validity of the newly defined metabolic classification and stimulated us to compare them to existing transcriptomic HNSCC subtype classifications.

To describe the association of the MPS-classification with the existing transcriptome-based “Keck subtypes”^10^ which represent a widely used molecular stratifier in HNSCC, we first compared the KEGG metabolic gene compilation (*n* = 1659) used for MPS classification with the genes employed for molecular subtyping of HNSCC by Keck et al. (*n* = 821) and found a marginal overlap of 92 genes only. Nevertheless, the two MPS groups exhibited significantly different distributions of “Keck subtypes” (Fisher’s exact test *P* < 0.001, in both the LMU-KKG and TCGA cohorts). MPS1 was enriched in cases of the basal (BA) and inflamed/mesenchymal (IMS) subtypes, while the classical (CL) subtype was overrepresented in MPS2 (Fig. 1e).

### Metabolic gene expression of MPS1 and MPS2 cases in comparison to normal tissue samples on the RNA and protein level and pilot metabolite level analyses

We further investigated the MPS on protein level and compared MPS-classified tumors and normal tissue samples. For this purpose, we used the TCGA (*n* = 275 samples) and the CPTAC (*n* = 241 samples) cohorts, including adjacent normal tissue samples (TCGA: *n* = 34 matched normal samples; CPTAC: *n* = 53 matched normal samples with mRNA expression data and *n* = 62 with protein expression data), thus enabling a comparison with respective MPS-classified tumors. PCA based on metabolic gene or protein expression showed a separation of MPS classes within tumor tissue samples and a distinct normal tissue cluster for both cohorts (Supplementary Fig. 3a, c). Interestingly, tumor samples exhibited higher numbers of metabolic genes and proteins down- than upregulated (Supplementary Fig. 3b, d) and therefore lower overall metabolic pathway scores (gene and protein expression level) as compared to normal tissue samples. This reduced metabolic pathway expression – except for GAG-related pathways – might be attributed to the attenuation of energy consuming cellular functions and corresponding metabolic processes in tumors^14^. Both HNSCC cohorts indicated an MPS1-specific upregulation of GAG-related metabolic pathways compared to MPS2 and/or to the corresponding normal tissues (Supplementary Fig. 3e). Importantly, the consensus results on RNA level could be confirmed on the protein level in the CPTAC cohort – again demonstrating the validity of the transcriptome-derived MPS profiles (Supplementary Fig. 3e). Metabolite level data (metabolite abundance or flux measurements) are necessary to explore the association between metabolic gene/protein expression and metabolic activity. Unfortunately, systematic data are not available for the clinical cohorts analyzed within this study. In pilot experiments, we subjected exemplary tumor cases to MALDI-MSI analysis and identified m/z species previously described for fragments of chondroitin sulfate originating from glycan metabolism^15^, strongly enriched in MPS1 (Supplementary Fig. MALDI-MSI HE). This finding reinforces the RNA and protein level-based observation that GAG metabolism is upregulated in MPS1. Conversely, multiple m/z species associated with various metabolic processes from the energy, amino acid, and lipid metabolism categories were detected in greater abundance in MPS2 (Supplementary Fig. MALDI-MSI HE), once again confirming the RNA and protein level data.

### Association of MPS subtyping with clinical response in HPV-negative HNSCC

**Fig. 2:**
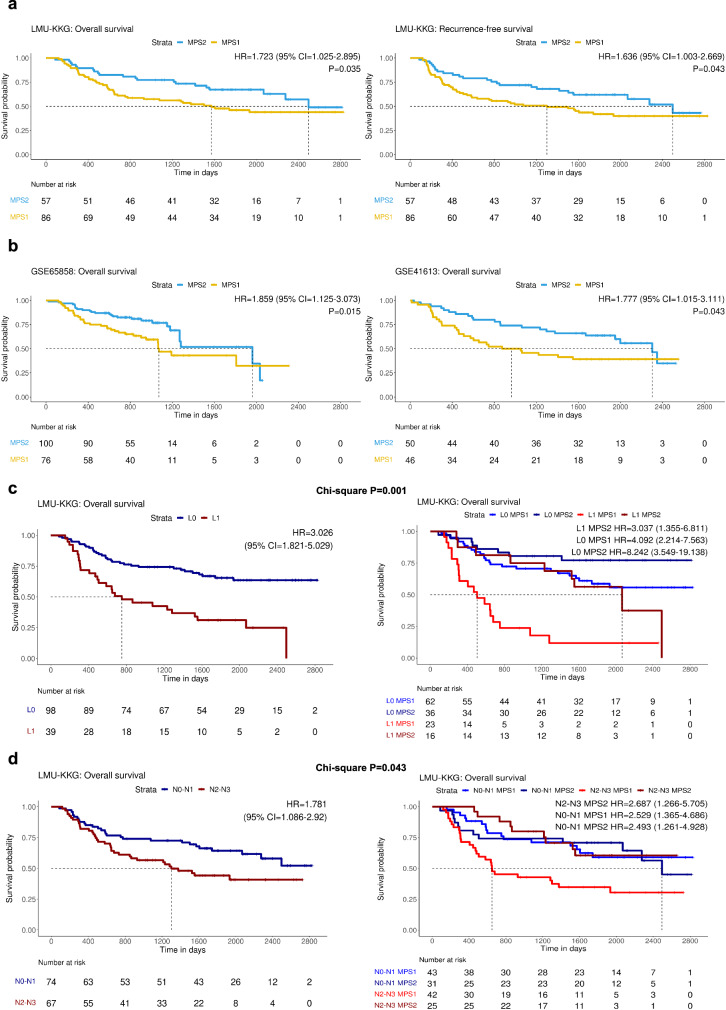
MPS is associated with radio(chemo)therapy response. Kaplan-Meier (KM) plots of the LMU-KKG cohort (HPV-negative) for overall survival (OS), and recurrence-free survival (RFS), respectively (**a**). MPS1 with significantly adverse OS, RFS, and locoregional recurrence-free survival (LR-RFS) compared to MPS2 (LR-RFS, and additional endpoints in Supplementary Figure 4a). Independent validation of MPS1 with adverse OS in two HPV-negative HNSCC data sets (GSE65858 and GSE41613) (**b**). Risk-group stratification and comparative KM analysis based on established clinical prognostic factors and MPS: lymphovascular invasion (LVI/L stage, **c**) and N stage (**d**) for endpoint OS. Clinical variables only (left) and in combination with MPS (right). The two models were compared by chi-square testing, and *P*-values are shown. Reference groups in pairwise comparisons are L1 MPS1 (**c**), and N2-3 MPS1 (**d**), HRs with 95% CI are indicated (additional clinical factors/endpoints in Supplementary Figure 6).

Next, we set out to assess the implications of divergent metabolic gene expression for clinical prognosis. No association between MPS1/MPS2 and previously reported prognostic clinical variables was observed by Fisher’s exact test in the LMU-KKG, TCGA, GSE65858, and GSE41613 HPV-negative cohorts (Supplementary Table 1), but univariable Cox Proportional-Hazards (Cox PH) modeling and Kaplan-Meier (KM) analysis revealed significantly impaired overall survival (OS) for MPS1 compared to MPS2 cases in the LMU-KKG, GSE6558, and GSE41613 cohorts (LMU-KKG, GSE65858, and GSE41613; *P* = 0.035, HR = 1.723, 95% CI = 1.025–2.895, *P* = 0.015, HR = 1.86, 95% CI = 1.125–3.073 and *P* = 0.043, HR = 1.777, 95% CI = 1.015–3.111, respectively) (Fig. 2a, b). Additionally, recurrence-free survival (RFS) and locoregional RFS (LR-RFS) were significantly reduced for MPS1 in the LMU-KKG cohort (logrank *P* = 0.043, HR = 1.636, 95% CI = 1.003-2.669 and logrank *P* = 0.044, HR = 1.65, 95% CI = 1.001–2.719, respectively) (Fig. 2a, Supplementary Fig. 4a). In contrast to MPS phenotypes, “Keck subtypes” exhibited no association with clinical endpoints (OS, RFS) in LMU-KKG (Supplementary Fig. 5), thereby validating the lack of a prognostic value of “Keck subtypes” in HPV-negative HNSCC as reported by Keck et al.^10^. Thus, MPS classification turned out to be independently associated with the clinical response of HPV-negative HNSCC upon adjuvant radio(chemo)therapy. In the subset of HPV-negative HNSCCs with adjuvant radio(chemo)therapy of the TCGA cohort, MPS classification did not reach statistical significance in association with OS (*n* = 77, logrank *P* = 0.45). This discrepancy may be explained by the multi-center nature of the TCGA cohort which increases heterogeneity in the applied therapeutic and follow-up schemes. More specific clinical endpoints beyond OS, such as RFS or LR-RFS, were not available for the TCGA cohort. In multivariable modeling, including previously reported clinical prognostic factors in HNSCC, addition of MPS classification into the modeling approach improved the model for RFS in the LMU-KKG data set as indicated by concordance-index (C-index) and HR. Multivariable modeling approaches for OS and LR-RFS showed nearly similar performance with and without inclusion of MPS classification (Table 1a).

**Table 1:** Risk modeling of clinical response using MPS class and CS/DS metabolism pathway enrichment scores

a						
Endpoint	Model with MPS			Model without MPS		
	Final variables	Risk score *P*-value, HR (95% CI)	C-index (95%, 85% CI)	Final variables	Risk score *P*-value, HR (95% CI)	C-index (95%, 85% CI)
OS	MPS, ECE, Resection margin status, LVI	0.001, 2.565 (1.443–4.560)	0.741 (0.603-0.878, 0.640-0.842)	ECE, Resection margin status, LVI	0.032, 1.698 (1.048–2.751)	0.761 (0.632–0.890, 0.666-0.855)
RFS	MPS, ECE, LVI	<0.001, 2.542 (1.530–4.222)	0.815 (0.714-0.916, 0.741-0.889)	ECE, LVI	0.024, 2.182 (1.630–2.489)	0.738 (0.598–0.879, 0.635-0.842)
LR-RFS	MPS, ECE, LVI	0.227, 1.382 (0.818–2.337)	0.597 (0.403-0.790, 0.454-0.739)	ECE, LVI	0.744, 1.086 (0.661–1.786)	0.585 (0.377–0.792, 0.432–0.737)

b			
Endpoint	*P*-value	Adj. *P*-value	HR (95% CI)
OS	0.014	0.016	2.867 (1.240–6.625)
RFS	0.006	0.014	3.121 (1.395–6.981)
LR-RFS	0.009	0.014	3.023 (1.326–6.894)
DSS	0.005	0.014	7.136 (1.825–27.91)
FFR	0.009	0.014	4.646 (1.459–14.79)
LRC	0.159	0.159	3.180 (0.636–15.910)
OS (GSE65858)	0.004	–	3.427 (1.476–7.957)
OS (GSE41613)	0.005	–	2.782 (1.367–5.665)

Multivariable Cox Proportional-Hazards (Cox PH) analysis results assessment with MPS and modeling without the inclusion of MPS on the LMU-KKG HPV-negative cohort (*n* = 145) (**a**) Prognostic association of CS/DS metabolism enrichment scores in univariable Cox PH analysis for the LMU-KKG HPV-negative (*n* = 145), and GSE65858 (*n* = 176) and GSE41613 (*n* = 96) cohorts as validation (**b**) *OS* overall survival, *RFS* recurrence-free survival, *LR-RFS* locoregional recurrence-free survival, *DSS* disease-specific survival, *FFR* freedom from recurrence, *LRC* locoregional control, *HR* hazard ratio, *C-index* concordance-index, *CSPG* chondroitin sulfate proteoglycan, *IHC* immunohistochemistry.

A remarkable improvement in clinical risk group stratification was demonstrated when MPS classification was combined with lymphovascular invasion (LVI), TNM N-stage, extracapsular extension (ECE), and perineural invasion (PNI), respectively, resulting in sub-stratification of four risk groups. All four-group Cox PH models exhibited significantly improved performance compared to the two-group Cox PH models based on single risk factors for OS and RFS (as determined by chi-square testing; Fig. 2c, d, Supplementary Figs. 6-7). The MPS sub-stratification of LVI, ECE, and N-stage identified high-risk groups with significantly elevated HRs when compared to the corresponding low-risk groups.

### Molecular characterization of the MPS1 phenotype

**Fig. 3:**
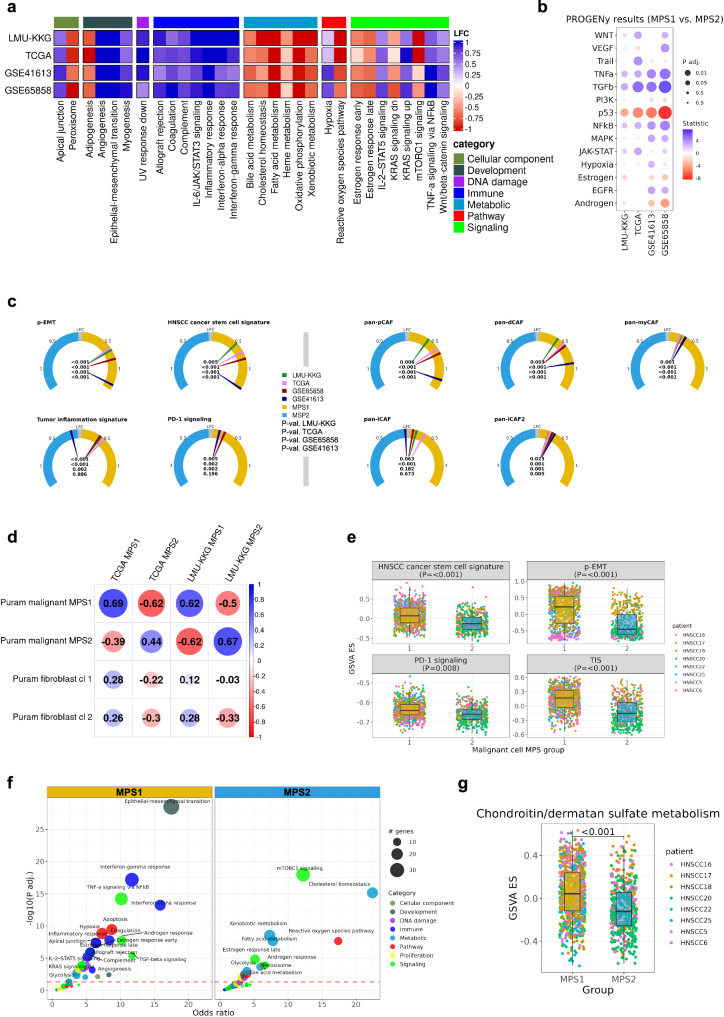
Functional characterization of the MPS in bulk and single-cell data. Differential MPS1 vs. MPS2 hallmarks analysis (GSVA scores) in four individual data sets (LMU-KKG, TCGA, GSE41613, and GSE65858, HPV-negative only) (**a**). Hallmarks with P adj.<0.05 in at least three cohorts are visualized. PROGENy differential analysis was performed and showed consistent results for eleven cancer-related signaling pathways in the four data sets (**b**). Gauge charts of LFC values of MPS1 vs. MPS2 with selected gene signatures (GSVA scores, Wilcoxon test, four data sets individually) (**c**). Correlation plot with Spearman correlation coefficients of the data set-specific MPS/cluster groups (based on KEGG metabolic GSVA enrichment scores) between Puram et al. malignant cells or fibroblast cells, and LMU-KKG or TCGA MPS classes (**d**). Gene signature comparisons between MPS1 and MPS2 malignant cells of the Puram et al. scRNAseq data (generalized linear mixed models) (**e**). Enrichment analysis results of MPS1 and MPS2 malignant cell clusters of the Puram et al. data set using the hallmarks signatures (dashed red line *P* adj.=0.05) (**f**). Significant upregulation of CS/DS metabolism enrichment scores in MPS1 compared to MPS2 malignant cells (generalized linear mixed models) (**g**). Cl cluster.

For molecular characterization of MPS1 we employed the consensus results derived from all four clinical cohorts and from available single-cell RNAseq (scRNAseq) data. In all data sets, MPS1 revealed a clear enrichment of several pathways from the glycan biosynthesis and metabolism category (*P* adj.<0.001 for all four data sets), including *glycosaminoglycan biosynthesis – chondroitin/dermatan sulfate* (CS/DS), *glycosphingolipid biosynthesis – ganglio series*, *glycosaminoglycan biosynthesis – heparan sulfate/heparin* (*P* adj. = 0.015, <0.001, 0.001, <0.001 for LMU-KKG, TCGA, GSE6558, and GSE41613), and *other glycan degradation* (all results in Supplementary Tables 2–5). These results were independently validated on the protein level of the CPTAC cohort (Supplementary Fig. 3e). Accordingly, multiple genes involved in the CS/DS pathway exhibited differential expression between MPS1 and MPS2 (Supplementary Fig. 8b, Supplementary Tables 6–8). The previously reported role of GAGs in cell-cell (including tumor cell-CAF) and cell-extracellular matrix (ECM) interactions^16,17^ prompted us to examine CAF subtype signatures, disclosing a clear enrichment of several CAF subtypes in the high-risk MPS1 tumors (consensus in all four data sets, Fig. 3c). This finding was well in line with CIBERSORTx analysis results pointing to elevated abundance of fibroblasts in the MPS1 subtype (Supplementary Fig. 9).

Taking advantage of scRNAseq data from Puram et al.^18^, we observed that the reported metabolic profiles of MPS1 and MPS2 predominantly originated from malignant cells and not from fibroblasts (CAFs) which represent the second most prevalent cell type in the bulk RNAseq data used for MPS classification (Fig. 3d, Supplementary Fig. 2c-f).

To achieve a comprehensive overview of transcriptomic landscape differences between MPS1 and MPS2, we independently applied gene set enrichment/variation analysis (GSEA/GSVA) using the Molecular Signatures Database (MSigDB) hallmarks gene set collection in the four cohorts and the scRNAseq data set. As expected, multiple metabolism-related hallmark gene sets were among the most prominently enriched in MPS2 (Fig. 3a, f). Furthermore, hallmark gene sets associated with immune mechanisms, including *inflammatory response*, *interferon-alpha response*, *interferon-gamma response*, and *coagulation* (Fig. 3a, f) were consistently enriched in MPS1, both on the bulk and (malignant) single-cell levels (Fig. 3a, f) (all results in Supplementary Tables 9-13). Similarly, the immune-related *tumor inflammation signature* (TIS) was upregulated in MPS1, paralleled by enhanced expression of the *PD-1 signaling* signature (Fig. 3c). These findings suggest an increased infiltration and/or activation of immune cells and inflammatory signaling pathways in the MPS1 subtype, potentially counteracted by immunosuppressive mechanisms. MPS1 (compared to MPS2) also showed enrichment of the developmental hallmark gene sets *angiogenesis* and *myogenesis* in the bulk (P adj.<0.001 all data sets) but not in the scRNAseq data of malignant cells only (Fig. 3a, f), implying that both might preferentially originate from fibroblasts and other stromal cell types with higher abundance in the MPS1 subtype (Supplementary Fig. 9, Fig. 3c). *Epithelial-mesenchymal transition* (EMT) was another developmental hallmark gene set with strong enrichment in MPS1 and in agreement with upregulation of the related partial EMT (*p-EMT*) signature (Fig. 3c, e). Both are known to contribute to cancer progression and treatment resistance and have been linked to cancer stemness and angiogenesis via the secretion of vascular endothelial growth factor (*VEGF*) or direct differentiation of cancer stem cells into endothelial-like cells, respectively^18^. This encouraged us to investigate the *HNSCC cancer stem cell* signature which similarly showed significant upregulation in MPS1 (bulk and scRNAseq data) (Fig. 3c, e). Interested in the potential upstream regulators responsible for the observed MPS1-specific gene expression patterns, we performed PROGENy analysis and identified *TNF* and *TGF-β signaling* to be positively associated with MPS1 gene expression, whereas *p53 signaling* showed negative association (Fig. 3b, Supplementary Table 14). *TNF* is recognized for its ability to mediate cytotoxicity of radio- and/or chemotherapy in HNSCC^19–21^. *TGF-β* is involved in several processes, such as tumor progression promotion, EMT, and the formation of an immunosuppressive TME. It is feasible to assume that both cytokine signaling pathways contribute to the regulation of the observed tumor-promoting processes and treatment failure in high-risk MPS1 tumors.

**Fig. 4:**
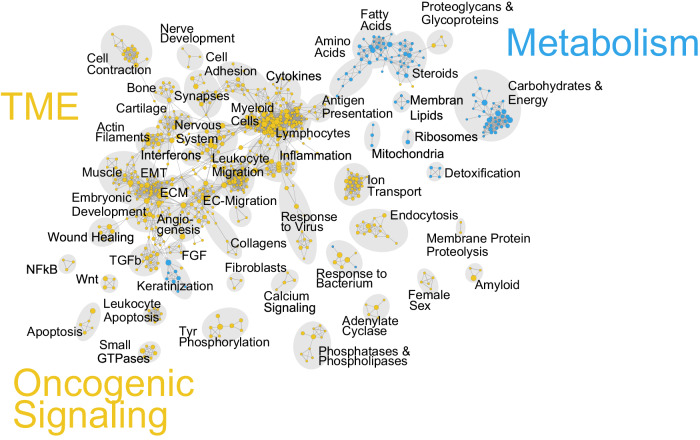
Integrated network visualization of enriched biological processes in MPS1 and MPS2 HPV-negative tumors. Integrated network visualization of MPS1 and MPS2 enriched biological processes was carried out using enrichmentMap. MPS1 vs. MPS2 differential gene expression results of the four cohorts were used to derive a mean-ranked list of genes and pre-ranked gene set enrichment analysis (GSEA) with the gene set collection Gene Ontology Biological Process (GO-BP) was performed and visualized using enrichmentMap. Yellow indicates MPS1-specific, and blue color indicates MPS2-specific terms.

Finally, a pre-ranked GSEA with the more detailed (compared to the hallmarks collection) Gene Ontology Biological Process (GO-BP) gene set compilation was performed using a mean-ranked gene list derived from the differential gene expression results of MPS1 vs. MPS2 in the four individual cohorts. Subsequent EnrichmentMap^22^ network visualization essentially confirmed and complemented the molecular characterization of the MPS phenotypes. Motifs of biological processes enriched in MPS1 are predominantly associated with TME organization and oncogenic signaling processes, while metabolic processes predominantly shape the biological process landscape of MPS2 (Fig. 4).

### Inclusion of HPV-positive HNSCC in MPS classification

**Fig. 5:**
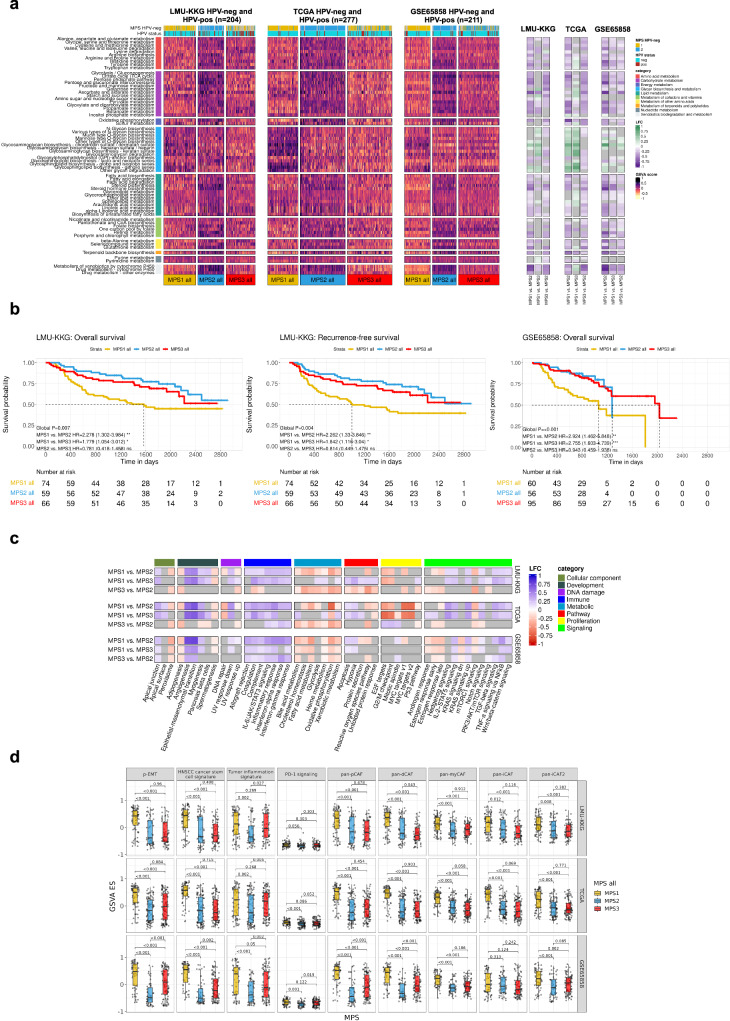
Inclusion of HPV-positive HNSCC in MPS classification. K-means clustering was performed with k = 3 on the HPV-negative and HPV-positive cases of the LMU-KKG, TCGA, and GSE65858 cohorts. Heatmaps of KEGG metabolic pathways GSVA enrichment scores, according to the classes obtained by k-means clustering (k = 3) for the LMU-KKG (*n* = 204), TCGA-HNSC (*n* = 277), and GSE65858 (*n* = 211) cohorts, respectively. 68 intersecting metabolic pathways are visualized. On the right-hand side, LFC values of GSVA scores per pathway for MPS1 vs. MPS2, gray color indicates P adj.>0.05 (**a**). KM plots of MPS1, MPS2, and MPS3 of the LMU-KKG and GSE65858 cohorts for OS and RFS (LMU-KKG only). Pairwise HRs with 95% CI. ***P-value < 0.001, **P-value >= 0.001 and < 0.01, *P-value >= 0.01 and < 0.05, ns: *P*-value >= 0.05. Global P: global logrank *P*-value (**b**). LFC values of hallmarks GSVA scores comparing the three MPS groups of the three data sets (LMU-KKG, TCGA, and GSE65858), gray color indicates *P* adj.>0.05 (**c**). Boxplots and differential testing of gene signatures (GSVA scores) compared between the three MPS of the three data sets, adjusted *P*-values are indicated (**d**).

In consideration of the fundamentally different etiology, pathogenesis, and prognosis of HPV-positive HNSCCs, they were deliberately excluded from our initial investigation of metabolic heterogeneity and the determination of metabolic subtypes. Now, to test if/how they integrate into the proposed MPS classification system, HPV-positive cases of the LMU-KKG (*n* = 204, comprising 145 HPV-negative and 59 HPV-positive cases), TCGA (*n* = 277, comprising 241 HPV-negative and 36 HPV-positive cases), and GSE65858 cohorts (*n* = 211, 176 HPV-negative and 35 HPV-positive cases) were included. GSVA and subsequent k-means clustering with *k* = 3 delineated an additional MPS3 subgroup, significantly enriched in HPV-positive cases in the LMU-KKG and TCGA cohorts (Fisher’s exact test *P* < 0.001 for TCGA and LMU-KKG, and *P* = 0.138 for GSE65858). MPS3 showed the lowest overall metabolic GSVA enrichment scores (Fig. 5a). Identical metabolic profiles across the three MPS phenotypes were obtained by MPS subtyping analysis of the comprehensive TCGA data set comprising 500 cases^2^, and accordingly MPS3 was significantly enriched in HPV-positive cases (*P* < 0.001, Supplementary Fig. 10, including MSigDB hallmarks analysis). Since Chakravarthy et al.^23^ determined the HPV-status of the comprehensive TCGA cohort from RNAseq data exclusively, we focused on the harmonized TCGA collection for which the HPV-status was determined based on p16 immunohistochemistry in combination with HPV-specific DNA analysis, similar to the LMU-KKG cohort.

A random forest classifier trained on the gene expression data of the LMU-KKG cohort and tested on the TCGA cohort achieved prediction accuracy of 0.685 (95% CI 0.602-0.761). When applied to the exclusively HPV-positive LHSC OPC cohort (*n* = 43)^24^, it assigned *n* = 30 to MPS3, *n* = 12 to MPS2, and only one case to MPS1. This distribution mirrored findings in the LMU-KKG and TCGA cohorts (HPV-positive cases LMU-KKG: *n* = 31, *n* = 16, *n* = 12; TCGA: *n* = 13, *n* = 5, *n* = 3 classified as MPS3, MPS2, or MPS1, respectively). Accordingly, the LHSC OPC MPS3 subgroup exhibited consistently lower metabolic enrichment scores compared to MPS2 (Supplementary Fig. 11). These results independently confirm findings from LMU-KKG, TCGA, and GSE65858 cohorts (Fig. 5) and demonstrate the feasibility of MPS classification of individual tumors.

Upon inclusion of HPV-positive HNSCCs, associations with clinical parameters and molecular characteristics of MPS1 and MPS2 remained virtually unchanged (Fig. 5b, Supplementary Fig. 12). However, the delineation of the MPS3 phenotype complemented the picture of metabolic heterogeneity in HNSCC and contributed to a refined definition of the high-risk MPS1 phenotype (Fig. 5b). In terms of metabolic gene expression based on the KEGG metabolic pathways collection, MPS3 was highly similar to MPS1, with the clear exception of GAG metabolism. Specifically, CS/DS biosynthesis was detected as the only major difference between MPS1 and MPS3, suggesting that – from the metabolic perspective – CS/DS biosynthesis may contribute to the clear impaired clinical response of MPS1 vs. MPS3 and MPS2 (Fig. 5a, b). In contrast, the full transcriptomic landscape of MPS3 as assessed by GSVA with the MSigDB hallmarks collection and the gene expression signatures associated with the composition of the TME (see Fig. 3c) was more similar to MPS2 (Fig. 5c, d) with the exception of TIS and *PD-1 signaling* signatures. These results allow the conclusion that elevated GAG metabolism and specifically CS/DS biosynthesis are strongly associated with the establishment of an immunosuppressive TME in association with enrichment of CAFs, cancer cell stemness, and ECM remodeling, and might therefore contribute to explain the significantly impaired survival and therapy-induced tumor control of MPS1 HNSCCs. The recently published HPV-related tumor-specific NFκB-signature was not associated with the MPS classification (Supplementary Fig. 10c).

### Chondroitin/dermatan sulfate metabolism of malignant cells is associated with unfavorable outcome in HPV-negative HNSCC

Motivated by the finding that CS/DS metabolism was the most prominently upregulated metabolic pathway in MPS1 compared to MPS2 and MPS3 (Fig. 1b, d, Fig. 5a), we assessed its prognostic value by univariable Cox PH modeling. Elevated CS/DS pathway enrichment was strongly associated with an unfavorable adjuvant treatment response in HPV-negative cases of the LMU-KKG cohort (P.adj<0.05 for OS, RFS, LR-RFS, DSS, and FFR, Table 1b). The prognostic association of the CS/DS pathway enrichment and OS could be independently validated in the GSE65858 and GSE41613 HNSCC data sets (*P* = 0.004 and *P* = 0.005, respectively, Table 1b).

In addition, we investigated the tumor cell-specificity of the CS/DS metabolism in the scRNAseq data sets of Puram et al. and Kürten et al.^18,25^. The GSVA enrichment scores of the CS/DS pathway were significantly elevated in malignant cells of MPS1 compared to MPS2 malignant cells (*P* < 0.001, generalized linear mixed model) (Fig. 3g). An alternative computational approach identified MPS1 malignant cell clusters (Supplementary Fig. 13a) with a significant enrichment of the CS/DS pathway compared to MPS2 malignant cell clusters (Supplementary Fig. 13b), thus serving as an independent confirmation. Accordingly, MPS1-specific upregulation of the CS/DS pathway occurs in malignant cells and appears to be of prognostic relevance.

### Association of MPS phenotypes with histology and chondroitin sulfate proteoglycan (CSPG) immunostaining in HPV-negative HNSCC

**Fig. 6:**
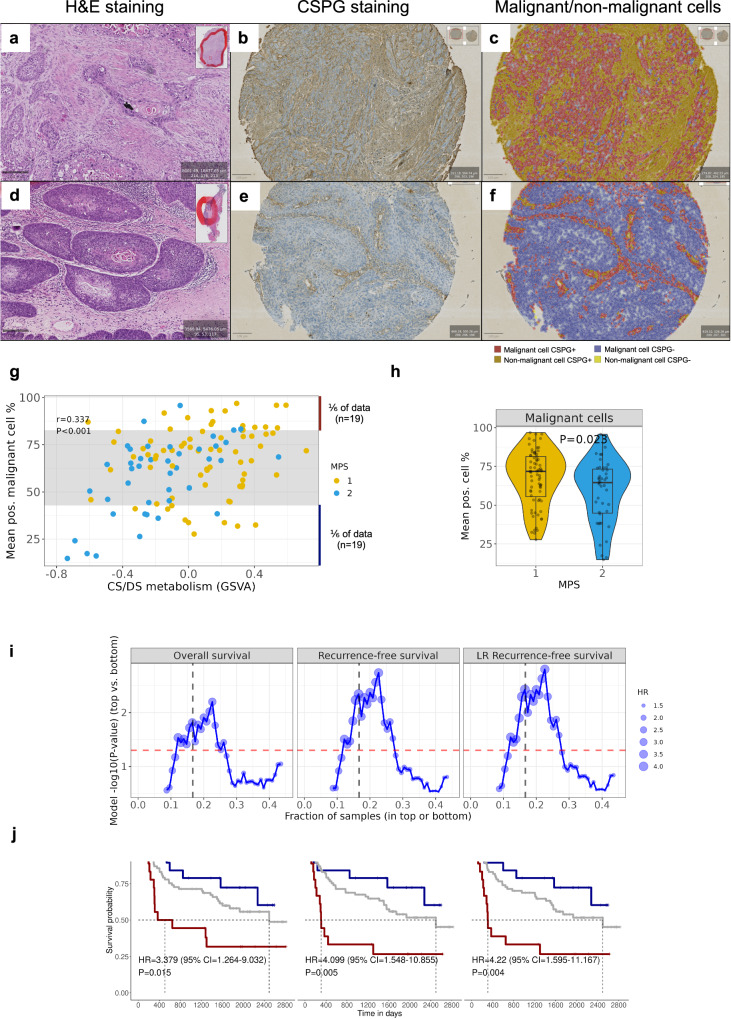
ECM remodeling, activated tumor stroma, and elevated chondroitin sulfate proteoglycan (CSPG) level in MPS1. HE sections of MPS1 (CS/DS metabolism high) and MPS2 (CS/DS metabolism low) tumors in the LMU-KKG cohort (6x magnification, 200 µm scale bar) (**a**, **d**). MPS1 (high CS/DS metabolism): activated stroma/ECM rearrangement (**a**). MPS2 (low CS/DS metabolism): lower level of malignant cells-TME interaction (**d**). CSPG staining in FFPE tissue sections of MPS1 and MPS2 tumors, respectively, from the LMU-KKG cohort (3x magnification, 100 µm scale bar) (**b**, **c**, **e**, **f**, additional examples in Supplementary Figs. IHC slides). Elevated fractions of CSPG-positive malignant cells in MPS1 (**b**, **c***)* compared to MPS2 malignant cells (**e**, **f**). Annotation color: red=pos. malignant cell, blue=neg. malignant cell, dark yellow=pos. non-malignant cell, light yellow=neg. non-malignant cell. Scatterplot with GSVA enrichment scores of CS/DS metabolism vs. CSPG-positive malignant cell % of 115 HPV-negative cases from LMU-KKG (Spearman *r* = 0.337, *P* < 0.001) (**g**). The gray interval area indicates the inner 2/3 of the data set. CSPG-positive cell fractions were calculated using QuPath. CSPG-positive malignant cell % difference of MPS1 and MPS2 of the same 115 cases (Wilcoxon *P* = 0.023) (**h**). Sliding threshold analysis for definition of top and bottom fraction of cases (based on mean CSPG-positive malignant cell %). Cox models’ HR (dot size), -log10(P-value) (y-axis) for the top vs bottom groups are plotted for varying thresholds (x-axis) (**i**). Dashed red lines indicate *P* = 0.05. Dashed black lines indicate the 1/6 fraction threshold for top vs. bottom, as visualized in panels **g** and in the KM plots in panel **j**. KM analysis of 114 HPV-negative cases from LMU-KKG (one patient did not have survival data), highlighting the top and bottom 1/6 of tumors (based on CSPG-positive malignant cell %) Cox models’ HR, CI, and *P*-value for top vs bottom groups (**j**). HR, 95% CI, and P indicate the comparison of the CSPG high and CSPG low groups.

Histopathologic evaluation of hematoxylin and eosin (HE)-stained tissue sections of HPV-negative tumors from the LMU-KKG and TCGA cohorts was carried out to compare MPS1- and MPS2-classified tumors. Two distinct histological tumor phenotypes were observed. HE sections of ten MPS1 tumors with the highest and ten MPS2 tumors with the lowest CS/DS metabolism pathway enrichment scores were examined (Supplementary Figs. HE slides). Representative examples of the histological phenotype of MPS1 tumors (CS/DS pathway enrichment scores high) showed an elevated degree of ECM remodeling and CAF activation as indicated by spindle-shaped fibroblasts (CAFs) with random orientation and a prominent cell nucleus (Fig. 6a, c). Furthermore, MPS1 was characterized by highly intermixed populations of malignant and stromal cells. In contrast, MPS2 (CS/DS pathway enrichment scores low) displayed well-separated populations of malignant and stromal cells with seemingly little interaction between malignant cells and fibroblasts. MPS2 tumors showed a clearly reduced degree of ECM remodeling, accompanied by a mature small spindle cell fibroblast morphology with a thin body structure and a symmetric and/or parallel orientation (Fig. 6b, d). These observations provided a histopathologic visualization of the RNA level-defined MPS1/MPS2 phenotypes and strengthened our transcriptome-based notion of changes in the TME contexture.

Our pilot MALDI-MSI experiments provided proof-of-concept data illustrating fundamental differences in the metabolite spectra of MPS1 and MPS2 HNSCCs (Supplementary Fig. MALDI-MSI HE). Given the specific enrichment of the CS/DS pathway in MPS1, its conceivable implications for clinical prognosis, and its accessibility for systematic immunohistochemical analyses on the basis of chondroitin sulfate proteoglycan (CSPG) staining, automated quantification of CSPG-positive malignant and non-malignant cell fractions was performed in the LMU-KKG cohort (*n* = 115) with QuPath^26^. The analysis revealed a significant correlation between CSPG-positive malignant cell fractions and GSVA CS/DS pathway enrichment scores (Fig. 6g, Spearman *r* = 0.337, *P* < 0.001). CSPG-positive malignant cell fractions were significantly elevated in MPS1 compared to MPS2 tumors (*P* = 0.023) (Fig. 6h) and strongly associated with clinical endpoints including RFS, LR-RFS, DSS, and FFR (*P* adj.<0.1) (Supplementary Table 15), thus, validating the association of CD/DS pathway expression with CSPG metabolite abundance and clinical outcome. KM analysis revealed significantly elevated risks when comparing the top 1/6 vs. bottom 1/6 of cases for the endpoints OS, RFS, LR-RFS, DSS, FFR (*P* < 0.05, HR > 3.3) and a strong tendency for LRC (*P* = 0.066) (Fig. 6j, Supplementary Fig. 14b). The inner 2/3 of the cases collectively exhibited an intermediate risk compared to the top and bottom 1/6 of the cases. Sliding threshold analysis for the selection of top and bottom fraction of the cases (based on CSPG-positivity) delineated a significant difference in clinical outcome between top and bottom groups in the range of 15–25% fractions of the data set (Fig. 6i, Supplementary Fig. 14a). These results complemented and validated the initially observed association between CS/DS pathway expression in malignant cells and unfavorable therapy outcome as derived from transcriptomic data (bulk RNAseq and scRNAseq) at the metabolite level and demonstrated a potential clinical relevance of CSPG-positive malignant cell fractions in HNSCC.

### Replication of MPS phenotypes in established HNSCC cell lines and orthotopic xenotransplants

**Fig. 7:**
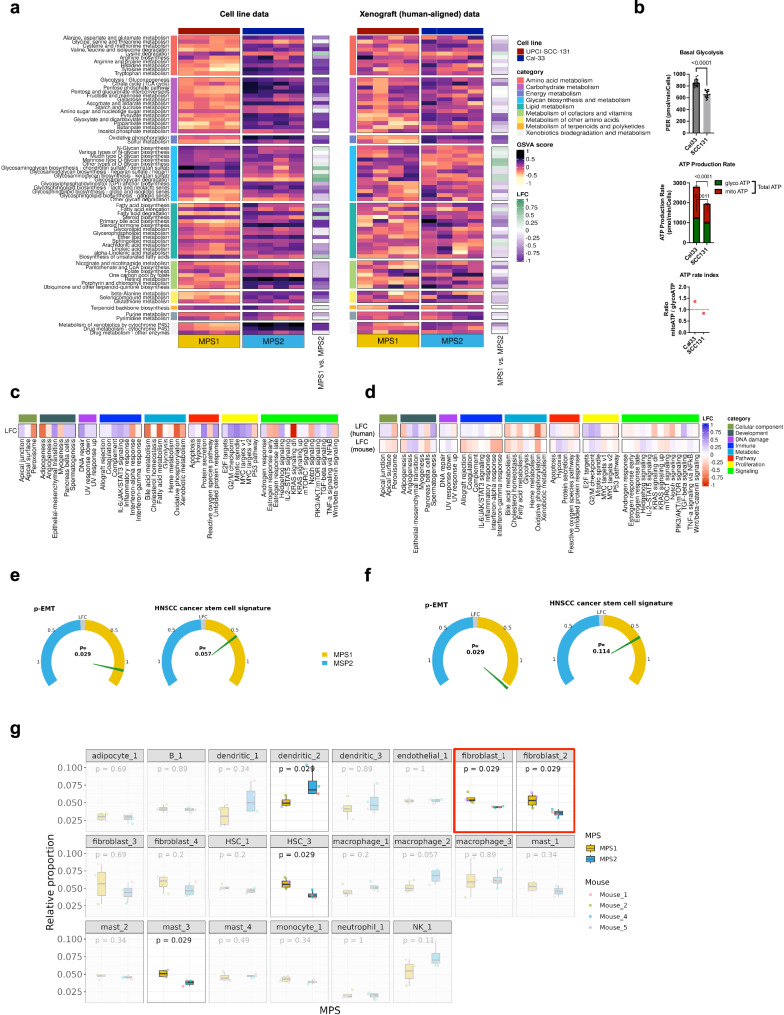
In vitro and orthotopic xenograft models support MPS-specific TME assembly. KEGG metabolic GSVA scores of UPCI-SCC-131 and Cal33 HNSCC cell lines (each *n* = 4 replicates) were used for MPS-classification using NSC trained/tested on the LMU-KKG/TCGA HPV-negative cohort (**a**). Proton Efflux Rate (PER) representing glycolytic rate in Cal33 and UPCI-SCC131 cells determined with a Glycolysis Rate Assay using a Seahorse XFe96 Analyzer and basal glycolysis by Glycolytic rate assay (*n* = 7 wells each). Total ATP rate, ATP by glycolysis (glyco) and mitochondrial (mito) production in Cal33 by ATP Rate Assay. ATP rate index representing the ratio of ATP production by mitochondria and glycolysis for Cal33 and UPCI-SCC131 (**b**). LFC values of GSVA MSigDB hallmarks scores of MPS1 vs. MPS2 (**c**). Gene signatures (*p-EMT*, *HNSCC cancer stem cell signature*) were quantified likewise and compared between MPS1 vs. MPS2 (**e**). UPCI-SCC-131 and Cal33 xenografts were MPS classified (using RNAseq data thereof and NSC), and human-aligned data (tumor cells) KEGG metabolic GSVA scores were visualized (**a**). Using human and mouse genome-aligned (host cells, TME) data, hallmarks GSVA scores were compared MPS1 vs. MPS2 (**d**), and accordingly, human-aligned gene signatures were compared between MPSs (**f**). Mouse genome-aligned gene expression data were utilized in cell type deconvolution of the TME using the SSMD tool, and relative proportions of cell types were compared between MPS1 and MPS2 (**g**). LFC log2 fold change, HSC hematopoietic stem cell.

To enable future functional and mechanistic analyses, preclinical MPS models are needed. We therefore analyzed metabolic gene expression in established HNSCC cell lines and orthotopic xenotransplants derived thereof, and successfully identified the MPS1 and MPS2 phenotypes in UPCI-SCC-131 and Cal33, respectively. Cell lines and xenotransplants exhibited the corresponding KEGG metabolic pathways profiles with similar characteristics as described for clinical tumor samples, including the MPS1-specific upregulation of GAG metabolism pathways (Fig. 7a). Transcriptomic profiles (MSigDB hallmarks and other gene signatures Fig. 7c–f) recapitulated patient-derived observations, most prominently the elevated enrichment scores of *EMT*/*p-EMT*, *angiogenesis*, *cancer stemness* signatures.

Additionally, the attenuated energy metabolism of the MPS1 phenotype as found in clinical cohorts could be validated via real-time functional measurement of bioenergetic pathways using a Seahorse Bioanalyzer. UPCI-SCC-131 cells exhibited significantly reduced basal/compensatory glycolytic potential and ATP production rates of mitochondrial origin (oxidative phosphorylation) and via glycolysis (Fig. 7b). Furthermore, the altered mitotic/glycolytic ATP production ratio (Fig. 7b) and different capacities/dependencies to oxidize varying fuels, including glucose, glutamine, and fatty acids (Supplementary Fig. Fuel Flex Test), support the interpretation of a reduced/decelerated energy metabolism in favor of GAG-related metabolic processes.

From our analyses in HNSCC tumor cohorts, we deduced an MPS-specific and tumor cell-driven TME assembly. Along these lines, the deconvolution of the mouse genome-aligned xenograft RNAseq data (reflecting the mouse TME) confirmed a tumor cell-driven, MPS-specific TME contexture. Our data indicated differential abundance of specific cell types within the TME of orthotopic MPS1 and MPS2 xenografts with a particular enrichment of different fibroblast subsets in MPS1 (Fig. 7g, cell type marker genes in Supplementary Table 16). Concordantly, the MPS-specific tumor morphology, which we observed in the patient-derived tissue sections (Fig. 6a, Supplementary Figs. HE slides), was present in the respective xenograft MPS phenotype model (Supplementary Figure Xenograft HE). These findings encourage the conclusion that MPS1 malignant cells with elevated GAG metabolism and associated signaling promote the recruitment, proliferation, and/or differentiation of specific fibroblast subsets needed for the TME reorganization and MPS1-specific tumor-stroma interactions.

## Discussion

Metabolic dysregulation contributes to molecular heterogeneity and diverging therapy responses and therefore represents a major challenge in tumor therapy. For various cancer types, alterations in tumor metabolism and specific metabolic phenotypes have been linked to clinical prognosis^8,27,28^. Although measurement of metabolite abundance is classically considered to constitute the gold-standard for metabolic profiling, metabolic phenotypes can be determined on the basis of transcriptomic data, rendering this approach particularly attractive and advantageous for application in large cohorts of clinical samples where metabolite level data are limited or not available^8^. We provide a reproducible and systematic workflow to perform this approach within a publicly available R library (*MetabolicExpressR*).

In the present study, we made use of bulk RNAseq and microarray data from four independent clinical HNSCC cohorts and two independent single-cell RNAseq data sets and identified metabolic pathway-based subtypes (MPS) in HNSCC: two MPS subtypes of HPV-negative cases (MPS1 and MPS2) and one MPS subtype that was enriched in HPV-positive cases (MPS3). Compared to MPS2, MPS1 showed broad-range downregulation of metabolism-associated gene expression – except for pathways of glycan metabolism – and was consistently associated with impaired prognosis in terms of various clinical endpoints. Interestingly, MPS3, the subtype enriched in HPV-positive cases, despite being of very different etiology, pathogenesis, and prognosis^29^, on the level of metabolic gene expression was highly similar to MPS1 – again except for pathways of glycan metabolism. Accordingly, increased expression of glycan metabolism genes was uniquely associated with MPS1 and compromised clinical outcome.

In relation to the established molecular “Keck classification”^10^, the MPS subgroups showed significantly different compositions and enrichment of specific Keck subtypes. However, for the “Keck classification”, no significant prognostic relevance was observed for any clinical endpoint tested, neither in the herein analyzed LMU-KKG cohort nor in the cohort of Keck et al.^10^. Thus, MPS phenotypes have superior prognostic power compared to Keck subtypes regarding clinical outcome after adjuvant radio(chemo)therapy.

The clinically most interesting, because prognostically most challenging phenotype MPS1 was uniquely and consistently associated with increased expression of GAG metabolism genes across all tested cohorts, in particular genes of the CS/DS metabolism pathway. GAGs are long, linear polysaccharides that form proteoglycan molecules^16^, which along with glycolipids and glycoproteins, are components of the cellular glycocalyx and the ECM. ECM and glycocalyx together form a complex network that impacts various biological processes, including regulation of cell adhesion, cell migration, and cell signaling in physiological and pathological conditions, such as metabolic and neurodegenerative diseases, infections, and cancer^16^. Glycobiology research has gained increasing attention in translational oncology, and several studies highlighted its importance in biological processes related to tumor control and therapy success^30^. However, the functional implications and the corresponding association with clinical outcome appear to vary between different tumor entities. In the present study, the upregulated expression of CS/DS metabolism pathway showed a clear and coherent association in continuous Cox PH models with different clinical endpoints for survival and loco-regional control in various clinical HNSCC cohorts. Moreover, our findings derived from the mRNA level could be validated and related to cellular structures within the histological context on the metabolite level by immunohistochemistry of chondroitin sulfate proteoglycans (CSPGs) and pilot MALDI-MSI analyses of chondroitin sulfate-related m/z species in the LMU-KKG cohort. The histological analysis revealed that higher abundance of CSPG-positive malignant cells within a tumor was associated with adverse clinical outcome upon adjuvant radio(chemo)therapy. Furthermore, our IHC findings suggested a potential use of CSPG as a prognostic marker and pointed to a potential impact on the tumor microenvironment (TME). Previous studies have associated the CS/DS metabolism with tumorigenic properties, ECM remodeling, and immune exclusion in esophageal squamous cell carcinoma, pancreatic adenocarcinoma, and colon carcinoma^15,31,32^. While in those studies it remained unclear whether CS/DS proteoglycans derive from malignant cells or CAFs, our results clearly showed a tumor cell-specific synthesis of chondroitin sulfate and CSPG expression in HPV-negative HNSCC.

Regarding its global transcriptomic landscape in comparison to MPS2 and MPS3, the MPS1 phenotype exhibited several distinct molecular individualities that are indicative of tumor progression and increased aggressiveness. Although detailed mechanisms and interplay of metabolic dysregulation and other tumor-specific processes are not fully understood, the presented molecular characterization, including the hypothesis-driven quantification of several specific gene signatures and the exploratory GSEA enrichmentMap analysis (GO Biological Processes compilation) (Fig. 4), supports the assumption of a close relationship between changes in the TME and MPS1-associated molecular processes. This assumption is further strengthened by the differences in TME contexture of MPS1 vs. MPS2 observed in patient tumors and the xenograft models (Fig. 7). Our xenograft observations suggest that the MPS phenotype of the tumor cells plays a driving role in shaping the TME. The upregulation of *TGF-β signaling* in MPS1 tumors points towards activation of CAFs, ECM remodeling, and immunosuppression, as previously reported in several cancer types including HNSCC^33^. Elevated expression of the HNSCC-specific *stemness signature* indicates increased abundance of stem-like cancer cells which have been shown to promote metastasis formation and to contribute to increased resistance to radio(chemo)therapy and tumor relapse^34^. Despite elevated expression of immune-related hallmark gene sets and the tumor-inflammation-signature (TIS), the higher level of *PD-1 signaling* potentially indicates immunosuppressive processes in MPS1 tumors and promotes the exhaustion of activated T-cells^35^.

For a more detailed understanding of an altered TME composition we focused on CAF subtype analysis based on the specific signatures proposed by Galbo et al.^36^, which showed an association of MPS1 tumors – with elevated and malignant cell-specific GAG metabolism – and increased CAF activities across all CAF subtypes examined. CAFs have varying promoting or suppressing roles in tumor development depending on the specific CAF subtype and cancer type^36,37^. In HNSCC, specific CAF subtypes play a crucial role in ECM remodeling, which is reflected by their morphological features and has been linked to compromised therapy responses^37^. Histologically, we observed this characteristic fibroblast and tumor stroma morphology predominantly in MPS1-classified tumors of the LMU-KKG and TCGA cohorts as well as in the MPS1 xenograft model (Supplementary Figure Xenograft HE). The immature, plump, and spindle-shaped CAFs with random orientation and prominent nuclei that were found in MPS1 tumors are considered to contribute to increased microvessel density and (p-)EMT^38,39^.

Beyond their potential role in prognosis, glycan metabolism and its GAG products offer promising avenues to new therapeutic approaches due to their favorable physicochemical properties and tumor-specific sulfation patterns^40^. Direct targeting and/or utilization of GAGs in drug delivery may hold great potential for therapy of HPV-negative MPS1 HNSCCs^30^, where combinatorial treatment regimes, including immuno- and chemotherapy so far failed to improve therapy success for the majority of patients^41^.

While this study marks the initial exploration of metabolic pathway-based subtypes in HNSCC, it has certain limitations. Firstly, clinical endpoints were not consistently available for all analyzed cohorts, for instance, the TCGA cohort only provided overall survival (OS) data. Secondly, the availability of single-cell RNAseq data was limited to a specific set of published cases, restricting cell type-specific analyses. Thirdly, the conclusions drawn from proof-of-concept experiments, such as xenograft models and metabolite measurements, necessitate comprehensive follow-up studies for a more thorough understanding. Nevertheless, this study dedicated substantial efforts to validate the identified MPS phenotypes and thoroughly describe their molecular landscapes.

In conclusion, we identified metabolic pathway-based subtypes in HNSCC of which MPS1 turned out to be the clinically most interesting and challenging phenotype due its adverse clinical outcome and its distinct transcriptomics landscape, histology, and TME composition, signifying a noteworthy subgroup within HPV-negative HNSCC for the investigation of alternative therapeutic approaches. Elevated expression levels of glycosaminoglycan metabolism and increased abundance of metabolites derived thereof were associated with impaired prognosis and may represent potential clinical prognosticators and/or future therapeutic targets in HPV-negative HNSCC upon further investigation, for instance with the herein presented model systems.

## Methods

### Patient specimens and study design

In this study, we utilized our “in-house” collected clinical cohort with head and neck squamous cell carcinoma (HNSCC) patients who underwent surgery followed by additional radio(chemo)therapy. These groups are known as the LMU-KKG 08-13 and LMU-KKG 13-16 cohorts, which were collected by the Ludwig-Maximilians-University of Munich, Clinical Cooperation Group “Personalized Radiotherapy in Head and Neck Cancer”. The LMU-KKG 08-13 cohort’s patient specimens and study design were previously described by Hess et al.^42^. The LMU-KKG 13-16 cohort involved the retrospective collection of clinical data and treatment-naive patient tissue specimens. All patients in both cohorts were diagnosed with HNSCC in the hypopharynx, oropharynx, or oral cavity, confirmed through histological examination. The retrospective study was conducted in accordance with the Declaration of Helsinki and received ethical approval from the LMU’s ethics committee (EA 312-12, 448-13, 17-116). A written informed consent was obtained from all human participants. The tumor stage was determined using the AJCC 7th edition^43^ of the Union for International Cancer Control Tumor-Node-Metastasis (UICC TNM) Classification of Malignant Tumors^43^. Human papillomavirus (HPV) status was assessed using p16^INK4a^ immunohistochemistry in combination with HPV DNA detection, following a previously described method^44^. The LMU-KKG 13-16 study, conducted at a single center, initially included HNSCC patients with at least UICC TNM stage III or close/positive microscopic resection margins. Close margins were defined as R0 but less than 5 mm according to the local pathologist. These patients received adjuvant radiotherapy between 2013 and 2016 at the LMU Department of Radiation Oncology. The treatment duration had a median of 45 days (interquartile range [IQR]: 43–47 days) with five treatment fractions per week. The median radiation dose applied was 64 Gy (median dose of 2 Gy per fraction) to the former tumor site or regions with extracapsular extension (ECE). Additionally, elective lymph node regions were irradiated with a median dose of 50 Gy (median dose of 2 Gy per fraction), and involved lymph node regions received a median dose of 56 Gy (median dose of 2 Gy per fraction), based on tumor stage and location. Patients with close/positive microscopic resection margins and/or ECE received concurrent chemotherapy. Among the total 134 patients, 49% received chemotherapy: 42% received CDDP/5-fluorouracil (CDDP: 20 mg/m2 BSA on days 1-5/29-33; 5-FU: 600 mg/m2 BSA on days 1-5/29-33), and for 9% of the patients, Mitomycin C (MMC) or 5-FU/MMC replaced platinum-based chemotherapy. After reviewing hematoxylin and eosin-stained tissue sections from available blocks with formalin-fixed and paraffin-embedded (FFPE) tumor tissue, a pathologist (A. Walch) annotated the tumor area. If necessary, microdissection was performed prior to nucleic acid extraction to ensure a minimum tumor cellularity of 60% (median 70%, IQR: 70%-80%). Definitions of clinical endpoints for LMU-KKG as previously published by Hess et al.^42^. In brief, recurrence-free survival (RFS) and locoregional recurrence-free survival were defined as the time (days) from radiotherapy start to the first observation of a distant or locoregional recurrence, respectively, or death of the patient. Freedom from recurrence (FFR) was defined as the time (days) from the start of radiotherapy to the first locoregional or distant recurrence. Additionally, overall survival (OS), disease-specific survival (DSS), and locoregional control (LRC) were calculated as the time in days.

### Full-length and 3’ mRNA sequencing

Processing of tumor specimens was performed as outlined in Hess et al.^42^. Transcriptomic quantification of clinical tumor samples was carried out by mRNA sequencing. Nucleic acid extraction, mRNAseq library preparation, whole RNA sequencing, and data preprocessing of the LMU-KKG 08-13 cohort (*n* = 70) was executed as described previously^9^.

Data generation for the LMU-KKG 13-16 (*n* = 134) cohort was realized from FFPE tissue sections. Total RNA has been extracted and quality checked as for whole RNAseq. 3’ tag libraries were generated from 100 ng input using the QuantSeq 3’-RNA-Seq Library Prep Kit FWD for Illumina with i5 6 nt Dual Indexing (Lexogen GmbH, Vienna, Austria) according to the manufacturer’s instructions for dual-indexing and low quantity samples. For library amplification, PCR cycles were determined using the PCR Add-on Kit for Illumina (Lexogen). Quantity and quality of sequencing libraries were assessed using the Quanti-iT PicoGreen dsDNA Assay Kit (ThermoScientific) and the Bioanalyzer High Sensitivity DNA Analysis Kit (Agilent Technologies). An equimolar pool of libraries has been prepared and sequenced in 150 bp paired-end mode at Novogene.

### KEGG metabolic pathways collection

The KEGG metabolic pathways collection with 95 pathways was obtained from the official KEGG website using a custom R script (accessed on 12.10.2021, Supplementary Table 17)^45,46^. These metabolic pathways consist of 1659 unique genes that can be grouped into 12 main categories. These categories are amino acid metabolism, biosynthesis of other secondary metabolites, carbohydrate metabolism, energy metabolism, glycan biosynthesis and metabolism, lipid metabolism, metabolism of cofactors and vitamins, metabolism of other amino acids, metabolism of terpenoids and polyketides, not included in regular maps, nucleotide metabolism, xenobiotics biodegradation, and metabolism.

### Bulk RNA sequencing data analysis

RNAseq data of LMU-KKG 13-16 cohort were pre-processed by adapter trimming (BBDuk), alignment to human GRCh38.93 reference genome (STAR^47^), gene expression quantification (HTSeq-count^48^), and imported into R (DESeq2^49^). Differential gene expression analyses are based on count data and the DESeq2 workflow. Processed, DESeq2 normalized count data of 43 HPV-positive oropharyngeal cancer (OPC) cases were provided by Anthony C. Nichols (Western University, London, Ontario, Canada) and processed as described previously^24^.

Transcriptomic data of *n* = 277 primary HNSCC tumors (including 77 adjuvantly treated tumors) and 37 matched normal tissues from the TCGA cohort (harmonized collection, accessed on 11.07.2022) were obtained through the TCGAbiolinks R library^50–52^.

### Publicly available gene and protein expression data

Processed and normalized gene and protein (z-scores) expression data of the CPTAC-HNSCC cohort^13^ consisting of 108 primary tumors and 53 (and 62 for protein expression data) matched adjacent normal cases were downloaded from the LinkedOmics website (http://linkedomics.org/data_download/CPTAC-HNSCC/, accessed on 17.05.2023). Publicly available, processed and normalized gene expression data sets from two independent HNSCC cohorts were obtained from Gene Expression Onmibus (GEO, GSE65858 and GSE41613)^53,54^. GSE65858 HPV-negative (*n* = 176) and HPV-positive (*n* = 35), and GSE41613 HPV-negative (*n* = 96) primary tumors were considered in the downstream analysis.

Differential gene expression analysis between groups of the GSE65858 and GSE41613 data sets was performed following the standard limma pipeline^55^.

### Single-cell RNAseq analysis

Processed single-cell RNAseq (scRNAseq) expression data (log_2_(TPM/10 + 1) expression values) from Puram et al.^18^ were obtained from GEO (GSE103322). For all analyses, we used cells from ten HPV-negative patients with sufficiently high cell numbers (patients HNSCC5, HNSCC6, HNSCC16, HNSCC17, HNSCC18, HNSCC20, HNSCC22, HNSCC28, HNSCC26, HNSCC25, based on Puram et al.^18^). Primary tumor malignant (*n* = 1313) and fibroblast cells (*n* = 678) were considered in the downstream analysis. Processed expression data of fibroblast and malignant cells were loaded separately into R and further analyzed using the R library Seurat^56^, and a neighborhood graph was constructed to identify related groups of cells using the first ten dimensions of reduction to use as input. Louvain clustering was then performed on the neighborhood graph with the resolution parameter set to 0.5 and data visualization by t-SNE plots using the first ten dimensions (Supplementary Fig. 15, 16). Cell types (cancer-associated fibroblast (CAF), myofibroblast, and normal fibroblast) were assigned to fibroblast clusters based on the expressed marker genes from Puram et al.

Processed gene barcodes of the Kürten et al.^25^ data set were downloaded from GEO (GSE164690). Data of CD45-negative cells (*n* = 21735) from 9 HPV-negative patients were loaded into R using Seurat. Quality control, filtering, normalization, and dimensionality reduction of data were done as in Kürten et al.^25^. Clustering of all CD45-negative cells was done as outlined above, and major cell types were assigned to clusters based on top-expressed known marker genes, such as epithelial cells (*KRT6A*, *KRT14*, *KRT15*, *KRT17*, *S100A2*), fibroblasts (*COL1A1*, *COL3A1*, *LUM*, *DCN*, *MMP1*), endothelial cells (VWF, *ACKR1*, *SPARCL1*), or immune cells (*GNLY*, *GZMA*, *CXCR4*, *CD3D*, *NKG7*, *LST1*, *AIF1*, *LYZ*). Similarly, sub-clustering of epithelial (*n* = 4190) and fibroblast (*n* = 4130) cells was performed. Fibroblast cell clusters were annotated based on marker genes from Kürten et al., annotating clusters as CAFs, myofibroblasts, or normal fibroblasts. The data were visualized as t-SNE plots (Supplementary Fig. 16). InferCNV^57^ was used to differentiate between malignant and non-malignant epithelial cells. Expression data of the sub-clustered epithelial cells, and peripheral blood leukocytes (PBL) as reference cells were used to infer copy number variation (CNV) from scRNAseq data. Patterns of the epithelial cells were compared to the baseline signal (PBL) to determine if the epithelial cell clusters are malignant or non-malignant (Supplementary Fig. 17).

Gene Set Variation Analysis (GSVA) of selected Kyoto Encyclopedia of Genes and Genomes (KEGG)^45,46^ metabolic pathways and gene signatures enrichment scores were compared between groups of the Puram et al. data set using generalized linear mixed models (GLMM) with patient-specific mixed effects. The lmer function from the R libraries lme4 and lmerTest^58,59^ were used for the calculations, setting the GSVA enrichment score as the response variable, group as the independent variable, and setting the mixed effect on the patient variable.

Differential gene set enrichment analysis (GSEA) was performed between the MPS1 and MPS2 malignant cell clusters of the Puram et al. data set using the Seurat function DEenrichRPlot with the MSigDB hallmarks.

To perform GSEA on the Kürten et al. scRNAseq data set, we used the R library singleseqgset^60^ and the KEGG metabolic pathways. We applied this analysis to the epithelial and CAF cell clusters of the Kürten et al. expression data. Scaled and log-normalized data were used as input, and log fold change was calculated between cell clusters (one cluster versus the rest in the case of the epithelial cells, or one cluster versus another cluster in the case of the malignant and CAF group comparisons). Then, enrichment scores and *P*-values were calculated quantifying the enrichment of metabolic pathways per cluster. *P*-values of enrichment scores were adjusted for multiple testing (Benjamini-Hochberg correction). The metabolic pathway-based subtype (MPS) class of malignant cell clusters of the Kürten et al. data were defined based on the Spearman correlation test of enrichment scores to TCGA and LMU-KKG MPS class GSVA centroids (correlation test *P* adj.<0.05 for both cohorts).

### Quantification of metabolic pathways and prognostic gene signatures

To quantify the enrichment of the KEGG metabolic pathways collection^45,46^ at sample-level separately in each data set, we applied GSVA using the GSVA R library^61^. For all data sets, DESeq2 normalized counts were used as input for GSVA with the ‘kcdf = “Poisson”’ parameter, setting the minimal gene set size to 9 and maximal gene set size to 300. For the Puram et al. scRNAseq data set, the original processed data was used and GSVA was run with the kcdf = “Gaussian” parameter. Selected and published prognostic gene signatures include *partial epithelial-mesenchymal transition* (*p-EMT*)^18^, *tumor inflammation signature* (*TIS*)^62^, *CAF signatures*^36^, *PD-1 signaling* from the Reactome collection^35,63,64^, and *HNSCC cancer stem cell (CSC) signature*^65^. *p-EMT*, *TIS*, *HNSCC CSC*, *CAF signatures*, and *PD-1 signaling* were quantified using GSVA from gene expression data. Likewise, the HPV-related tumor-specific NFκB-signature (“skyblue1 module”) was quantified using GSVA in HPV-positive tumors analyzed in this study^4^.

PROGENy (Pathway RespOnsive GENes for activity inference)^66^ was used for sample-level quantification of eleven signaling pathways relevant in cancer. VST (Variance Stabilized Transformation) or log2 (GSE65858 and GSE41613) normalized expression values were used as input and the resulting matrix was mean-centered and scaled. Differential pathway activity between the two sample groups (MPS) was calculated by setting up linear models for each pathway with pathway activity as the independent variable and MPS class as the response variable.

### Pooling of the LMU-KKG 08-13 and LMU-KKG 13-16 data sets GSVA data

We merged the GSVA matrices of the LMU-KKG 08-13 (*n* = 70) and LMU 13-16 (*n* = 134) data sets due to their identical origin, treatment plans, and clinical follow-up data/endpoints and based on our observation that there is no data set-related batch effect when performing the PCA on the two cohorts’ metabolic pathways GSVA enrichment scores (Supplementary Fig. 18). We refer to this merged data set as the LMU-KKG cohort (*n* = 204). In any other analysis when gene expression data were used directly (CIBERSORTx, differential gene expression analysis, Keck transcriptional subtyping) the two LMU-KKG data sets were handled separately.

### Identification of metabolic subtypes from RNAseq data of HNSCC samples

GSVA enrichment scores of the KEGG metabolic pathways were subjected to k-means clustering, individually for each cohort. The optimal number of clusters (k) was determined based on the consensus of 22 indices from the NBClust R library^67^. These indices are: “kl”, “ch”, “hartigan”, “db”, “silhouette”, “duda”, “pseudot2”, “beale”, “ratkowsky”, “ball”, “ptbiserial”, “gap”, “frey”, “mcclain”, “gamma”, “gplus”, “tau”, “dunn”, “hubert”, “sdindex”, “dindex”, and “sdbw”. For NBClust, the minimum number of clusters was set to 2 and the maximum number of clusters to 10, and the cluster analysis method to “kmeans”. K-means clustering was performed using the function kmeans from the R library stats^68^ with the optimal k parameter defined as described above, with 100 iterations (iter.max parameter) and 100 random sets (nstart parameter). The MPS class for each sample was defined by the obtained k-means cluster membership.

The nearest shrunken centroids (NSC)^69^ classification was used to a) demonstrate the consistency of the MPS classes between the LMU-KKG and TCGA HPV-negative cohorts, b) and to show the malignant cell-specific metabolic enrichment of the MPSs. The patient-level GSVA metabolic enrichment scores were used for building an NSC classifier using the pamr.train function from the pamr R library^70^. The pamr.cv function was used from the same R library for finding the optimal threshold for predicting the MPS class using the nearest shrunken centroid fit. For MPS class prediction on sample-level GSVA metabolic enrichment scores with the trained NSC classifier, the pamr.predict function was used from the pamr R library with the optimal threshold value described above. In the case of the NSC classifications on the LMU-KKG, TCGA, and Puram et al. cohorts, the NSC classifier-predicted MPS class was compared to the k-means clustering-based MPS class and was visualized as confusion matrices. The consistent classification rate was calculated as the sum of consistent classifications divided by the total number of cases.

MPS subtyping of the Puram et al. malignant cells data on KEGG metabolic pathways enrichment scores was carried out according to the workflow of clinical cohorts presented in Fig. 1a.

### Transcriptional subtype classification of the patients

Transcriptional subtyping according to Keck and colleagues (“Keck subtypes”) of all HNSCC patients was performed on the HPV-negative and HPV-positive cases together as reported by Weber et al.^9^.

### Univariable and multivariable survival models, statistical data analysis

Summary statistics of the HNSCC HPV-negative subsets of the LMU-KKG, TCGA, GSE65858, and GSE41613 cohorts are shown in (Supplementary Table 1).

Prognostic relevance of the MPS classification was assessed by Cox Proportional-Hazards (Cox PH) and Kaplan-Meier (KM) analysis using the *survival* and *survminer* R libraries^71–73^. The proportional-hazards assumption of Cox regression models was assessed using the R function cox.zph() from the R library *survival*, which tests the independence of Schoenfeld residuals of the model covariates and time. Overall survival (OS), recurrence-free survival (RFS), freedom from recurrence (FFR), disease-specific survival (DSS), locoregional control (LRC), and locoregional recurrence-free survival (LR-RFS) endpoints were considered in the LMU-KKG cohort, while only OS was available for TCGA. Nominal logrank test *P*-value and HR with upper/lower band of the 95% confidence interval (CI) were reported. In the case of multivariable model comparisons, 85% CI of concordance-index (C-index) was indicated additionally, since this CI level reflects approximately a 5% error rate when comparing C-indices with CIs^74^. For multivariable Cox PH modeling, 12 pre-selected clinically prognostic factors in HNSCC in addition to MPS class were considered. These are sex, tumor localization, smoking status, chemotherapy (yes or no), grading (dichotomized), ECE, TNM T stage (dichotomized), TNM N stage (dichotomized), UICC stage (dichotomized), Resection margin status, LVI stage (lymphovascular invasion), PNI stage (perineural invasion).

For each survival endpoint (OS, DSS, RFS, FFR, LR-RFS, and LC) we performed a balanced random 7:3 train-test split of the LMU-KKG HPV-negative cohort using the createDataPartition function from the caret R library^75^. Then we tested each of these variables in univariable Cox PH models using the coxph function from the R library survival^71^ and kept variables with *P*-value < 0.2 for backward selection. Backward variable selection was done using the stepAIC function from the MASS R library^76^ with the *k* = 2 parameter.

A final multivariable model was built with the selected variables on the train test, risk score prediction was done on the test set, and C-index was reported using the concordance.index function from the R library survcomp^77,78^. Patient-level predicted and standardized (scaled, centered) risk score was used for setting up univariable models on the test set, and HR, 95% CI of the HR, and *P*-value were reported. These performance metrics were compared to those obtained from the resulting models without including the MPS class from the beginning of the modeling workflow.

The two-group Cox PH models (based solely on a dichotomous clinical variable) vs. four-group Cox PH models (based on a dichotomous clinical variable plus MPS) were tested by chi-square test.

Differential pathway enrichment between MPS classes for KEGG metabolic pathways^79,80^, MSigDB hallmark gene sets^81^, and the selected gene signatures were assessed by Wilcoxon rank-sum test. Differential testing of protein expression z-scores between groups was performed using the metric Cohen’s d^82^. Pre-ranked GSEA between groups of samples using proteins sorted by Cohen’s d was performed with the R library fgsea^83^.

Single cell-level pathway or gene signature enrichment scores comparison between groups of the Puram et al. scRNAseq data set^18^ was done using generalized linear mixed models with patient-specific random effects. In the case of the Kürten et al. scRNAseq data set^25^, pathway enrichment comparison between groups was performed using pre-ranked gene set enrichment analysis. Association tests were performed by two-sided Fisher’s exact test and multiple testing corrections by Benjamini-Hochberg *P*-value adjustment.

### Gene expression rule-based MPS classification of LHSC OPC HPV-positive tumors

A single sample pair-based (rule-based) random forest classifier was built using gene expression data of the LMU-KKG (*n* = 145) and TCGA (*n* = 277) cohorts, and the R library multiclassPairs^84^. This way, we were able to perform accurate MPS classification of tumors from the LHSC OPC cohort, consisting of only HPV-positive cases. Using the expression data of the LMU-KKG and TCGA cohorts, a random train-test (0.7:0.3) split was applied, genes were sorted using the sort_genes_RF function. Then, the parameters of the random forest model were optimized using the optimize_RF function. Finally, the random forest classifier was trained using the function train_RF on the training data set. MPS class was predicted on the test set, and performance was assessed by comparing predicted class labels to GSVA and k-means clustering-based MPS classes. Using the trained random forest classifier, the MPS class was predicted on the LHSC OPC HPV-positive data set gene expression data.

### CIBERSORTx cell type deconvolution

The latest version of CIBERSORTx fractions from docker (created on 2020-04-04)^85^ was used to calculate individual-level cell type fractions using the Puram et al. reference matrix (500 permutations in batch correction “B-mode”). For CIBERSORTx analysis, TPM expression data in the case of the LMU-KKG 08-13 and TCGA data sets, and DESeq2 normalized counts data for the LMU-KKG 13-16 cohort were used.

### Immunohistochemistry and image analysis

Immunohistochemical staining of FFPE tumor tissue sections (3 µm) was performed using a primary antibody against the glycosaminoglycan (GAG) portion of native chondroitin sulfate proteoglycan (CSPG) (Anti-Chondroitin Sulfate antibody [CS-56] (ab11570), Abcam, USA). The primary antibody was used at a dilution of 1:200 and applied to the sample using the automated staining instrument Discovery XT (Roche, Ventana, Tucson, AZ, USA). For detection, the secondary antibody Discovery-Universal (Ventana) was used. Signal detection was performed using peroxidase-DAB-(diaminobenzidine)-MAP chemistry (Roche, Ventana). The stained tissue sections were fixed in an ethanol series and coated by a coverslip before scanning at 20x objective magnification with a digital slide scanning system (Axioscan 7, Carl Zeiss MicroImaging, Jena, Germany). The specificity and quality of the staining were reviewed and confirmed by a pathologist (A. Walch).

The open-source software QuPath^26^ was used to quantify the fraction of CSPG-positive malignant cells from IHC tissue microarray (TMA) core images of the HPV-negative LMU-KKG cohort (*n* = 115).

Following positive cell detection and cell type annotation (malignant or non-malignant) on two MPS1 (CS/DS GSVA enrichment high) and two MPS2 (CS/DS GSVA enrichment low) cores, a random forest (RTrees) cell-type classifier was trained on these four manually annotated cores and all the remaining cores were annotated after positive cell detection. Positive cell detection was done via the optical density sum detection image parameter, all the other options were kept default. This way, CSPG positive cell % per malignant and non-malignant cells were quantified in TMA cores of 115 tumors, each of which was represented by three cores on the TMA. For further analysis, the average positive cell fraction [%] (malignant or non-malignant) per tumor (three cores) was considered.

### Cell line and xenograft data generation and processing

HNSCC cell lines were purchased from DSMZ (Braunschweig, Germany), authenticated by short tandem repeat (STR) typing (service by DSMZ, results in Supplementary Tables), and cultivated in DMEM supplemented with 10% FCS, 100 U/ml penicillin and 100 µg/ml streptomycin (all from Thermo Scientific, Schwerte, Germany) at 37 °C and 7.5% CO_2_ as described^86^. Real-time functional assessment of bioenergetic pathways of cell lines was performed using a Seahorse Bioanalyzer (details in Supplementary Figure Fuel Flex Test). Total RNA was extracted from exponentially growing cultures using NucleoSpin RNA II Kit (Macherey & Nagel, Dueren, Germany). All animal experiments were performed according to the FELASA guidelines and upon ethical approval by the *Regierung von Oberbayern*. Athymic NU/NU (Crl:NU-Foxn1nu) mice were purchased from Charles River Laboratories (Sulzfeld, Germany) and housed in individually ventilated cages (GM500, Tecniplast, Hohenpeissenberg, Germany) within a specified, pathogen-free animal facility and 12 h day/night cycle. NU/NU mice feed (Ssniff, Soest, Germany) and water were provided ad libitum. Animals were inspected daily.

For orthotopic inoculation of HNSCC tumors, exponentially growing Cal33 and UPCI-SCC131 cells were harvested by TripLE Express (Thermo Scientific) treatment, washed twice in PBS, and adjusted to 1 × 10^8^ cells/ml in PBS. 10 µl of the cell suspension were mixed 1 + 1 with growth factor-reduced matrigel (Merck KGaA, Darmstadt, Germany). Mice were analgized by intraperitoneal injection of 0.1 µg/g buprenorphin (Bayer, Leverkusen, Germany) and anesthetized in 2-4% isoflurane dissolved in 0.8 l/min oxygen. When reaching surgical tolerance, a 2 × 2 mm^2^ Y-flap incision was made on the ventral side of the neck, and cells were injected in the region of the mylohyoid muscle. Skins were sealed with Ethibond Excel 5.0 suture thread (Johnson&Johnson GmbH, Norderstedt, Germany), and anesthesia was discontinued. Tumor growth was monitored by weekly contrast-enhanced conebeam computed tomography (CBCT) scans as described^87^, starting from d7 after inoculation. Upon reaching tumor volumes of ca. 100 mm^3^, animals were sacrificed by cardiac perfusion and tumors were explanted. 3’ RNAseq data of HNSCC cell lines and orthotopic xenograft data were generated and processed according to the workflow of the patient cohort data (LMU-KKG 13-16). Additionally, reads were aligned both to the human and mouse (*Mus musculus* GRCm38 reference) genomes. GSVA with KEGG metabolic pathways, MSigDB hallmarks, and gene signatures was performed analogously as for the clinical patient cohorts. MPS classification of samples was carried out by NSC classification with models trained on the LMU-KKG 13-16 and TCGA data sets. Mouse genome-aligned gene expression data were used for the deconvolution of the host (mouse) TME (R package SSMD^88^, tissue type: “Inflammatory”). Recovered cell type marker genes are shown in Supplementary Table 16.

### MALDI-MSI data generation and analysis

MALDI mass spectrometry imaging measurements of fresh-frozen tumor tissue sections were performed as previously described^89^. Metabolite annotation was performed using the Human Metabolome Database (HMDB, http://www.hmdb.ca/)^90^ and METASPACE (https://metaspace2020.eu/)^91^.

### Reporting summary

Further information on research design is available in the Nature Research Reporting Summary linked to this article.

### Supplementary information

REPORTING SUMMARY

Supplementary Information

Supplementary Data

## Data Availability

Bulk RNA sequencing data of the LMU-KKG cohort used in this study have been deposited at GEO under GSE205308, and GSE235223. Gene and protein expression data of the CPTAC-HNSCC cohort are publicly available (www.linkedomics.org/data_download/CPTAC-HNSCC/). RNA sequencing data of the TCGA cohort (harmonized collection, accessed on 11.07.2022) were obtained through the TCGAbiolinks R library. Transcriptomic data of the LHSC OPC cohort are confidential and were obtained through personal communication and permission by A. Nichols. Processed scRNAseq data of the Puram et al. and raw scRNAseq data of the Kürten et al. data sets were obtained from GEO under GSE103322, and GSE164690, respectively. Microarray gene expression data sets of the two HNSCC cohorts are available from GEO (GSE65858 and GSE41613).

## Code availability

All code for data preprocessing and analysis associated with the current submission is available upon request. The standard pipeline used for MPS subtyping of tumors was implemented as an R library MetabolicExpressR and is available at https://github.com/dBenedek/MetabolicExpressR.

## References

[1] Johnson DE (2020). Head and neck squamous cell carcinoma. Nat. Rev. Dis. Prim..

[2] Lawrence MS (2015). Comprehensive genomic characterization of head and neck squamous cell carcinomas. Nature.

[3] Chen SMY (2020). Tumor immune microenvironment in head and neck cancers. Mol. Carcinog..

[4] Schrank TP (2023). Noncanonical HPV carcinogenesis drives radiosensitization of head and neck tumors. Proc. Natl Acad. Sci..

[5] Peltanova B, Raudenska M, Masarik M (2019). Effect of tumor microenvironment on pathogenesis of the head and neck squamous cell carcinoma: a systematic review. Mol. Cancer.

[6] Patel U (2020). Prognostic and predictive significance of nuclear HIF1α expression in locally advanced HNSCC patients treated with chemoradiation with or without nimotuzumab. Br. J. Cancer.

[7] Sørensen BS, Horsman MR (2020). Tumor Hypoxia: Impact on radiation therapy and molecular pathways. Front. Oncol..

[8] Gong Y (2021). Metabolic-pathway-based subtyping of triple-negative breast cancer reveals potential therapeutic targets. Cell Metab..

[9] Weber P (2022). Therapy-related transcriptional subtypes in matched primary and recurrent head and neck cancer. Clin. Cancer Res. J. Am. Assoc. Cancer Res..

[10] Keck MK (2015). Integrative analysis of head and neck cancer identifies two biologically distinct HPV and three Non-HPV subtypes. Clin. Cancer Res..

[11] Schrank TP (2021). Genomic heterogeneity and copy number variant burden are associated with poor recurrence-free survival and 11q loss in human papillomavirus-positive squamous cell carcinoma of the oropharynx. Cancer.

[12] Puram SV (2023). Cellular states are coupled to genomic and viral heterogeneity in HPV-related oropharyngeal carcinoma. Nat. Genet..

[13] Huang C (2021). Proteogenomic insights into the biology and treatment of HPV-negative head and neck squamous cell carcinoma. Cancer Cell.

[14] Bartman CR (2023). Slow TCA flux and ATP production in primary solid tumours but not metastases. Nature.

[15] Sun N (2021). Native glycan fragments detected by MALDI mass spectrometry imaging are independent prognostic factors in pancreatic ductal adenocarcinoma. EJNMMI Res.

[16] Wei J, Hu M, Huang K, Lin S, Du H (2020). Roles of Proteoglycans and Glycosaminoglycans in cancer development and progression. Int. J. Mol. Sci..

[17] Pudełko A, Wisowski G, Olczyk K, Koźma EM (2019). The dual role of the glycosaminoglycan chondroitin‐6‐sulfate in the development, progression and metastasis of cancer. FEBS J..

[18] Puram SV (2017). Single-cell transcriptomic analysis of primary and metastatic tumor ecosystems in head and neck cancer. Cell.

[19] Morgan EL (2023). Inhibition of USP14 promotes TNFα-induced cell death in head and neck squamous cell carcinoma (HNSCC). Cell Death Differ..

[20] Matzinger O (2015). The radiosensitizing activity of the SMAC-mimetic, Debio 1143, is TNFα-mediated in head and neck squamous cell carcinoma. Radiother. Oncol..

[21] Derakhshan A, Chen Z, Van Waes C (2017). Therapeutic small molecules target inhibitor of apoptosis proteins in cancers with deregulation of extrinsic and intrinsic cell death pathways. Clin. Cancer Res..

[22] Reimand J (2019). Pathway enrichment analysis and visualization of omics data using g:Profiler, GSEA, Cytoscape and EnrichmentMap. Nat. Protoc..

[23] Chakravarthy A (2016). Human Papillomavirus drives tumor development throughout the head and neck: improved prognosis is associated with an immune response largely restricted to the Oropharynx. J. Clin. Oncol..

[24] Zeng PYF (2022). Immune-based classification of HPV-associated oropharyngeal cancer with implications for biomarker-driven treatment de-intensification. eBioMedicine.

[25] Kürten CHL (2021). Investigating immune and non-immune cell interactions in head and neck tumors by single-cell RNA sequencing. Nat. Commun..

[26] Bankhead P (2017). QuPath: Open source software for digital pathology image analysis. Sci. Rep..

[27] Gaude E, Frezza C (2016). Tissue-specific and convergent metabolic transformation of cancer correlates with metastatic potential and patient survival. Nat. Commun..

[28] Gentric G (2019). PML-regulated mitochondrial metabolism enhances chemosensitivity in human ovarian cancers. Cell Metab..

[29] O’Sullivan B (2016). Development and validation of a staging system for HPV-related oropharyngeal cancer by the International Collaboration on Oropharyngeal cancer Network for Staging (ICON-S): a multicentre cohort study. Lancet Oncol..

[30] Berdiaki A (2021). Glycosaminoglycans: Carriers and targets for tailored anti-cancer therapy. Biomolecules.

[31] Wu Q (2022). Remodeling Chondroitin-6-Sulfate–mediated immune exclusion enhances anti–PD-1 response in colorectal cancer with microsatellite stability. Cancer Immunol. Res..

[32] Thelin M (2012). Dermatan sulfate is involved in the tumorigenic properties of Esophagus Squamous Cell Carcinoma. Cancer Res.

[33] Chakravarthy A, Khan L, Bensler NP, Bose P, De Carvalho DD (2018). TGF-β-associated extracellular matrix genes link cancer-associated fibroblasts to immune evasion and immunotherapy failure. Nat. Commun..

[34] Davis SJ (2010). Metastatic potential of cancer stem cells in head and neck squamous cell carcinoma. Arch. Otolaryngol. Neck Surg..

[35] Keir ME, Butte MJ, Freeman GJ, Sharpe AH (2008). PD-1 and its ligands in tolerance and immunity. Annu. Rev. Immunol..

[36] Galbo PM, Zang X, Zheng D (2021). Molecular features of cancer-associated fibroblast subtypes and their implication on cancer pathogenesis, prognosis, and immunotherapy resistance. Clin. Cancer Res..

[37] Peltanová B (2022). mRNA subtype of cancer-associated fibroblasts significantly affects key characteristics of head and neck cancer cells. Cancers.

[38] Choi J-H (2023). Single-cell transcriptome profiling of the stepwise progression of head and neck cancer. Nat. Commun..

[39] Ha SY, Yeo S-Y, Xuan Y, Kim S-H (2014). The prognostic significance of cancer-associated fibroblasts in esophageal squamous cell carcinoma. PLOS ONE.

[40] Harris AL (2020). Development of cancer metabolism as a therapeutic target: new pathways, patient studies, stratification and combination therapy. Br. J. Cancer.

[41] Haddad RI (2023). Nivolumab Plus Ipilimumab versus EXTREME regimen as first-line treatment for recurrent/metastatic squamous cell carcinoma of the head and neck: the final results of CheckMate 651. J. Clin. Oncol. J. Am. Soc. Clin. Oncol..

[42] Hess J (2019). A five-MicroRNA signature predicts survival and disease control of patients with head and neck cancer negative for HPV infection. Clin. Cancer Res..

[43] Edge SB, Compton CC (2010). The American Joint Committee on Cancer: the 7th Edition of the AJCC Cancer Staging Manual and the Future of TNM. Ann. Surg. Oncol..

[44] Wintergerst L (2018). A prognostic mRNA expression signature of four 16q24.3 genes in radio(chemo)therapy-treated head and neck squamous cell carcinoma (HNSCC). Mol. Oncol..

[45] Kanehisa M, Goto S (2000). KEGG: Kyoto Encyclopedia of Genes and Genomes. Nucleic Acids Res..

[46] Kanehisa M, Furumichi M, Sato Y, Ishiguro-Watanabe M, Tanabe M (2020). KEGG: integrating viruses and cellular organisms. Nucleic Acids Res..

[47] Dobin A (2013). STAR: ultrafast universal RNA-seq aligner. Bioinformatics.

[48] Anders S, Pyl PT, Huber W (2015). HTSeq—a Python framework to work with high-throughput sequencing data. Bioinformatics.

[49] Love MI, Huber W, Anders S (2014). Moderated estimation of fold change and dispersion for RNA-seq data with DESeq2. Genome Biol..

[50] Colaprico A (2016). TCGAbiolinks: an R/Bioconductor package for integrative analysis of TCGA data. Nucleic Acids Res..

[51] Silva, T. C. et al. *TCGA Workflow*: Analyze cancer genomics and epigenomics data using Bioconductor packages. Preprint at 10.12688/f1000research.8923.2 (2016). 10.12688/f1000research.8923.2 PMC5302158 28232861

[52] Mounir M (2019). New functionalities in the TCGAbiolinks package for the study and integration of cancer data from GDC and GTEx. PLOS Comput. Biol..

[53] Wichmann G (2015). The role of HPV RNA transcription, immune response-related gene expression and disruptive TP53 mutations in diagnostic and prognostic profiling of head and neck cancer. Int. J. Cancer.

[54] Lohavanichbutr P (2013). A 13-gene signature prognostic of HPV-negative OSCC: Discovery and external validation. Clin. Cancer Res..

[55] Ritchie ME (2015). limma powers differential expression analyses for RNA-sequencing and microarray studies. Nucleic Acids Res..

[56] Stuart T (2019). Comprehensive Integration of Single-cell data. Cell.

[57] Tickle, T., Tirosh, I., Georgescu, C., Brown M. & Haas, B. inferCNV of the Trinity CTAT Project. https://github.com/broadinstitute/inferCNV (2019).

[58] Bates D, Mächler M, Bolker B, Walker S (2015). Fitting linear mixed-effects models using lme4. J. Stat. Softw..

[59] Kuznetsova A, Brockhoff PB, Christensen RH (2017). B. lmerTest Package: Tests in linear mixed effects models. J. Stat. Softw..

[60] Cillo AR (2020). Immune landscape of viral- and carcinogen-driven head and neck cancer. Immunity.

[61] Hänzelmann S, Castelo R, Guinney J (2013). GSVA: gene set variation analysis for microarray and RNA-seq data. BMC Bioinforma..

[62] Ayers M (2017). IFN-**γ**–related mRNA profile predicts clinical response to PD-1 blockade. J. Clin. Invest..

[63] Wu, G. & Haw, R. Functional Interaction Network Construction and Analysis for Disease Discovery. in *Protein Bioinformatics: From Protein Modifications and Networks to Proteomics* (eds. Wu, C. H., Arighi, C. N. & Ross, K. E.) 235–253 (Springer, New York, NY, 2017). 10.1007/978-1-4939-6783-4_11. 10.1007/978-1-4939-6783-4_11 28150241

[64] Fife BT, Bluestone JA (2008). Control of peripheral T-cell tolerance and autoimmunity via the CTLA-4 and PD-1 pathways. Immunol. Rev..

[65] Wu Z-H, Li C, Zhang Y-J, Zhou W (2022). Identification of a cancer stem cells signature of head and neck squamous cell carcinoma. Front. Genet..

[66] Schubert M (2018). Perturbation-response genes reveal signaling footprints in cancer gene expression. Nat. Commun..

[67] Charrad M, Ghazzali N, Boiteau V, Niknafs A (2014). NbClust: An R Package for determining the relevant number of clusters in a data set. J. Stat. Softw..

[68] R Core Team. R: A Language and Environment for Statistical Computing. https://www.R-project.org/ (2019).

[69] Tibshirani R, Hastie T, Narasimhan B, Chu G (2002). Diagnosis of multiple cancer types by shrunken centroids of gene expression. Proc. Natl Acad. Sci..

[70] Hastie, T., Tibshirani, R. Balasubramanian Narasimhan, & Gil Chu. pamr: Pam: Prediction Analysis for Microarrays. https://CRAN.R-project.org/package=pamr (2019).

[71] Terry T. M. A Package for Survival Analysis in R. https://CRAN.R-project.org/package=survival (2022).

[72] Terry T. M. & Patricia M. Grambsch. *Modeling Survival**Data: Extending the Cox Model*. (Springer, New York, 2000).

[73] Kassambara, A., Kosinski, M. & Biecek, P. survminer: Drawing Survival Curves using ‘ggplot2’. https://CRAN.R-project.org/package=survminer (2019).

[74] Payton ME, Greenstone MH, Schenker N (2003). Overlapping confidence intervals or standard error intervals: What do they mean in terms of statistical significance?. J. Insect Sci..

[75] Kuhn, M. caret: Classification and Regression Training. Astrophysics Source Code Library ascl:1505.003 (2015).

[76] Venables, W. N. & Ripley, B. D. *Modern Applied Statistics with S*. (Springer, New York, NY, 2002). 10.1007/978-0-387-21706-2.

[77] Haibe-Kains B, Desmedt C, Sotiriou C, Bontempi G (2008). A comparative study of survival models for breast cancer prognostication based on microarray data: does a single gene beat them all?. Bioinformatics.

[78] Schröder MS, Culhane AC, Quackenbush J, Haibe-Kains B (2011). survcomp: an R/Bioconductor package for performance assessment and comparison of survival models. Bioinformatics.

[79] Subramanian A (2005). Gene set enrichment analysis: A knowledge-based approach for interpreting genome-wide expression profiles. Proc. Natl Acad. Sci..

[80] Liberzon A (2011). Molecular signatures database (MSigDB) 3.0. Bioinformatics.

[81] Liberzon A (2015). The Molecular Signatures Database Hallmark Gene Set Collection. Cell Syst..

[82] Cohen, J. *Statistical Power Analysis for the Behavioral Sciences*. (New York: Academic Press, New York, NY, 1988).

[83] Korotkevich, G. et al. Fast gene set enrichment analysis. 060012 Preprint at 10.1101/060012 (2021).

[84] Marzouka N-A-D, Eriksson P (2021). multiclassPairs: an R package to train multiclass pair-based classifier. Bioinformatics.

[85] Newman AM (2019). Determining cell type abundance and expression from bulk tissues with digital cytometry. Nat. Biotechnol..

[86] Schoetz U (2021). Early senescence and production of senescence-associated cytokines are major determinants of radioresistance in head-and-neck squamous cell carcinoma. Cell Death Dis..

[87] Stegen B (2020). Contrast-enhanced, conebeam CT-based, fractionated radiotherapy and follow-up monitoring of orthotopic mouse glioblastoma: a proof-of-concept study. Radiat. Oncol..

[88] Lu, X. et al. SSMD: a semi-supervised approach for a robust cell type identification and deconvolution of mouse transcriptomics data. *Brief. Bioinform*. **22**, bbaa307 (2021). 10.1093/bib/bbaa307 PMC8294548 33230549

[89] Murakami M (2023). In situ metabolomics of cortisol-producing adenomas. Clin. Chem..

[90] Wishart DS (2007). HMDB: the human metabolome database. Nucleic Acids Res.

[91] Palmer A (2017). FDR-controlled metabolite annotation for high-resolution imaging mass spectrometry. Nat. Methods.

