# Supplementary material for: Metabolic pathway-based subtypes associate glycan biosynthesis and treatment response in head and neck cancer
Source: NPJ Precis Oncol. 2024 May 23;8:116. doi: 10.1038/s41698-024-00602-0 (PMC11116554; doi:10.1038/s41698-024-00602-0)
Supplement: Supplementary file 2 — Supplementary Information [file 41698_2024_602_MOESM2_ESM.pdf]

Dankó et al., Metabolic pathway-based subtypes associate glycan biosynthesis and treatment response in head and neck cancer

Supplementary Information

Supplementary Figure 1: Deregulated metabolic gene and protein expression in HNSCC.

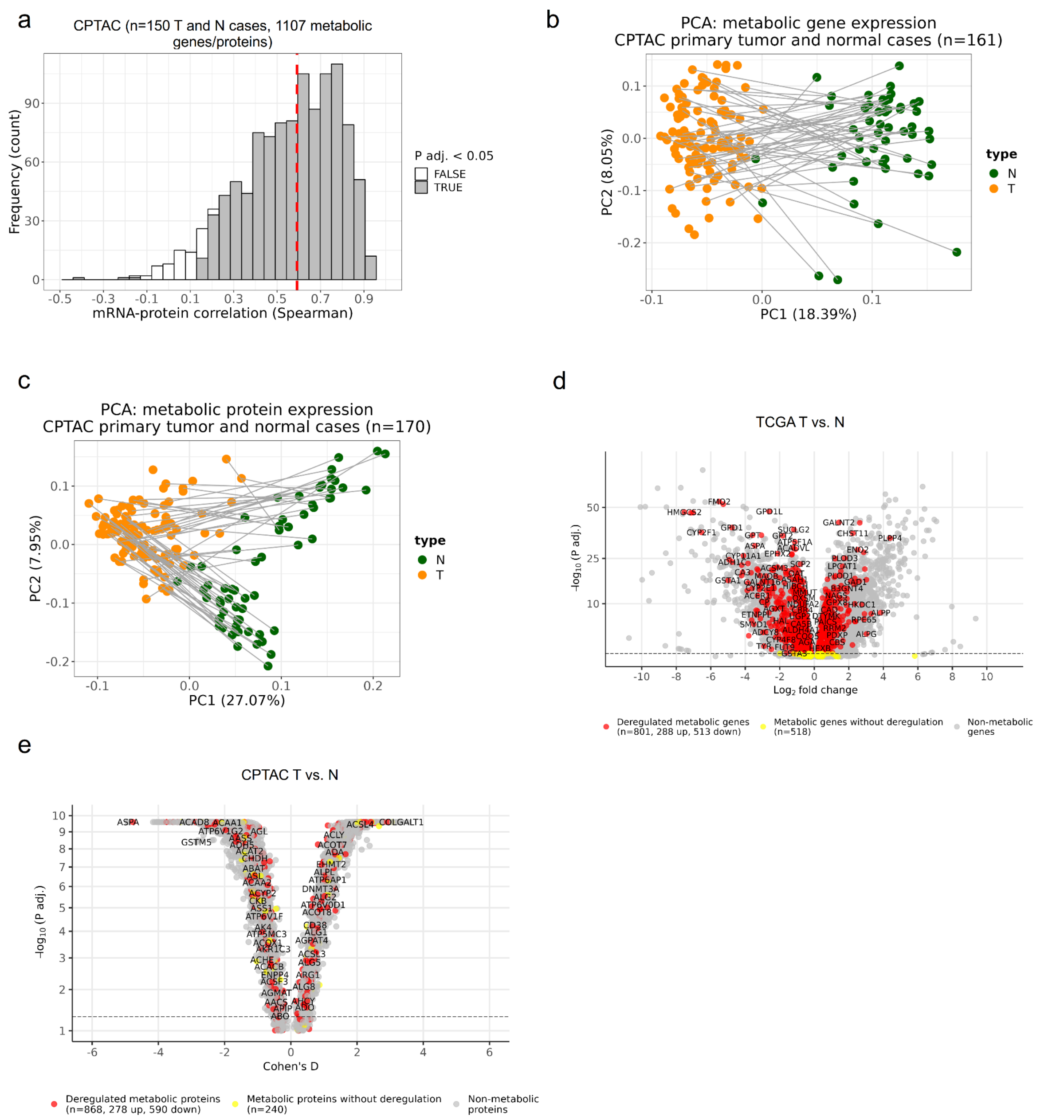

Histogram of correlation analysis of protein and gene expression data of 1107 metabolic genes/proteins from the KEGG metabolic pathways collection in the CPTAC-HNSCC cohort (150 tumor and normal cases) (**a**). Dashed line: median Spearman correlation coefficient (r=0.592). Principal component analysis (PCA) of all primary tumor and normal cases metabolic gene (n=161) and protein (n=170) expression data from the CPTAC cohort (**b, c**). Volcano plot representing deregulated metabolic gene expression between TCGA tumors and matched normal cases (n=74 pairs) (**d**). Volcano plot representing deregulated metabolic protein expression between CPTAC tumors and matched normal cases (n=62 pairs) (**e**). T: tumor, N: normal.

Supplementary Figure 2: Consistency and tumor cell specificity of the MPS.

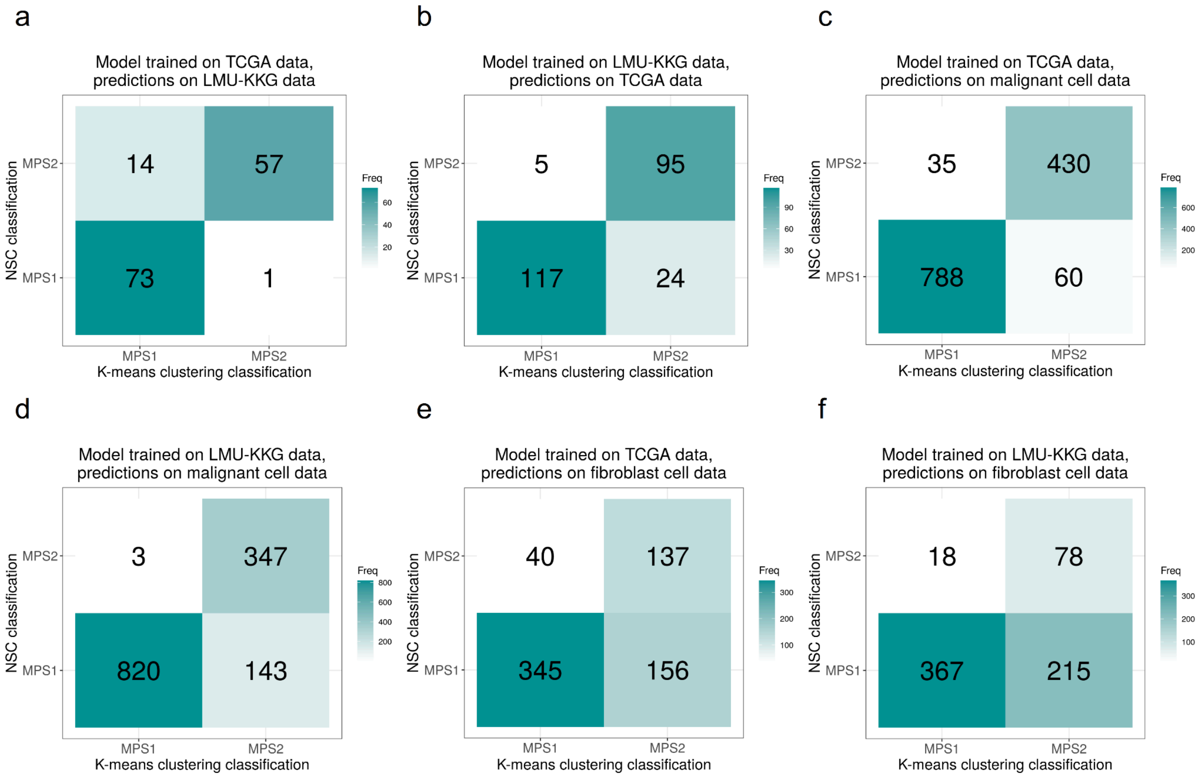

NSC classifier[1] was trained on the TCGA HPV-negative cohort (n=241) KEGG metabolic GSVA data and the MPS class was predicted on the LMU-KKG HPV-negative cohort (n=145) KEGG metabolic GSVA data and compared to the actual, k-means clustering-based MPS class (visualized as confusion matrix) (**a**). Consistent classification rate=0.897. NSC classifier was trained on the LMU-KKG HPV-negative cohort KEGG metabolic GSVA data and the MPS class was predicted on the TCGA HPV-negative cohort KEGG metabolic GSVA data and compared to the actual, k-means clustering-based MPS class (**b**). Consistent classification rate=0.880. NSC classifiers were trained on the TCGA HPV-negative cohort KEGG metabolic GSVA data, and the MPS class was predicted on the Puram et al.[2] malignant cell (n=1313) and fibroblast cell (n=678) KEGG metabolic GSVA data and compared to the actual, k-means clustering-based MPS class (**c, e**). Consistent

classification rate=0.928, 0.711, respectively. NSC classifiers were trained on the LMU-KKG HPV-negative cohort KEGG metabolic GSVA data, and the MPS class was predicted on the Puram et al. malignant cell and fibroblast cell KEGG metabolic GSVA data and compared to the k-means clustering-based MPS class (**d, f**). Consistent classification rate=0.890, 0.653, respectively.

Supplementary Figure 3: Tumors show metabolic deregulation both on the gene- and protein expression levels.

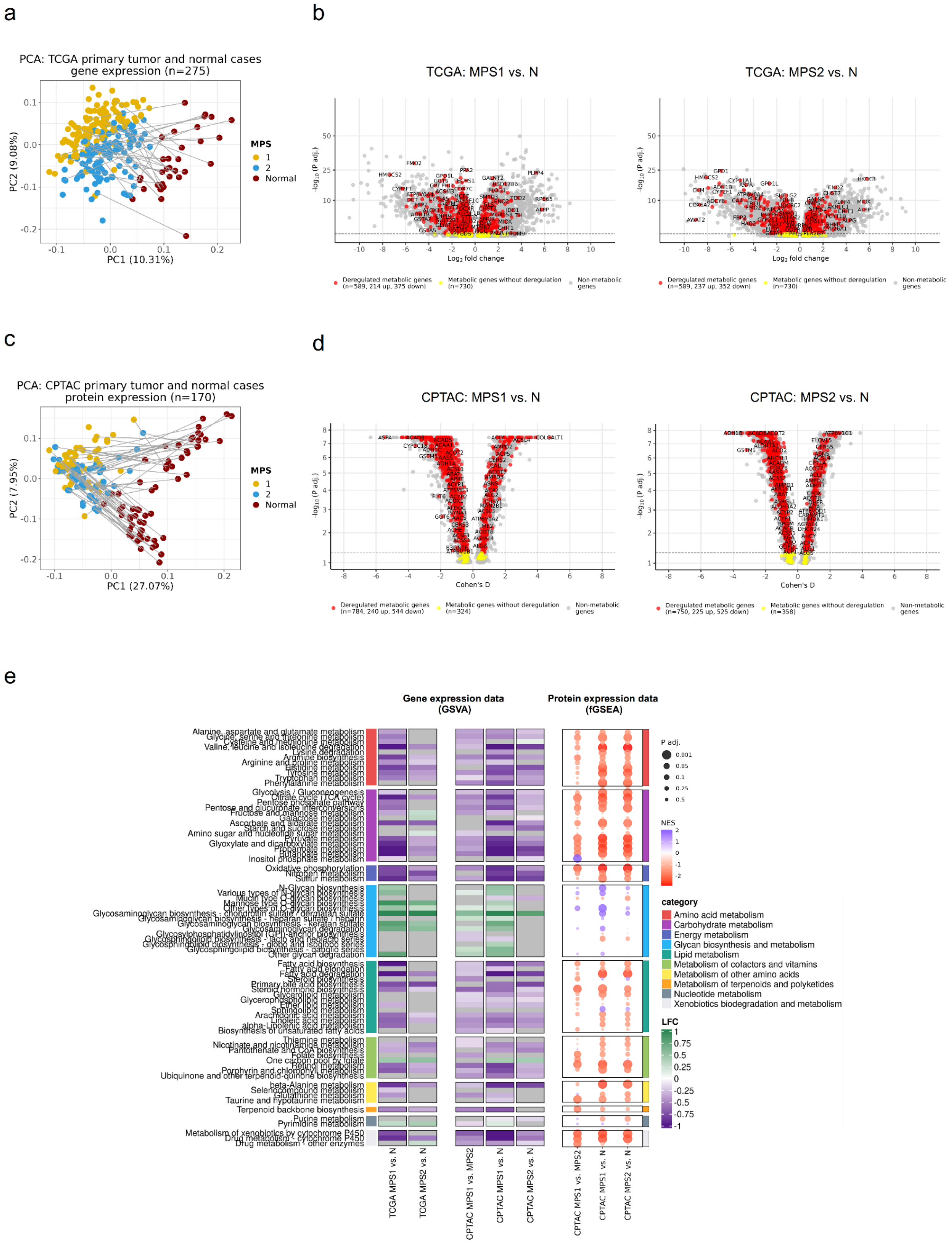

Principal component analysis (PCA) of metabolic gene expression data of the TCGA HPV-negative primary tumor and normal cases (n=275) (**a**). Gray lines connect tumor-normal pairs. The first two principal components are visualized. Volcano plots of the TCGA MPS1 vs. matched normal (n=16 pairs), and MPS2 vs. matched normal (n=18 pairs) differential gene expression analysis results, respectively (**b**). PCA of metabolic protein expression data of the CPTAC HPV-negative primary tumor and normal cases (n=170) (**c**). Volcano plots of the CPTAC MPS1 vs. matched normal (n=30 pairs), and MPS2 vs. matched normal (n=32 pairs) differential protein expression analysis results, respectively (**d**). LFC values of group comparisons of metabolic GSVA scores in TCGA and CPTAC HPV-negative cohorts. Non-significant (P adj.>0.05) pathways are indicated with gray color. On the right-hand side, fGSEA results of the corresponding group comparisons based on the CPTAC protein expression data are shown (**e**). NES: normalized enrichment score.

Supplementary Figure 4: MPS and further clinical endpoints in the HPV-negative LMU-KKG 13-16 cohort.

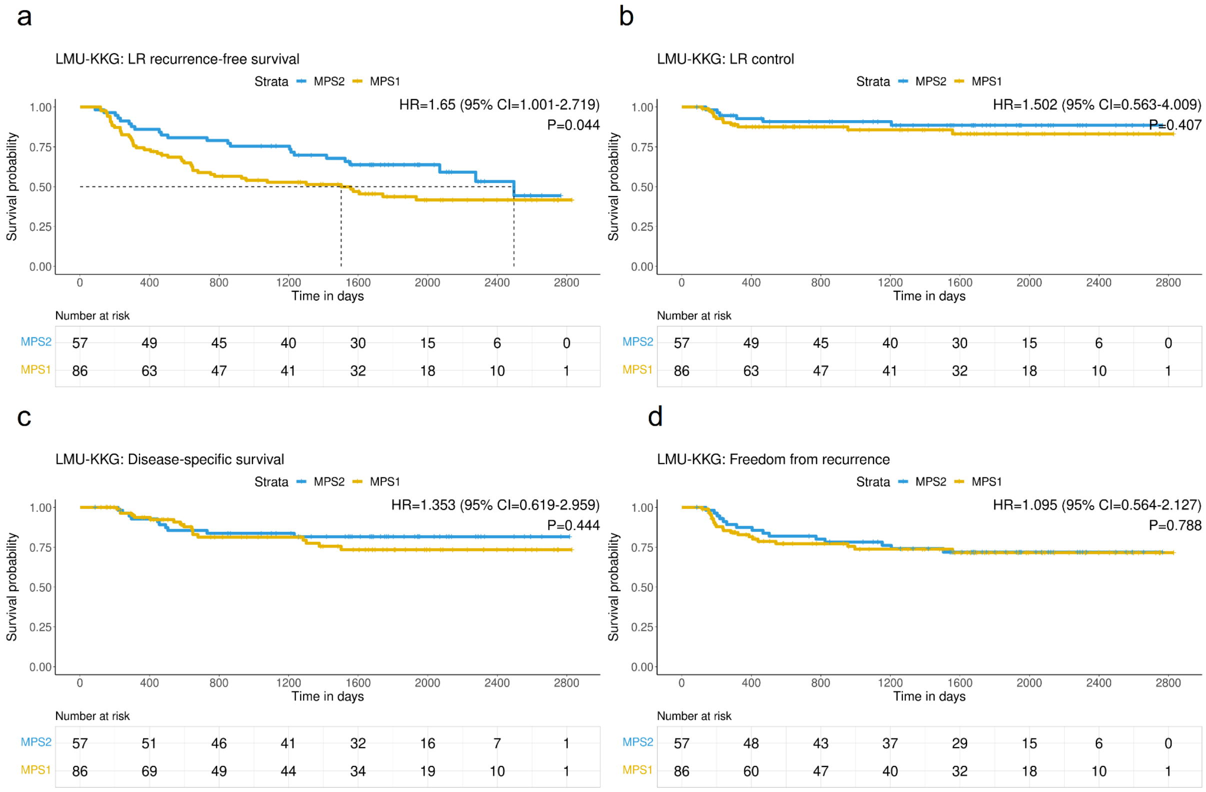

The univariable Kaplan-Meier (KM) plots of the LMU-KKG cohort HPV-negative subset for LR recurrence-free survival (**a**), LR control (**b**), disease-specific survival (**c**), and freedom from recurrence (**d**), respectively. HRs with 95% CI are shown.

Supplementary Figure 5: Keck subtypes and clinical endpoints in the LMU-KKG HPV-negative cohort.

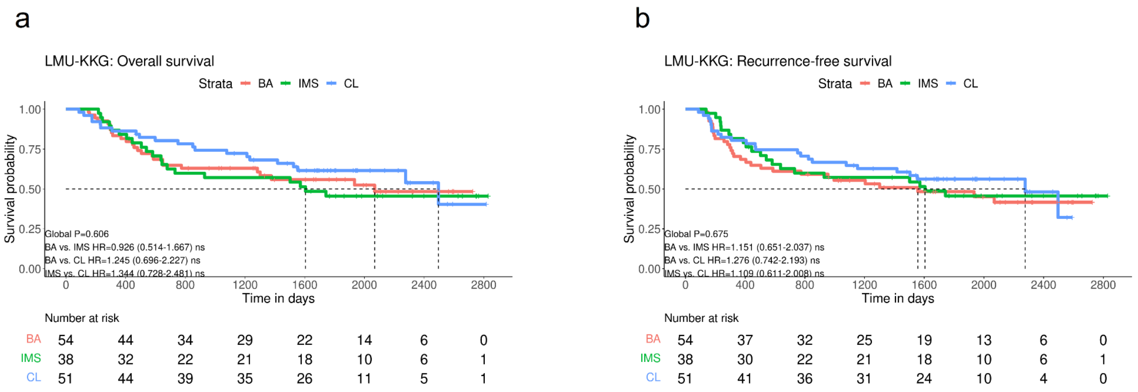

The univariable Kaplan-Meier (KM) plots of the LMU-KKG cohort HPV-negative subset with Keck et al. transcriptional subtypes for overall survival (OS) (**a**), and recurrence-free survival (RFS) (**b**). Pairwise HRs with 95% CI are indicated. ***: P-value < 0.001, **: P-value >= 0.001 and < 0.01, ***: P-value >= 0.01 and < 0.05, ns: P-value >= 0.05. Global P: global logrank P-value. BA: basal, IMS: inflamed/mesenchymal, CL: classical.

Supplementary Figure 6: Sub-stratification of known therapy response-associated clinical variables by MPS (overall survival).

a

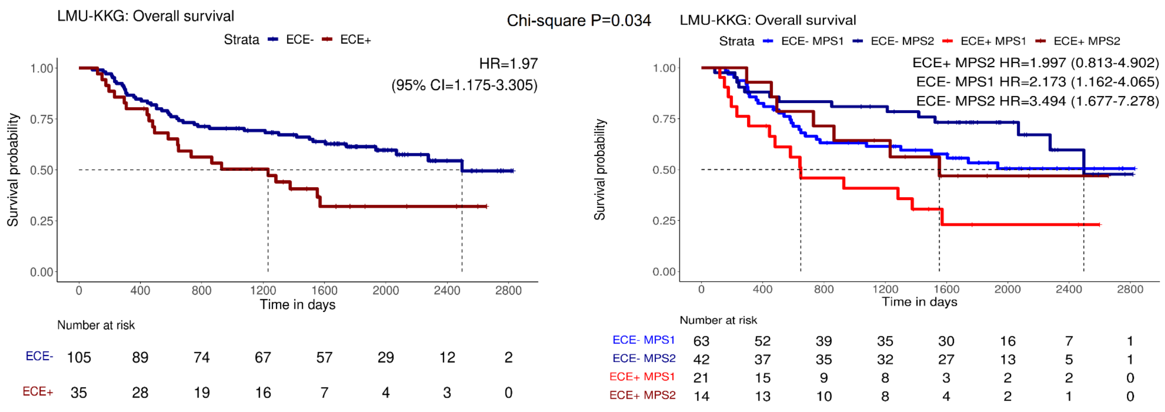

b

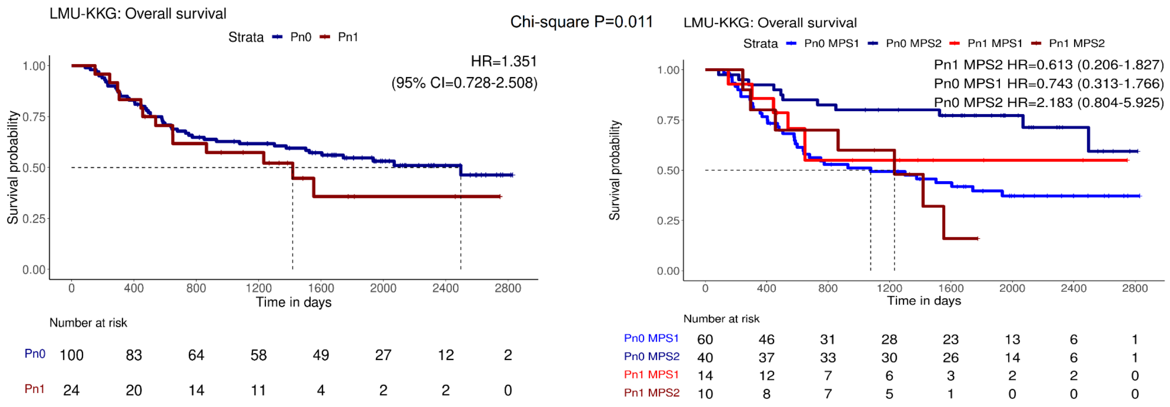

KM analysis was performed on the HPV-negative LMU-KKG cohort using therapy response-associated clinical variables alone or in combination with MPS. The endpoint of overall survival for ECE (**a**), and PNI (Pn) stage (**b**) are shown here (other clinical variables in Fig. 2). Chi-square test P-values of the two models' comparisons (two-group or four-group models) are indicated. In the case of the four-group models, subgroups are compared to the ECE+ MPS1 (a) or Pn1 MPS1 (b) groups, and pairwise HRs with 95% CI are indicated.

Supplementary Figure 7: Sub-stratification of known therapy response-associated clinical variables by MPS (recurrence-free survival).

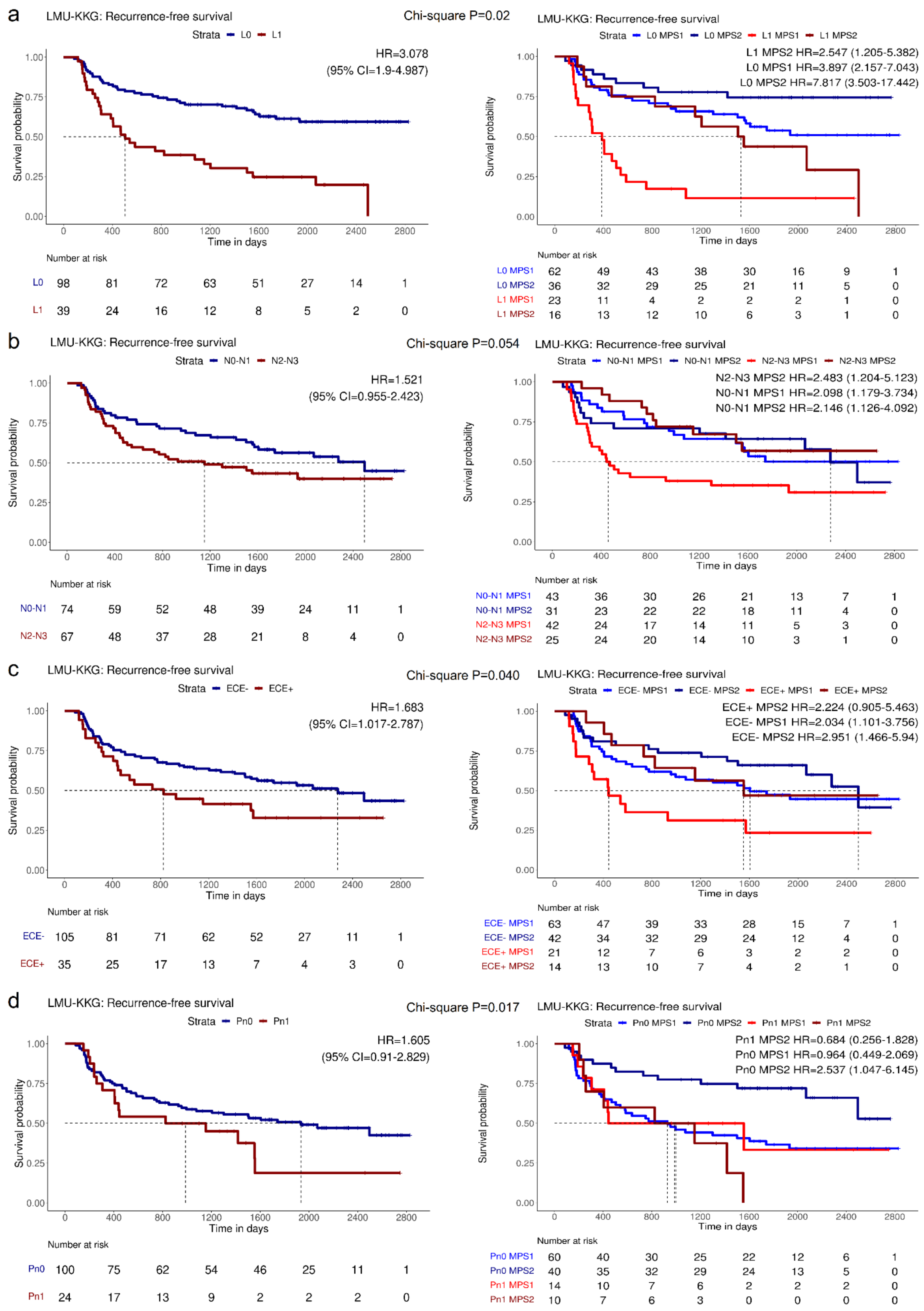

KM analysis was performed on the HPV-negative LMU-KKG cohort using therapy response-associated clinical variables alone or in combination with MPS. The endpoint of recurrence-free survival for LVI (L) stage (**a**), N stage (**b**), ECE (**c**), and PNI (Pn) stage (**d**) are shown here (other clinical variables in Fig. 2). Chi-square test P-values of the two models' comparisons (two-group or four-group models) are indicated. In the case of the four-group models, subgroups are compared to the L1 MPS1 (a), N2-3 MPS1 (b), ECE+ MPS1 (c), or Pn+ MPS1 (d) reference groups, and pairwise HRs with 95% CI are indicated.

Supplementary Figure 8: KEGG pathway map of the glycosaminoglycan biosynthesis – chondroitin/dermatan sulfate pathway and its differentially expressed genes between MPS1 and MPS2.

a

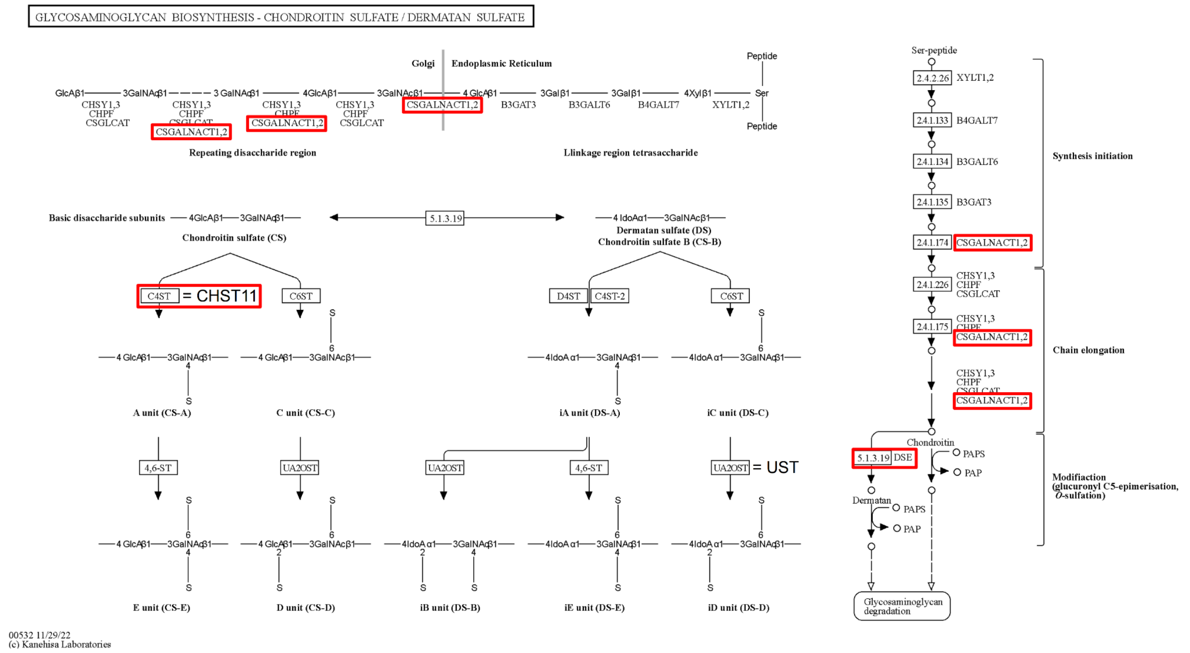

b

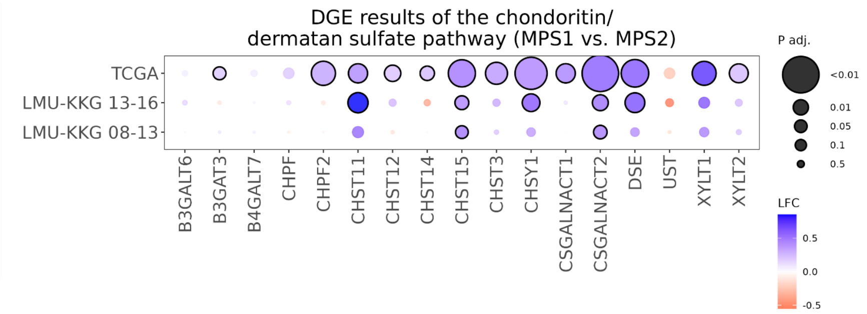

KEGG pathway map obtained from the KEGG website www.genome.jp/pathway/map00532 (accessed on 20.01.2023)[3,4] (**a**). The most prominently upregulated genes in MPS1 are highlighted by red rectangles. Differential gene expression analysis was carried out on the three data sets separately (HPV-negative cases) using DESeq2[5] (**b**). Benjamini-Hochberg P-value adjustment was applied and log2 fold-change (LFC) and adjusted P-values (P adj.) are visualized for the genes of the *glycosaminoglycan biosynthesis –*

chondroitin/dermatan sulfate KEGG metabolic pathway in a dot-plot. The genes *CHST11*, *DSE*, and *CSGALNACT2* (highlighted in **a**) are consistently upregulated in MPS1 compared to MPS2 in the three data sets (*CHST11*: P ajd.=0.065, <0.001, <0.001, LFC=0.547, 0.854, 0.440; *DSE*: P ajd.=0.199, <0.001, <0.001, LFC=0.405, 0.643, 0.610; *CSGALNACT2*: P ajd.=0.029, 0.006, <0.001, LFC=0.483, 0.534, 0.602 for the LMU-KKG 08-13, LMU-KKG 13-16, and TCGA data sets, respectively). Significant genes (P adj.<0.05) are highlighted with a black border. DGE: differential gene expression.

Supplementary Figure 9: CIBERSORTx analysis results with the Puram et al. reference matrix: relative cell fraction differences between MPS1 and MPS2 in the LMU-KKG 08-13, LMU-KKG 13-16, and TCGA HPV-negative data sets.

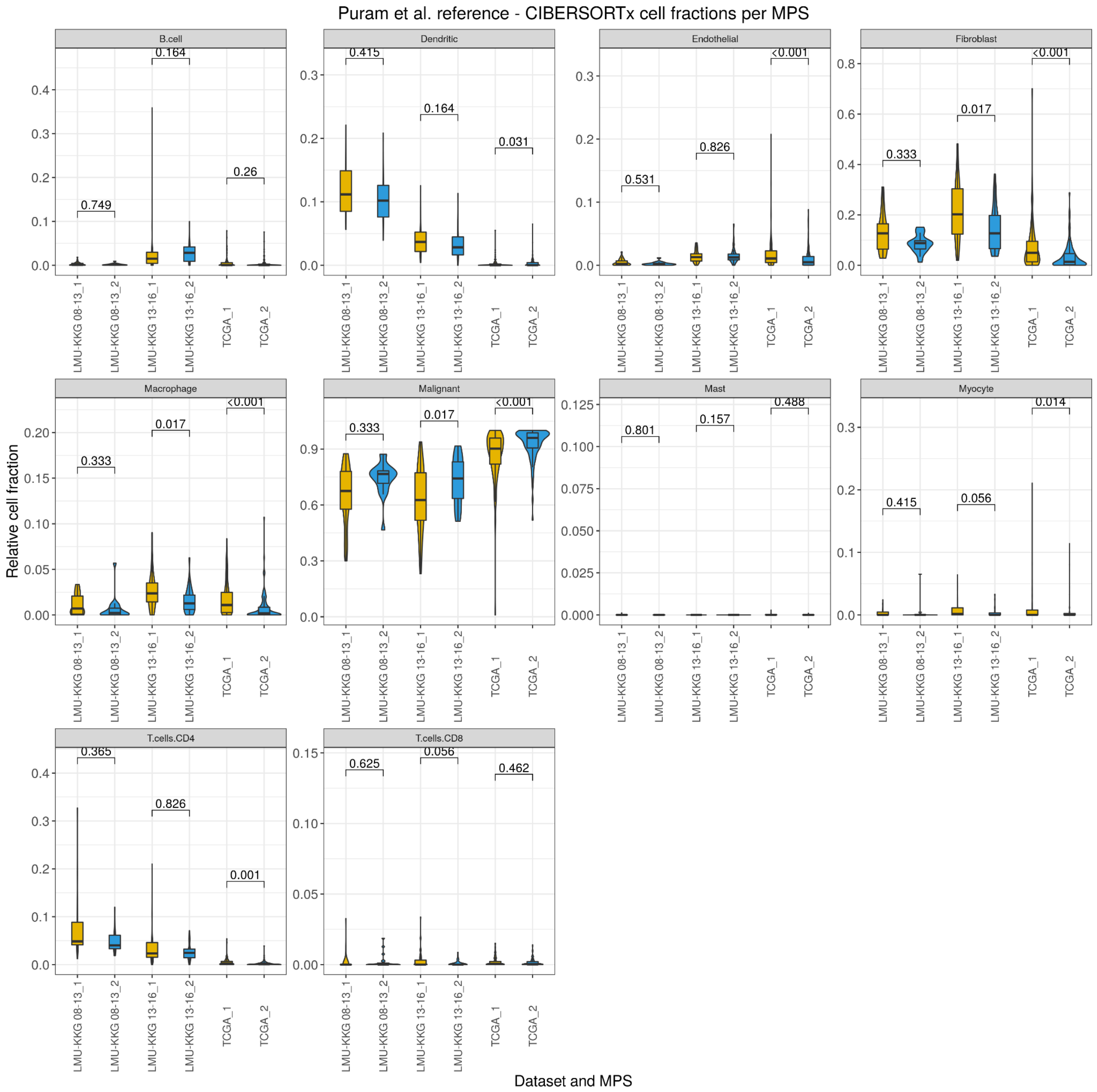

CIBERSORTx cell fractions mode was used for deconvolution on the HPV-negative cases of the LMU-KKG 08-13, LMU-KKG 13-16, and TCGA data sets with the Puram et al. reference matrix. Wilcoxon test was applied for the comparison of relative cell fraction between MPS1 and MPS2, and Benjamini-Hochberg P-value adjustment was done. MPS1 shows a consistently elevated level of dendritic cells, macrophages, fibroblasts, and myocytes, and a decreased level of malignant cells compared to MPS2.

Supplementary Figure 10: MPS in the TCGA full data set (n=500) of HPV-negative and HPV-positive tumors and non-canonical NFκB signaling in HPV-positive tumors.

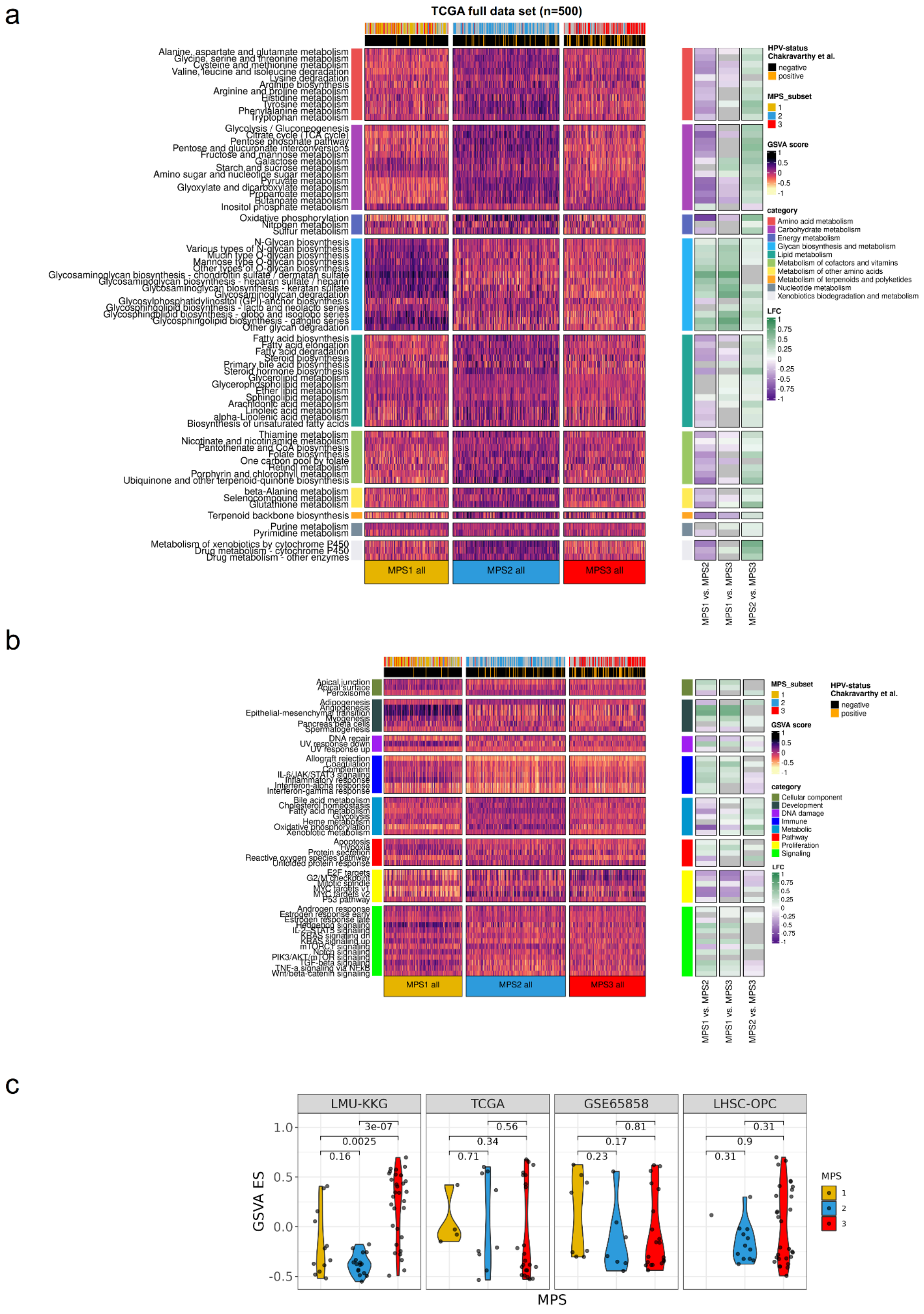

K-means clustering was performed with k=3 on the HPV-negative and HPV-positive cases of the TCGA cohort (n=500). HPV-status used here for the full cohort was determined by Chakravarthy et al.[6]. Heatmap of KEGG metabolic pathways GSVA

enrichment scores, according to the classes obtained by k-means clustering (k=3). On the right-hand side, LFC values of group comparisons of metabolic GSVA scores are shown. Frequencies of HVP-positive tumors in the respective MPS classes are, MPS1: n=5 HPV+ (3.4%), MPS2: n=24 HPV+ (14%), MPS3: n=39 HPV+ (35.2%) tumors. MPS3 was significantly enriched with HPV-pos. cases (Fisher's exact test P<0.001). (**a**). Non-significant (P adj.>0.05) pathways are indicated with grey color. Heatmap of MSigDB hallmarks GSVA enrichment scores with LFC values of group comparisons of metabolic GSVA scores are shown (**b**). Non-significant (P adj.>0.05) pathways are indicated with grey color. MPS_subset: MPS subtype defined on the reanalyzed (n=277) data set. Noncanonical NFκB signaling[7] was quantified in HPV-positive tumors of the cohorts LMU-KKG (n=59), TCGA (n=36), GSE65858 (n=35), and LHSC-OPC (n=43) using GSVA, and compared between MPS1, MPS2, and MPS3 (**c**).

Supplementary Figure 11: Classification of metabolic pathway-based subtypes in the HPV-positive LHSC OPC cohort.

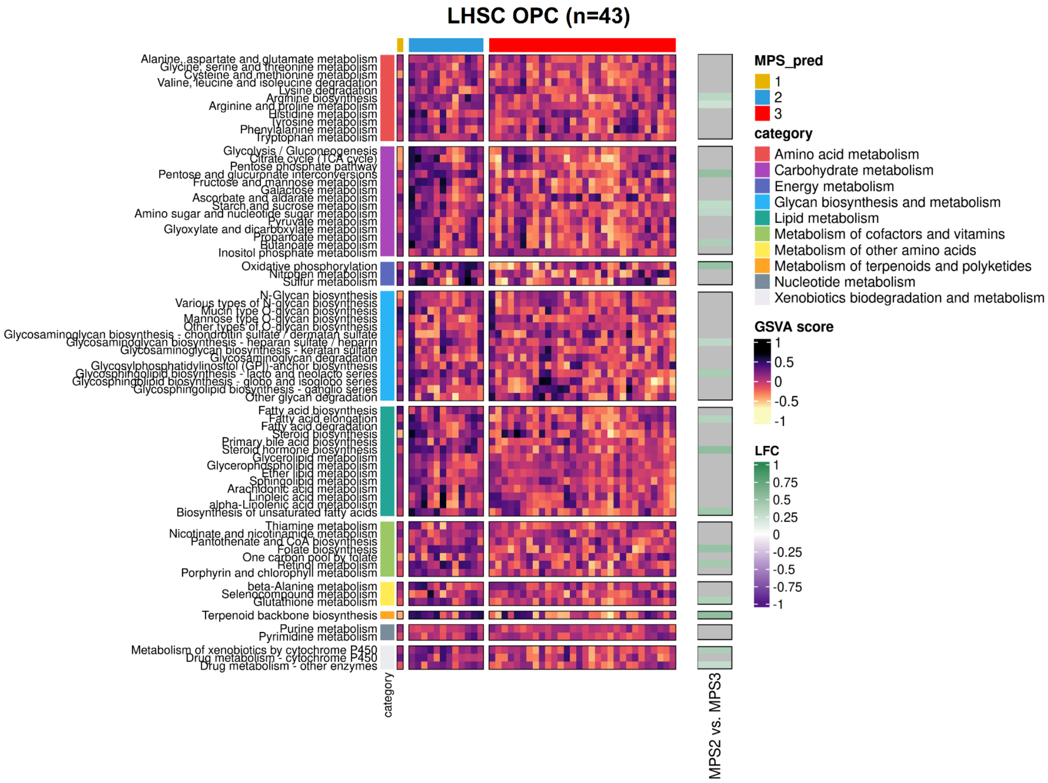

MPS classification into MPS1, MPS2, or MPS3 subtypes of the cases from the HPV-positive LHSC OPC cohort was conducted using a gene expression-based random forest (RF) classifier. The heatmap displays the GSVA enrichment scores of KEGG metabolic pathways for each tumor, organized by their respective MPS class. The heatmap on the right represents MPS2 vs. MPS3 log2 fold change (LFC) values of GSVA enrichment scores of the same metabolic pathways. Non-significant (P adj.>0.05) pathways are indicated with grey color. MPS_pred: gene expression-based predicted MPS class.

Supplementary Figure 12: Association of MPS and therapy outcome in univariable models presented on the HPV-negative and HPV-positive cases.

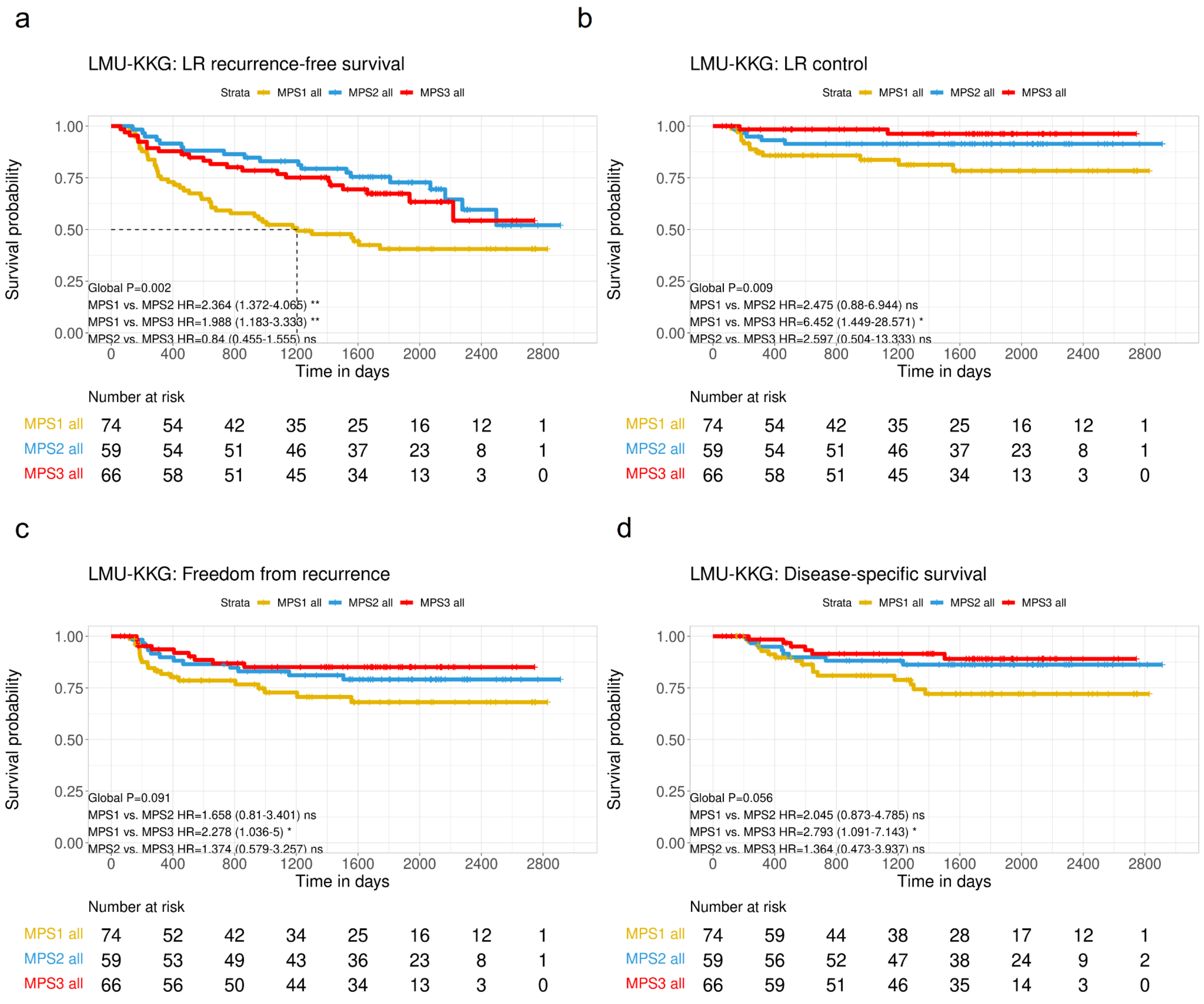

K-means clustering was performed with k=3 on the LMU-KKG HPV-negative and HPV-positive cases (n=204) in order to define the MPS class membership of the patients. Kaplan-Meier analysis was performed comparing MPS1, MPS2, and MPS3 for the endpoints of LR-RFS, LRC, FFR, and DSS (OS and RFS in Fig. 5). Pairwise HRs with 95% CI are indicated. ***: P-value < 0.001, **: P-value >= 0.001 and < 0.01, ***: P-value >= 0.01 and < 0.05, ns: P-value >= 0.05. Global P: logrank P-value.

Supplementary Figure 13: Kürten et al. KEGG metabolic GSVA scores of epithelial cell clusters and MPS1 malignant cell-specificity of CS/DS metabolism pathway.

a

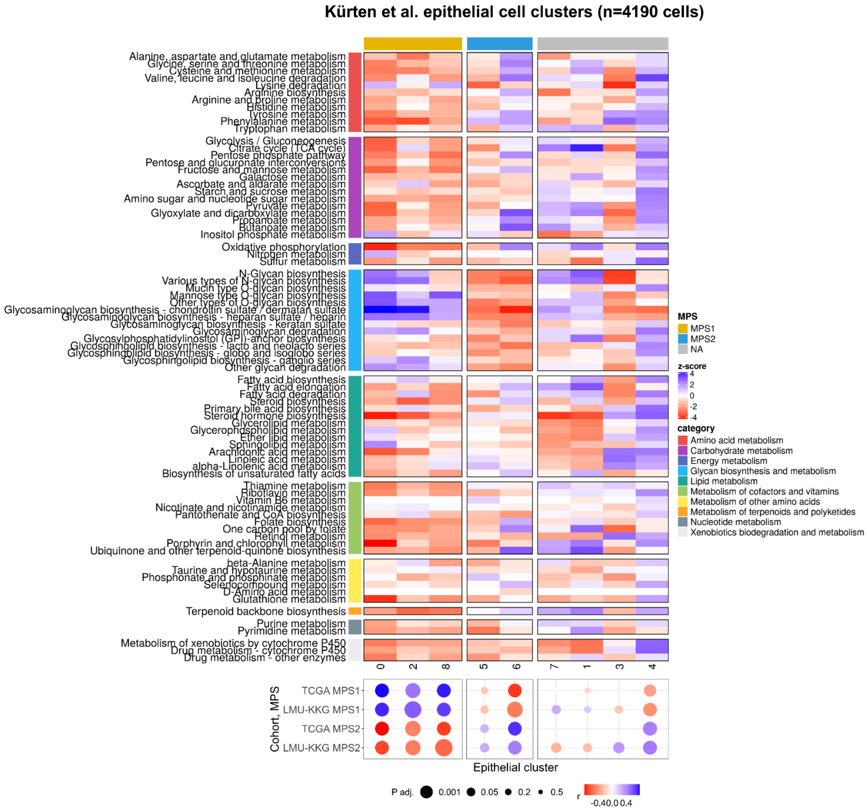

b

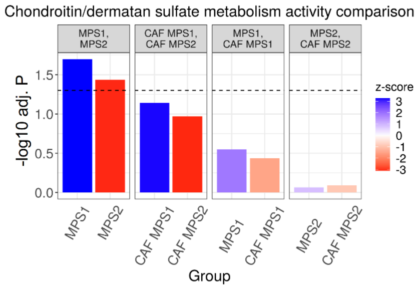

KEGG metabolic pathways enrichment scores of the Kürten et al. epithelial cell clusters identified by Seurat (**a**). The MPS class of clusters was defined based on correlation tests to the LMU-KKG and TCGA MPS centroids (see correlation plot). Significantly elevated CS/DS metabolism in MPS1 vs. MSP2 malignant cells (adj.P<0.05, pre-ranked GSEA), but not between CAFs from MPS1 and MPS2 tumors (**b**).

Supplementary Figure 14: Analysis of sliding threshold of IHC CSPG level and association with further clinical endpoints in the HPV-negative subset of the LMU-KKG cohort.

a

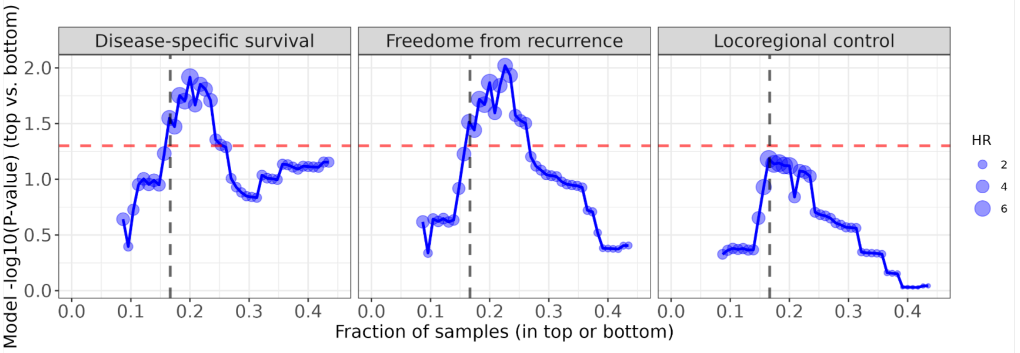

b

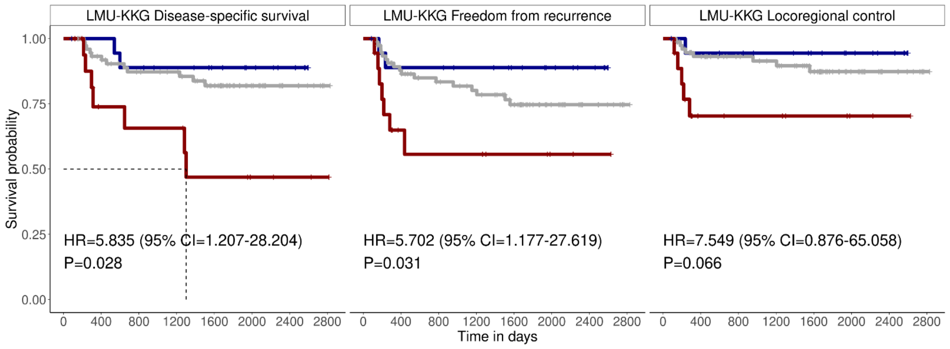

Samples from the LMU-KKG HPV-negative cohort (n=115) were grouped into top or bottom classes based on CSPG protein expression quantified by IHC. Using different symmetric thresholds (from 10% to 50% by 1%) for top and bottom grouping, Cox PH models were built and the two groups were compared with regards to HR (dot size) and model -log10(P-value) (y-axis) (**a**). The dashed red line indicates P-value=0.05 and the dashed black line shows the fraction of samples ⅙ (as in Fig. 6 **g** and **j**). Using the selected ⅙ threshold (which corresponds to ~17% on panel a), top and bottom groups based on CSPG expression were compared for the endpoints disease-specific survival, freedom from recurrence, and locoregional control (**b**).

Supplementary Figure 15: t-SNE of the Puram et al. and Kürten et al. scRNAseq data sets.

a

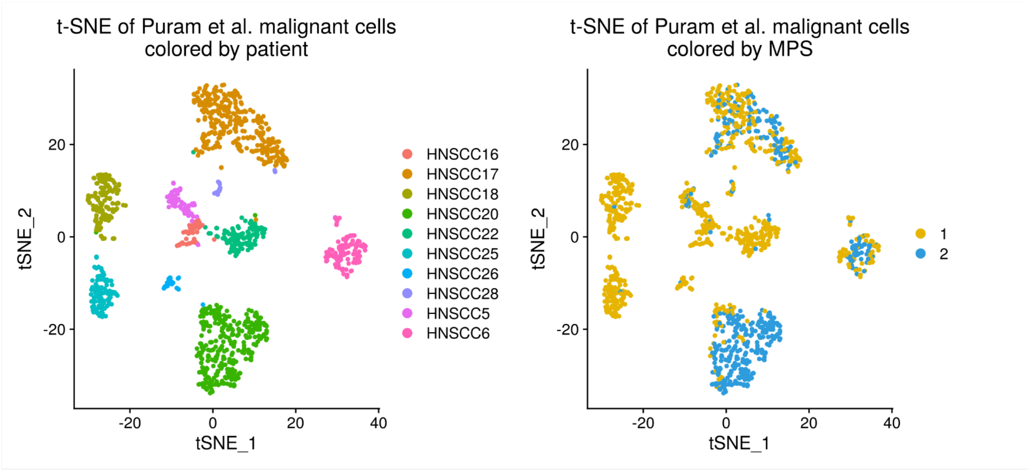

b

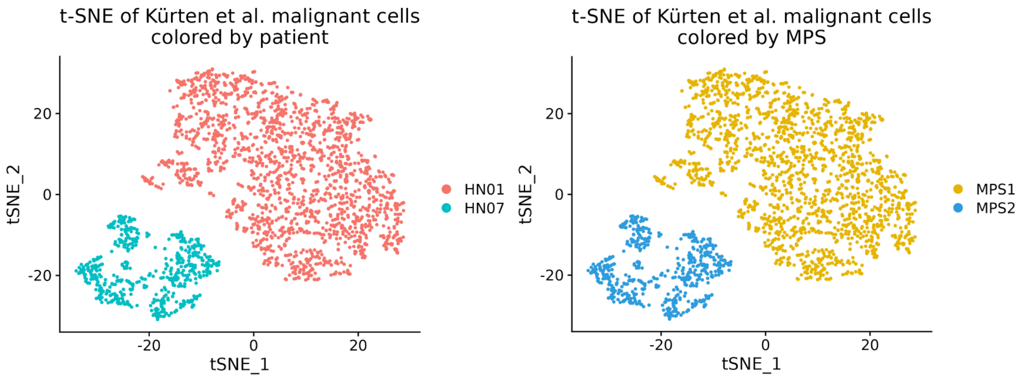

t-SNE of the Puram et al. malignant single-cell (n=1313) expression data of 10 HNSCC cases colored by patient and MPS, respectively (cell numbers and proportions in Supplementary Table 18) (**a-b**)**.** t-SNE of the MPS-classified malignant cells in the Kürten et al. data set, colored by patient and MPS, respectively (**c-d**). Only patients with more than 50 malignant cells and MPS class included (tumors HN01 and HN07, n=2400 cells).

Supplementary Figure 16: Further t-SNE plots of the Puram et al. and Kürten et al. scRNAseq data sets.

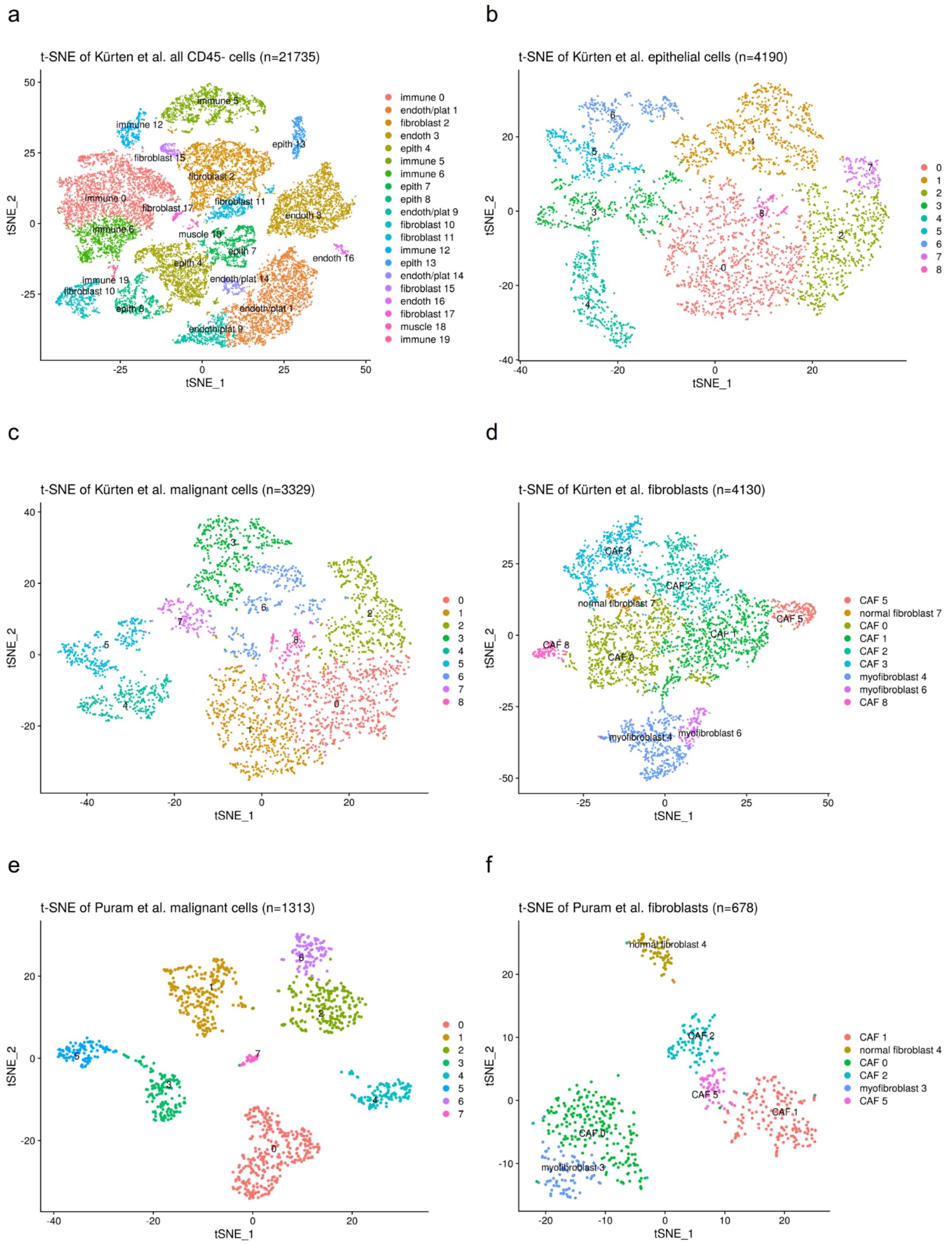

Identification of cell clusters was performed as outlined in Methods. t-SNE of all Kürten et al.[8] CD45-negative cell clusters (**a**). t-SNE of Kürten et al. epithelial cell clusters (**b**). Clusters 3, and 4: non-malignant cell clusters based on the InferCNV results (Supplementary Fig. 17). t-SNE of Kürten and Puram malignant cell clusters, respectively (**c, e**). Non-malignant epithelial cells were excluded based on the InferCNV results. t-SNE of Kürten and Puram fibroblast cell clusters, respectively (**d, f**). In the case of the Kürten et al. data set t-SNEs of epithelial/malignant, and fibroblast cells, sub-clustering was done on the selected cell subset after clustering and cell type annotation of all CD45-negative cells. The Puram et al. malignant and fibroblast cell subsets were already annotated by the authors. Only HPV-negative patients are included. CAF: cancer-associated fibroblast.

Supplementary Figure 17: InferCNV identification of malignant cell clusters from the Kürten et al. epithelial cell subsets.

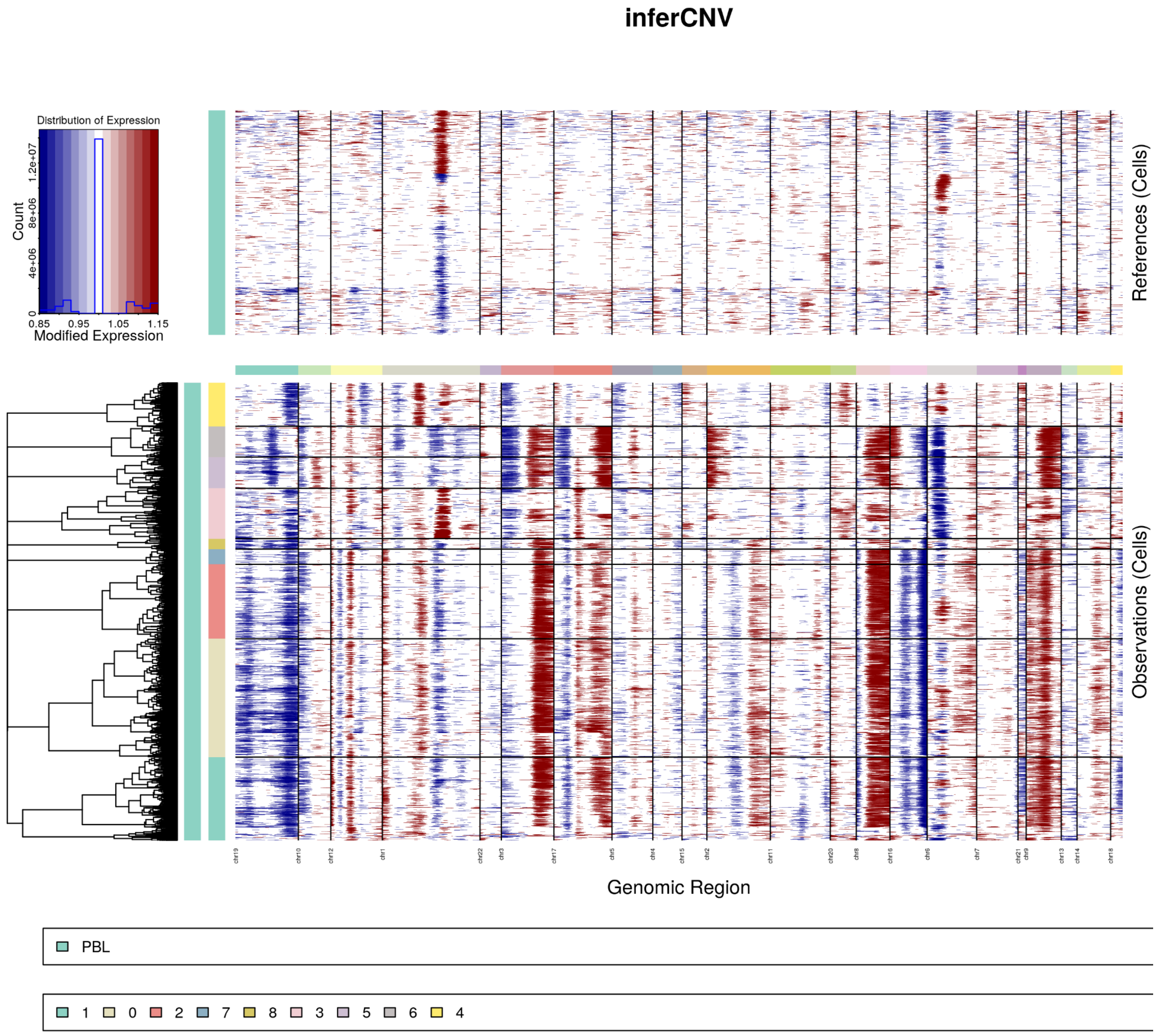

Comparison of copy number variation (CNV) profiles of CD45-negative epithelial cell clusters to the reference/baseline PBL (peripheral blood leukocytes) cells from scRNAseq data, similarly as it was done by Kürten et al.[8]. Based on these results, epithelial clusters 3 and 4 were annotated as non-malignant subsets. Only HPV-negative patients are included.

Supplementary Figure 18: PCA of the KEGG metabolic pathways GSVA enrichment scores of the LMU-KKG 08-13 and LMU-KKG 13-16 data sets.

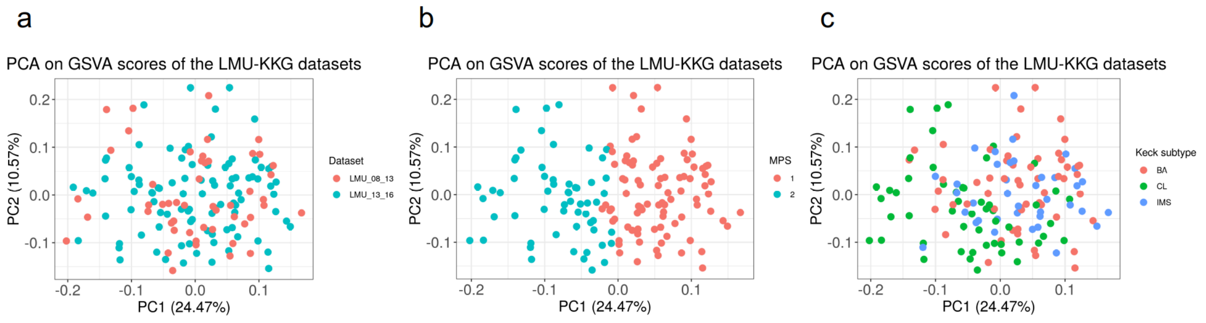

PCA was performed on the KEGG metabolic GSVA enrichment scores of the LMU-KKG 08-13 (n=50), and LMU-KKG 13-16 (n=95) HPV-negative subset cases together. Coloring by cohort (**a**), coloring by MPS class (**b**), and coloring by Keck subtype[9] (**c**). BA: basal, CL: classical, IMS: inflamed/mesenchymal.

References

1. Tibshirani, R., Hastie, T., Narasimhan, B. & Chu, G. Diagnosis of multiple cancer types by shrunken centroids of gene expression. *Proc. Natl. Acad. Sci.* **99**, 6567–6572 (2002).
2. Puram, S. V. *et al.* Single-Cell Transcriptomic Analysis of Primary and Metastatic Tumor Ecosystems in Head and Neck Cancer. *Cell* **171**, 1611-1624.e24 (2017).
3. Kanehisa, M. & Goto, S. KEGG: Kyoto Encyclopedia of Genes and Genomes. *Nucleic Acids Res.* **28**, 27–30 (2000).
4. Kanehisa, M., Furumichi, M., Sato, Y., Ishiguro-Watanabe, M. & Tanabe, M. KEGG: integrating viruses and cellular organisms. *Nucleic Acids Res.* **49**, D545–D551 (2020).
5. Love, M. I., Huber, W. & Anders, S. Moderated estimation of fold change and dispersion for RNA-seq data with DESeq2. *Genome Biol.* **15**, 550 (2014).
6. Chakravarthy, A. *et al.* Human Papillomavirus Drives Tumor Development Throughout the Head and Neck: Improved Prognosis Is Associated With an Immune Response Largely Restricted to the Oropharynx. *J. Clin. Oncol.* **34**, 4132–4141 (2016).
7. Schrank, T. P. *et al.* Noncanonical HPV carcinogenesis drives radiosensitization of head and neck tumors. *Proc. Natl. Acad. Sci.* **120**, e2216532120 (2023).
8. Kürten, C. H. L. *et al.* Investigating immune and non-immune cell interactions in head and neck tumors by single-cell RNA sequencing. *Nat. Commun.* **12**, 7338 (2021).
9. Keck, M. K. *et al.* Integrative Analysis of Head and Neck Cancer Identifies Two Biologically Distinct HPV and Three Non-HPV Subtypes. *Clin. Cancer Res.* **21**, 870–881 (2015).

Supplementary Figures HE slides

LMU HPV-negative MPS1 CS/DS metabolism high cases

Black arrow: malignant cells, grey arrow: CAFs

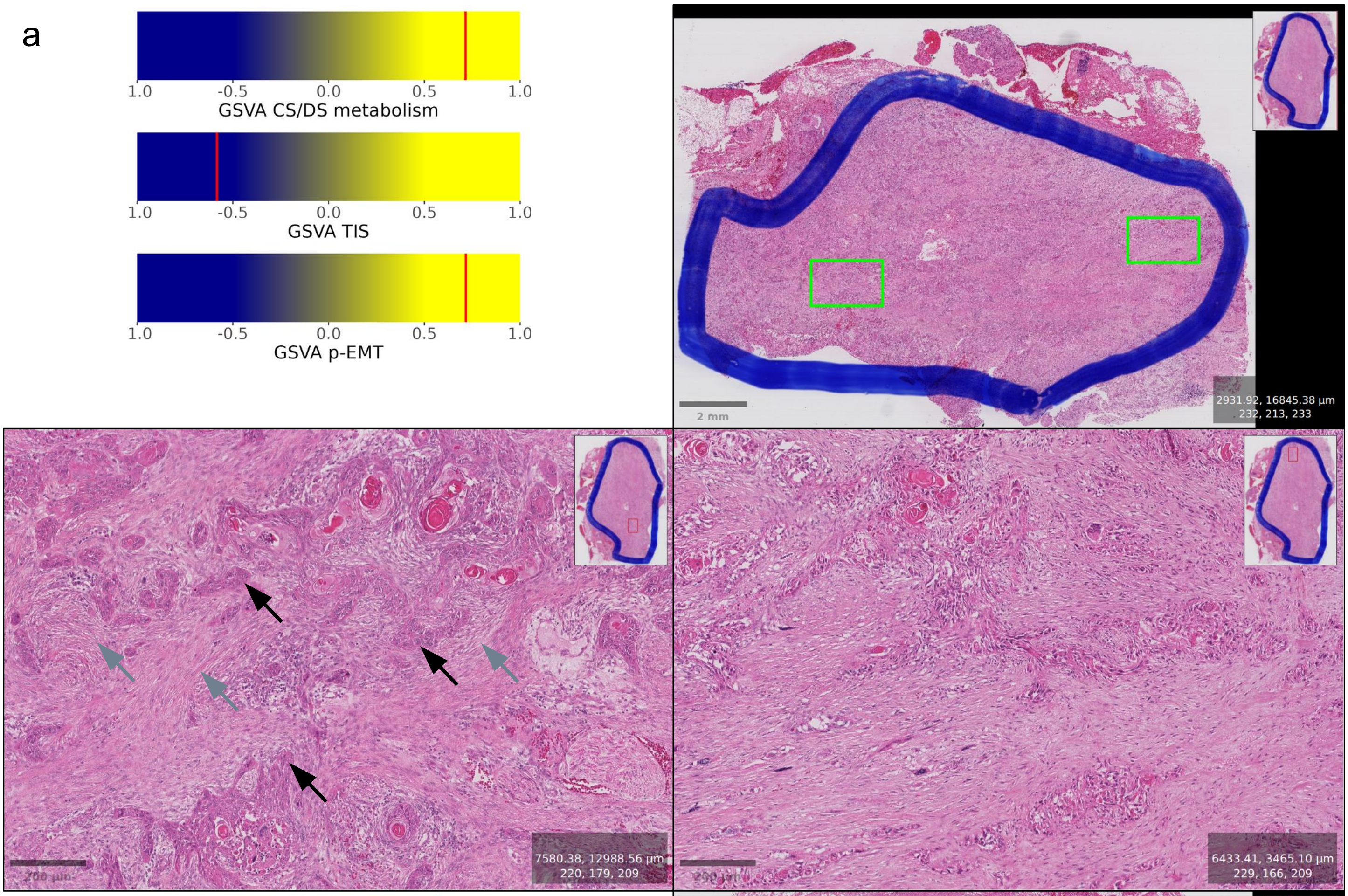

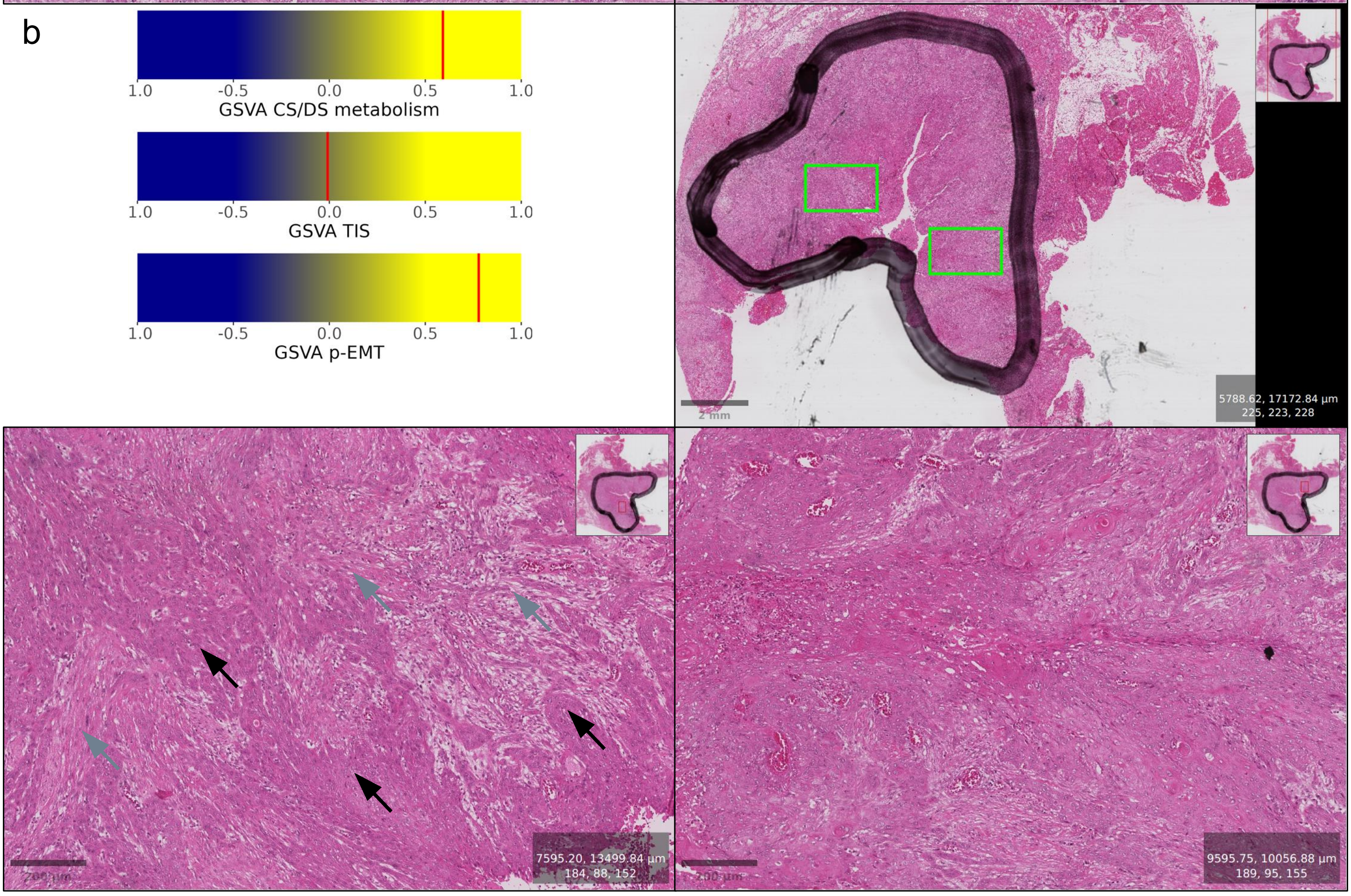

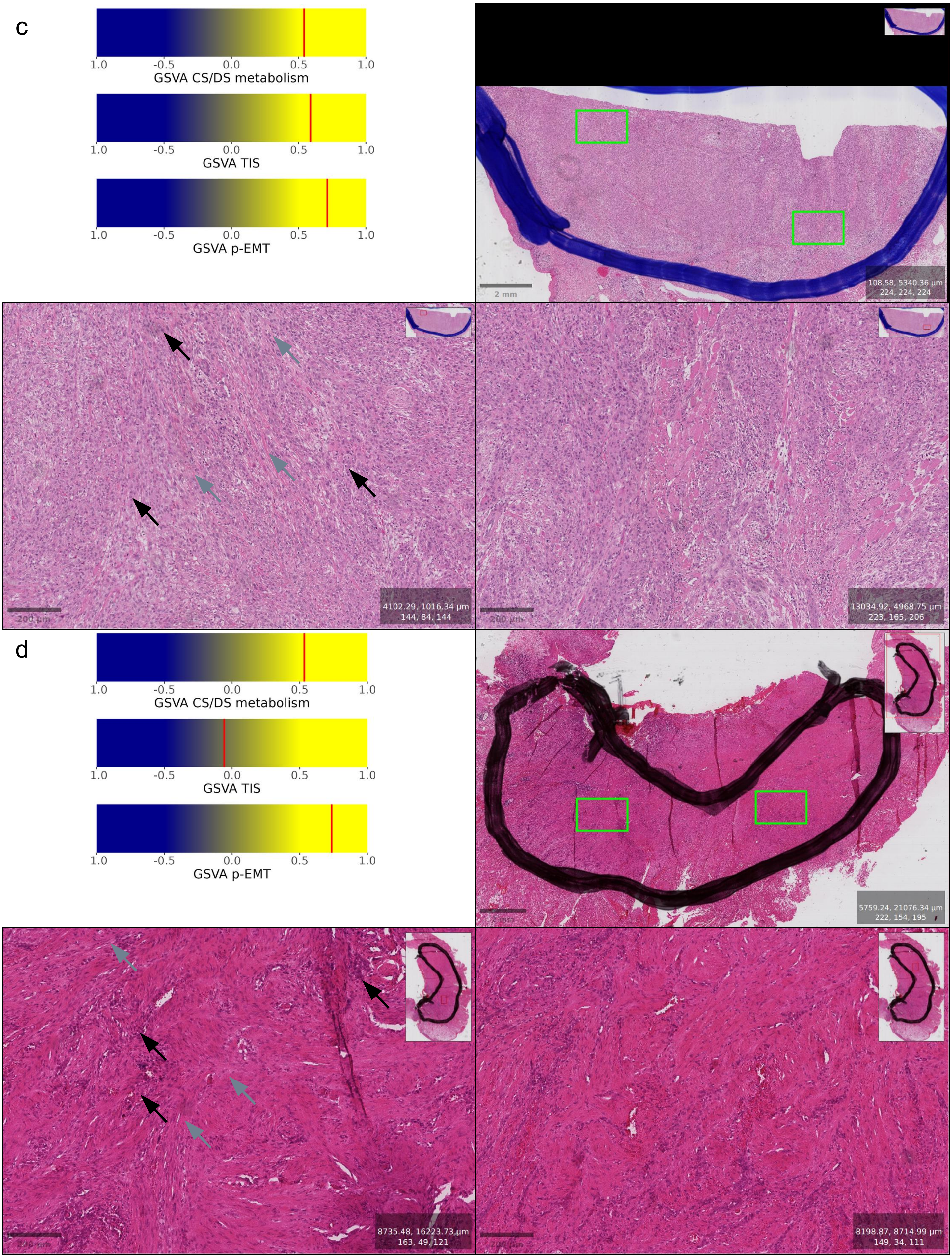
c
1.0 -0.5 0.0 0.5 1.0
GSVA CS/DS metabolism
1.0 -0.5 0.0 0.5 1.0
GSVA TIS
1.0 -0.5 0.0 0.5 1.0
GSVA p-EMT
2 mm
108.58, 5340.36 µm
224, 224, 224
4102.29, 1016.34 µm
144, 84, 144
13034.92, 4968.75 µm
223, 165, 206
d
1.0 -0.5 0.0 0.5 1.0
GSVA CS/DS metabolism
1.0 -0.5 0.0 0.5 1.0
GSVA TIS
1.0 -0.5 0.0 0.5 1.0
GSVA p-EMT
5759.24, 21076.34 µm
222, 154, 195
8735.48, 16223.73 µm
163, 49, 121
8198.87, 8714.99 µm
149, 34, 111

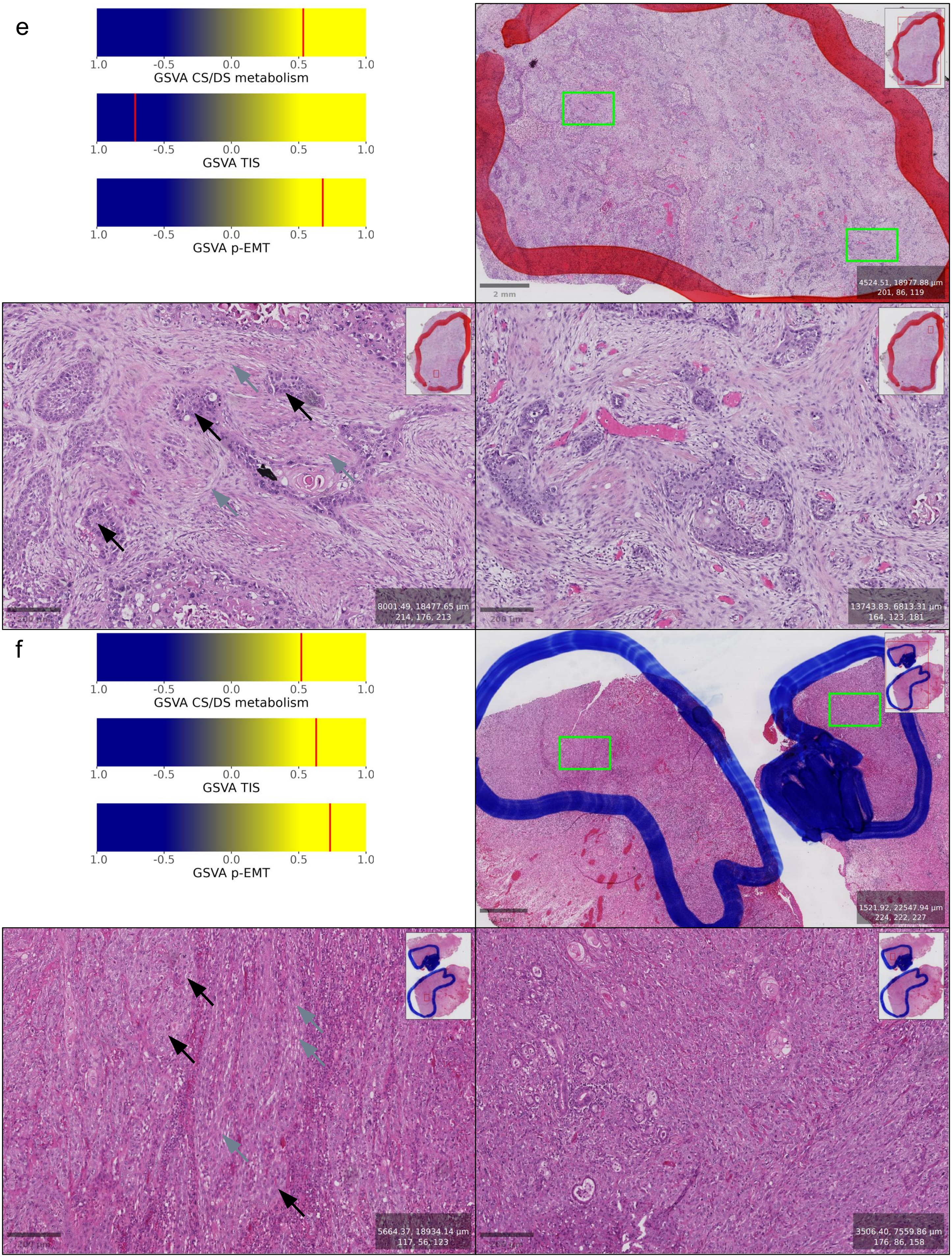

e
1.0 -0.5 0.0 0.5 1.0
GSVA CS/DS metabolism
1.0 -0.5 0.0 0.5 1.0
GSVA TIS
1.0 -0.5 0.0 0.5 1.0
GSVA p-EMT
2 mm
4524.51, 18977.88 µm
201, 86, 119
8001.49, 18477.65 µm
214, 176, 213
13743.83, 6813.31 µm
164, 123, 181
f
1.0 -0.5 0.0 0.5 1.0
GSVA CS/DS metabolism
1.0 -0.5 0.0 0.5 1.0
GSVA TIS
1.0 -0.5 0.0 0.5 1.0
GSVA p-EMT
1521.92, 22547.94 µm
224, 222, 227
5664.37, 18934.14 µm
117, 56, 123
3506.40, 7559.86 µm
176, 86, 158

g

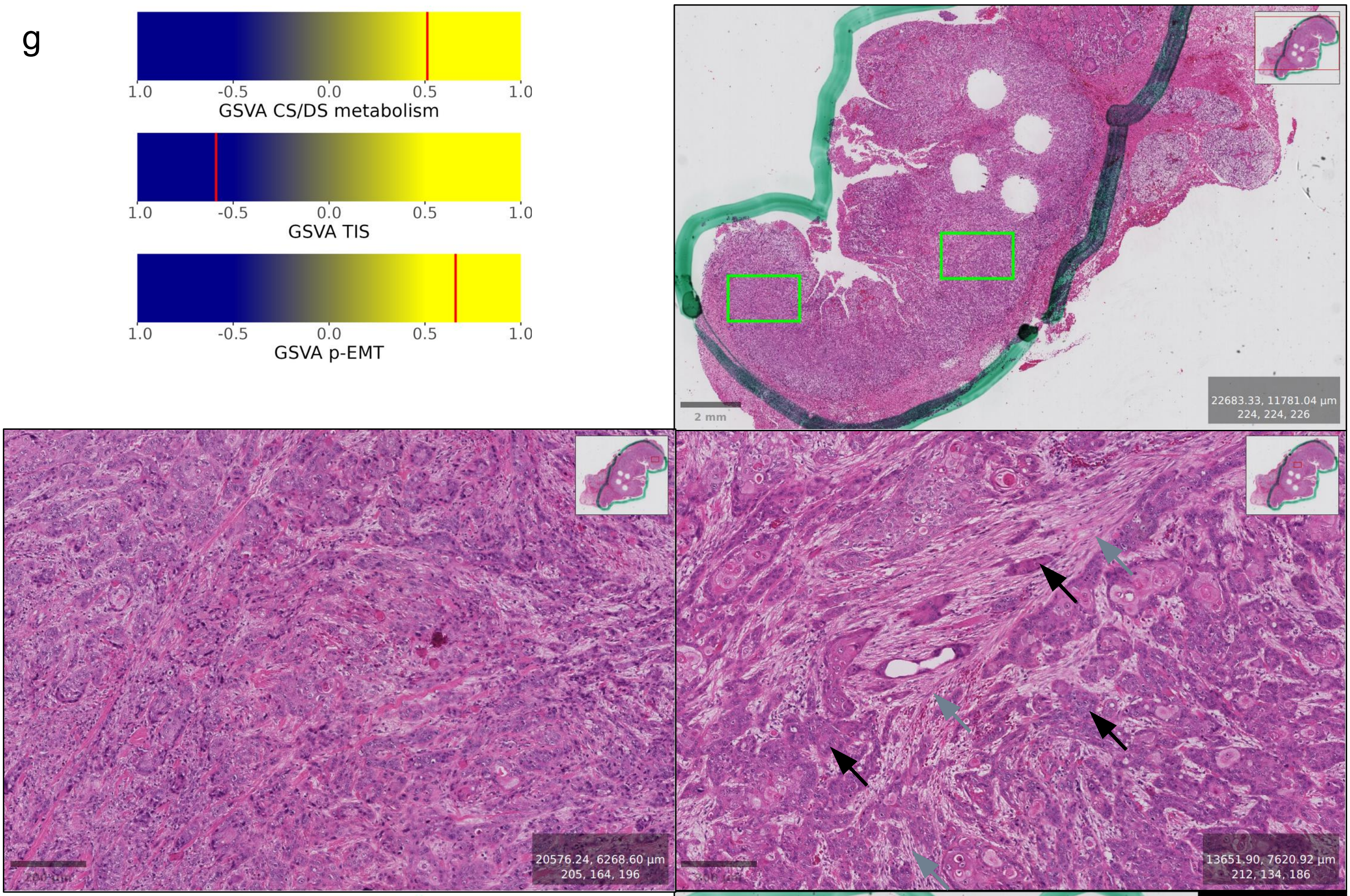
1.0 -0.5 0.0 0.5 1.0
GSVA CS/DS metabolism
1.0 -0.5 0.0 0.5 1.0
GSVA TIS
1.0 -0.5 0.0 0.5 1.0
GSVA p-EMT
2 mm
22683.33, 11781.04 µm
224, 224, 226
20576.24, 6268.60 µm
205, 164, 196
13651.90, 7620.92 µm
212, 134, 186

h

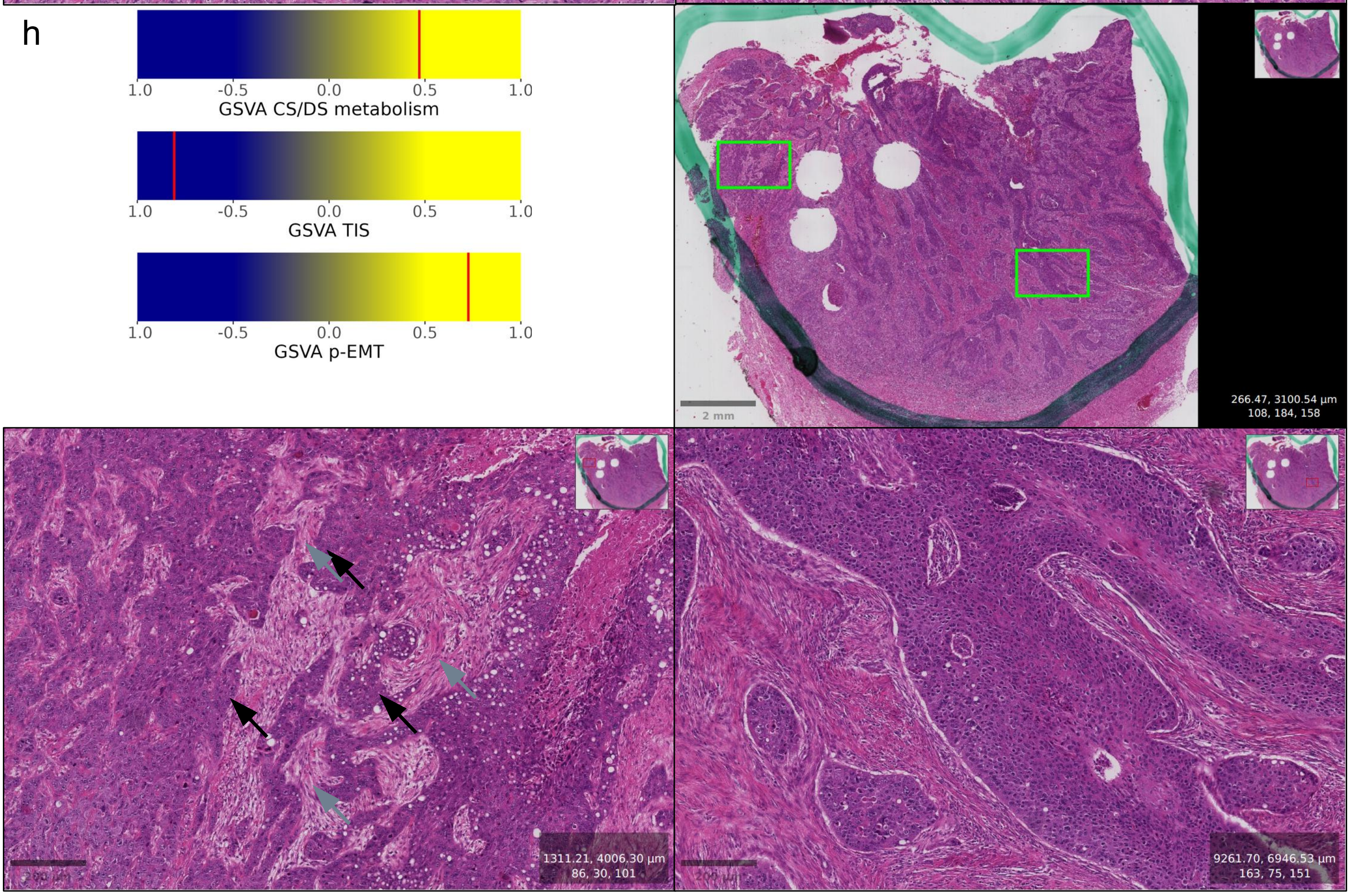
1.0 -0.5 0.0 0.5 1.0
GSVA CS/DS metabolism
1.0 -0.5 0.0 0.5 1.0
GSVA TIS
1.0 -0.5 0.0 0.5 1.0
GSVA p-EMT
2 mm
266.47, 3100.54 µm
108, 184, 158
1311.21, 4006.30 µm
86, 30, 101
9261.70, 6946.53 µm
163, 75, 151

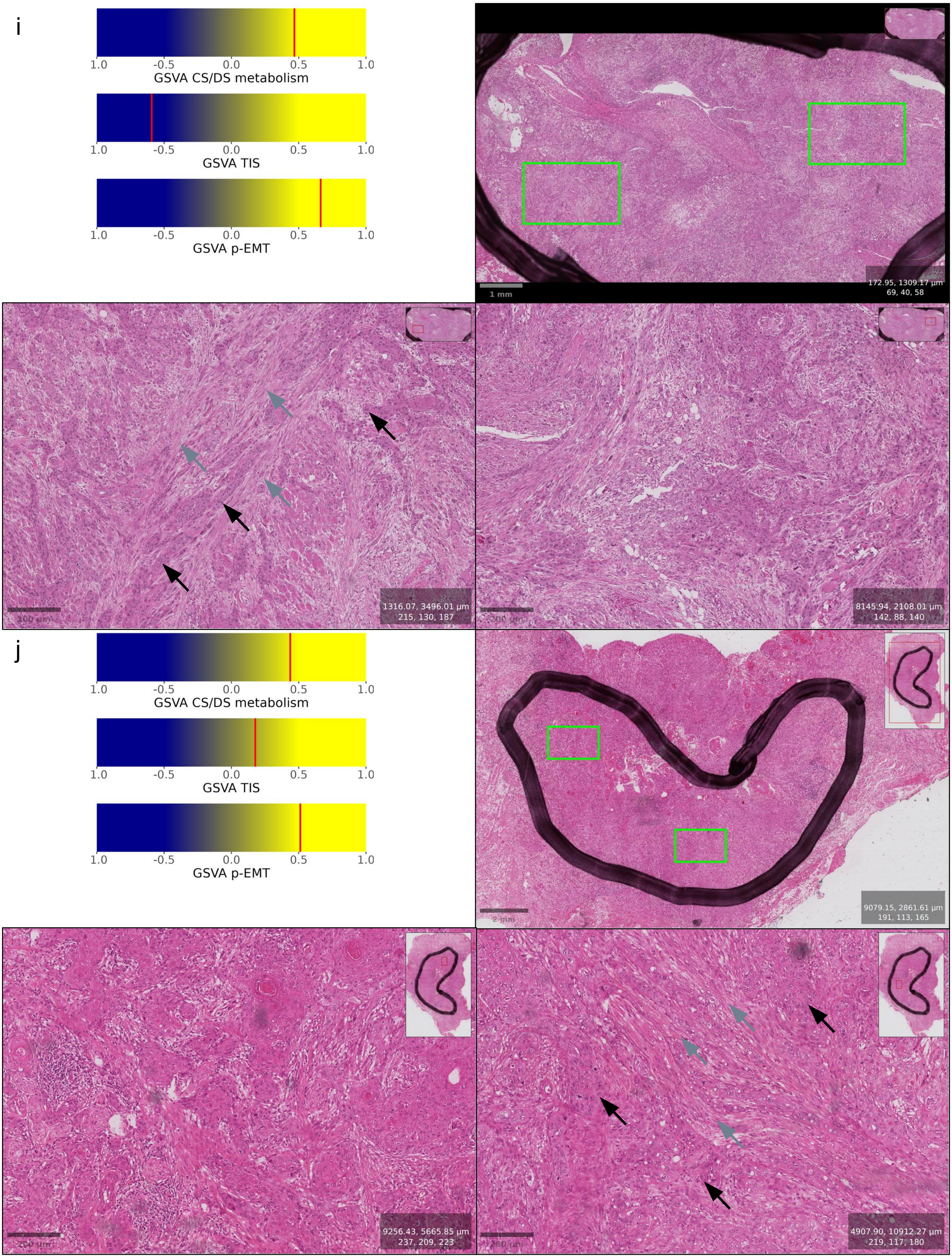
i
1.0 -0.5 0.0 0.5 1.0
GSVA CS/DS metabolism
1.0 -0.5 0.0 0.5 1.0
GSVA TIS
1.0 -0.5 0.0 0.5 1.0
GSVA p-EMT
1 mm
172.95, 1309.17 µm
69, 40, 58
1316.07, 3496.01 µm
215, 130, 187
8145.94, 2108.01 µm
142, 88, 140
j
1.0 -0.5 0.0 0.5 1.0
GSVA CS/DS metabolism
1.0 -0.5 0.0 0.5 1.0
GSVA TIS
1.0 -0.5 0.0 0.5 1.0
GSVA p-EMT
9079.15, 2861.61 µm
191, 113, 165
9256.43, 5665.85 µm
237, 209, 223
4907.90, 10912.27 µm
219, 117, 180

LMU HPV-negative MPS2 CS/DS low cases

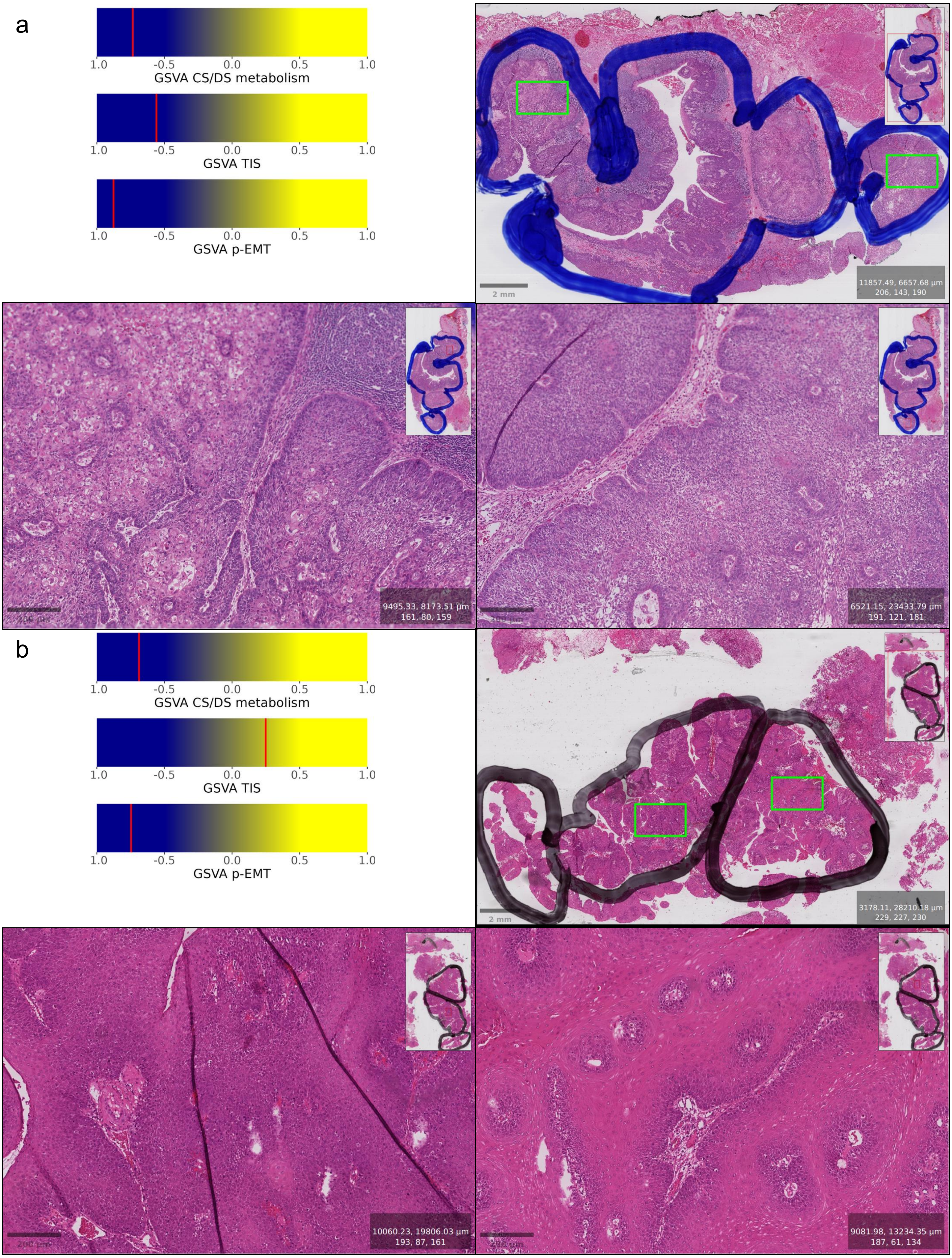

c

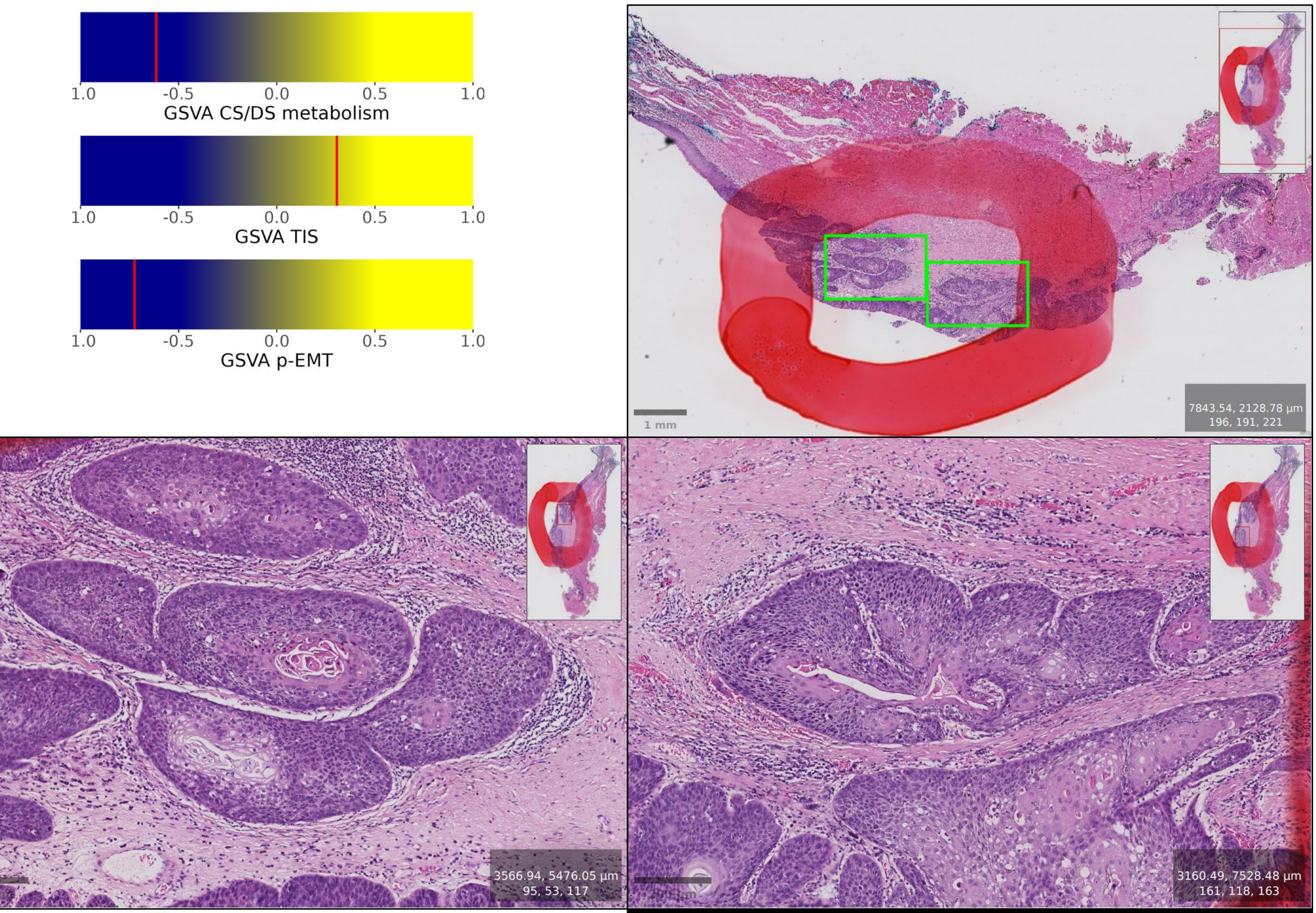
1.0 -0.5 0.0 0.5 1.0
GSVA CS/DS metabolism
1.0 -0.5 0.0 0.5 1.0
GSVA TIS
1.0 -0.5 0.0 0.5 1.0
GSVA p-EMT
1 mm
7843.54, 2128.78 μm
196, 191, 221
3566.94, 5476.05 μm
95, 53, 117
3160.49, 7528.48 μm
161, 118, 163

d

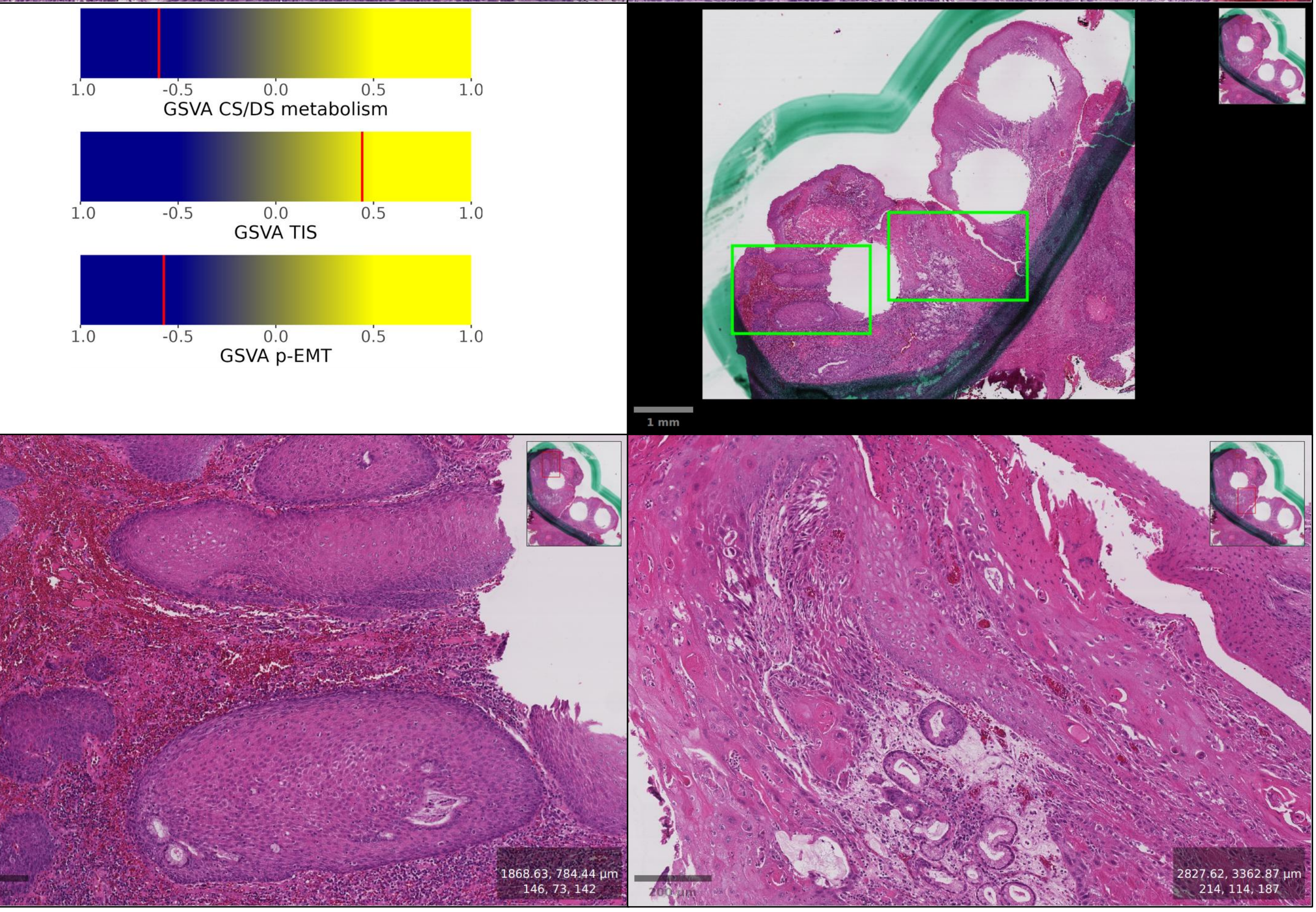
1.0 -0.5 0.0 0.5 1.0
GSVA CS/DS metabolism
1.0 -0.5 0.0 0.5 1.0
GSVA TIS
1.0 -0.5 0.0 0.5 1.0
GSVA p-EMT
1 mm
1868.63, 784.44 μm
146, 73, 142
2827.62, 3362.87 μm
214, 114, 187

e

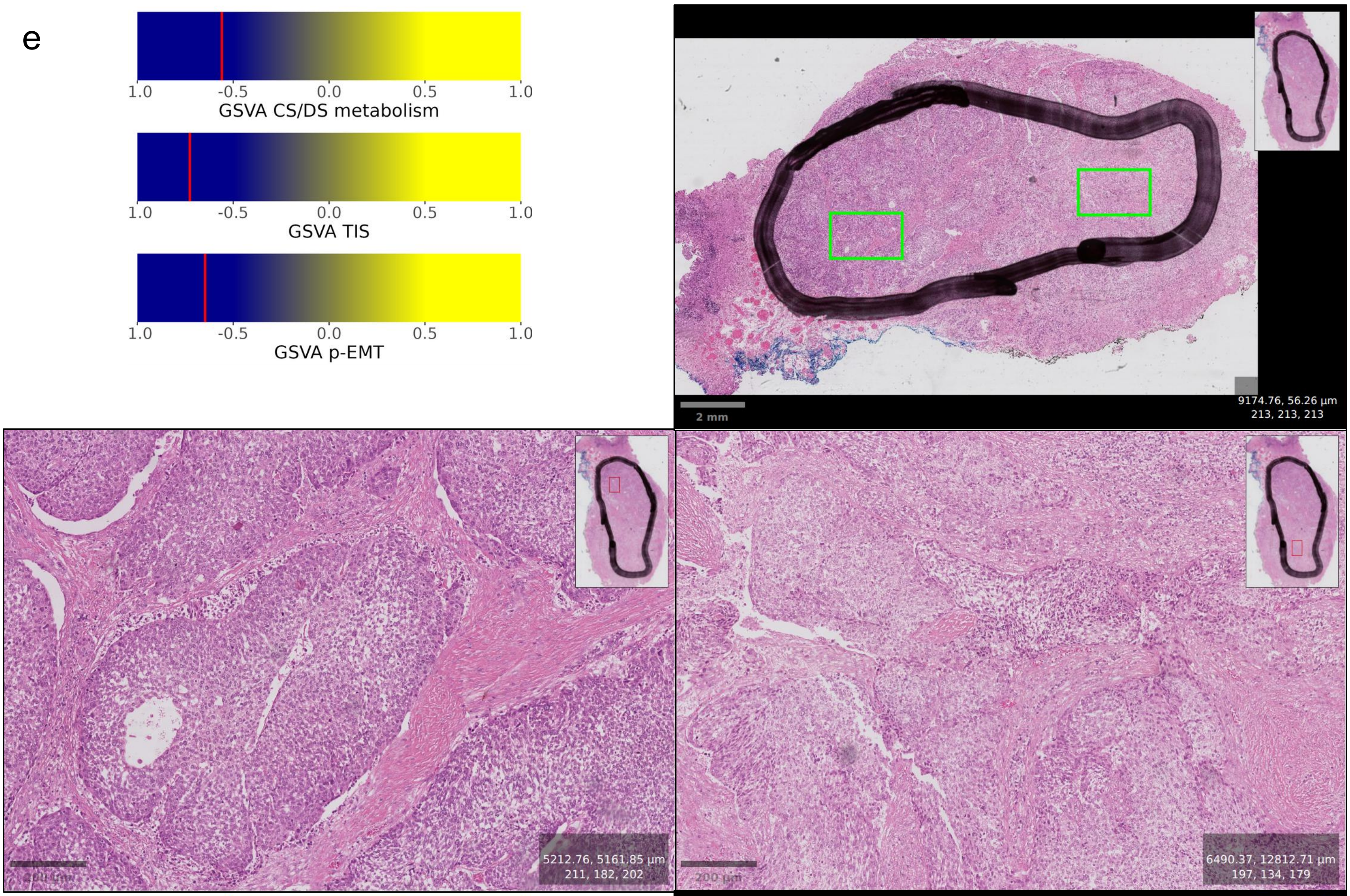

1.0 -0.5 0.0 0.5 1.0
GSVA CS/DS metabolism
1.0 -0.5 0.0 0.5 1.0
GSVA TIS
1.0 -0.5 0.0 0.5 1.0
GSVA p-EMT
2 mm
9174.76, 56.26 µm
213, 213, 213
5212.76, 5161.85 µm
211, 182, 202
6490.37, 12812.71 µm
197, 134, 179

f

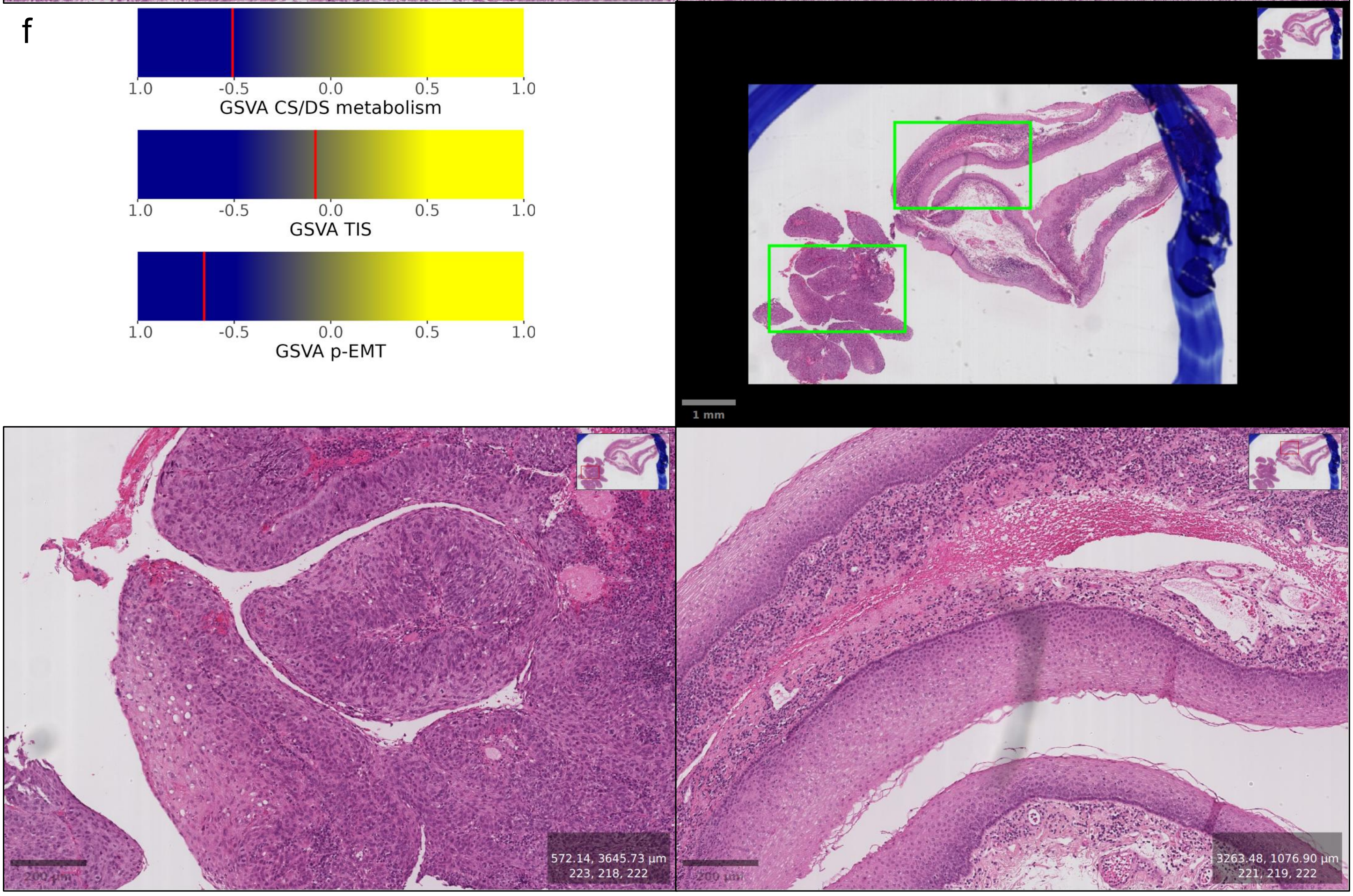

1.0 -0.5 0.0 0.5 1.0
GSVA CS/DS metabolism
1.0 -0.5 0.0 0.5 1.0
GSVA TIS
1.0 -0.5 0.0 0.5 1.0
GSVA p-EMT
1 mm
572.14, 3645.73 µm
223, 218, 222
3263.48, 1076.90 µm
221, 219, 222

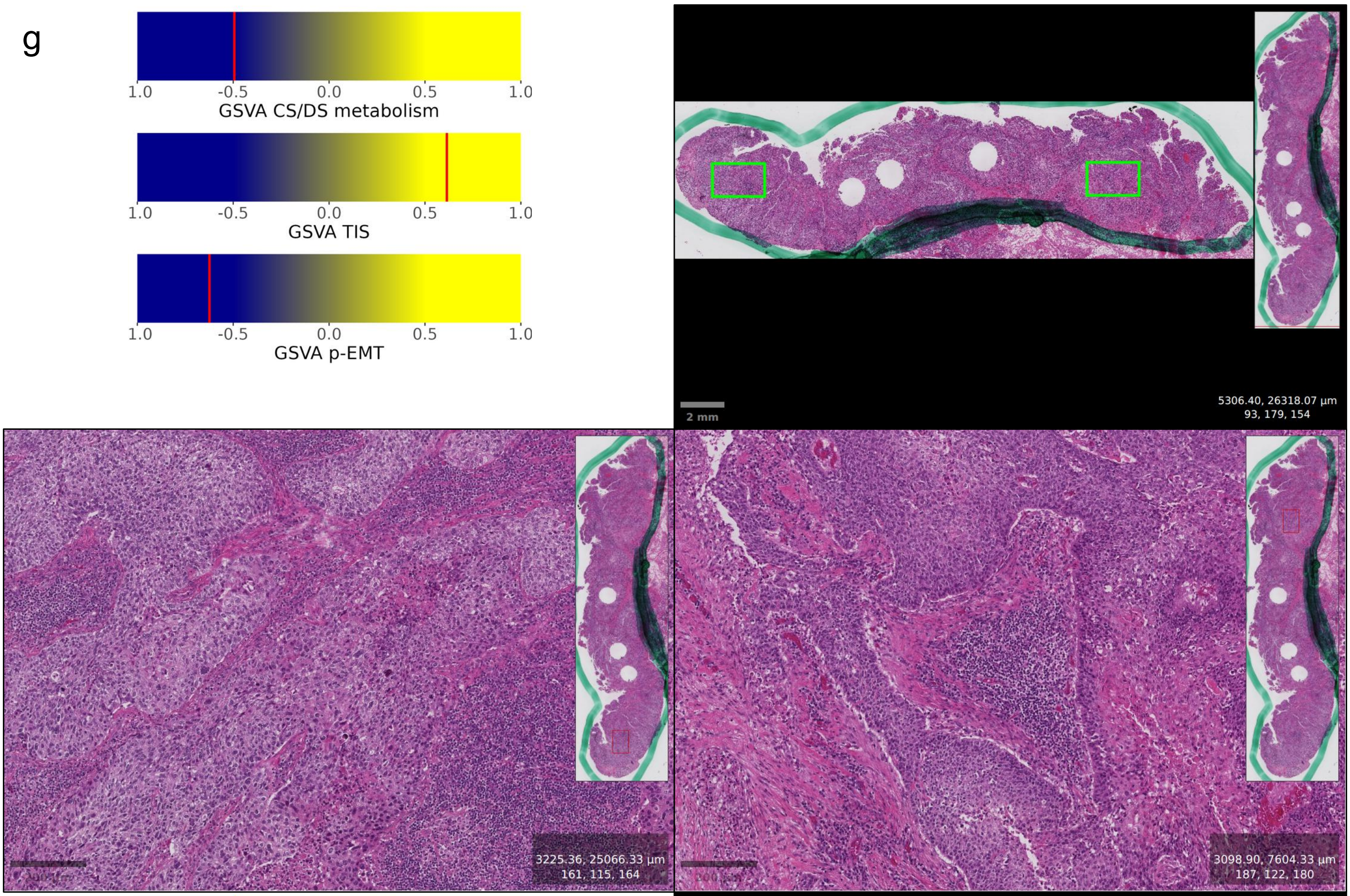
g
1.0
-0.5
0.0
0.5
1.0
GSVA CS/DS metabolism
GSVA TIS
GSVA p-EMT
2 mm
5306.40, 26318.07 µm
93, 179, 154
3225.36, 25066.33 µm
161, 115, 164
3098.90, 7604.33 µm
187, 122, 180

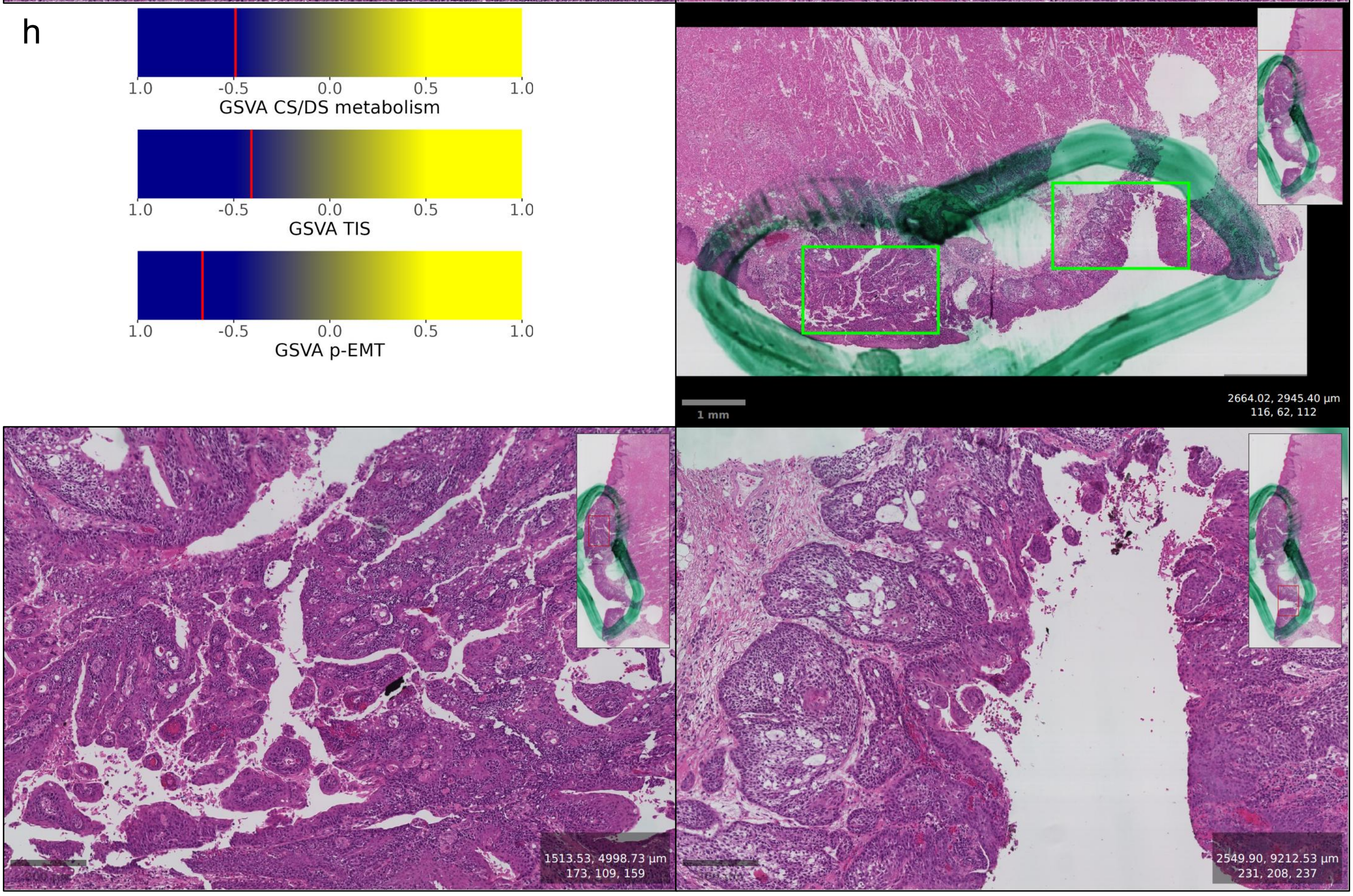
h
1.0
-0.5
0.0
0.5
1.0
GSVA CS/DS metabolism
GSVA TIS
GSVA p-EMT
1 mm
2664.02, 2945.40 µm
116, 62, 112
1513.53, 4998.73 µm
173, 109, 159
2549.90, 9212.53 µm
231, 208, 237

i

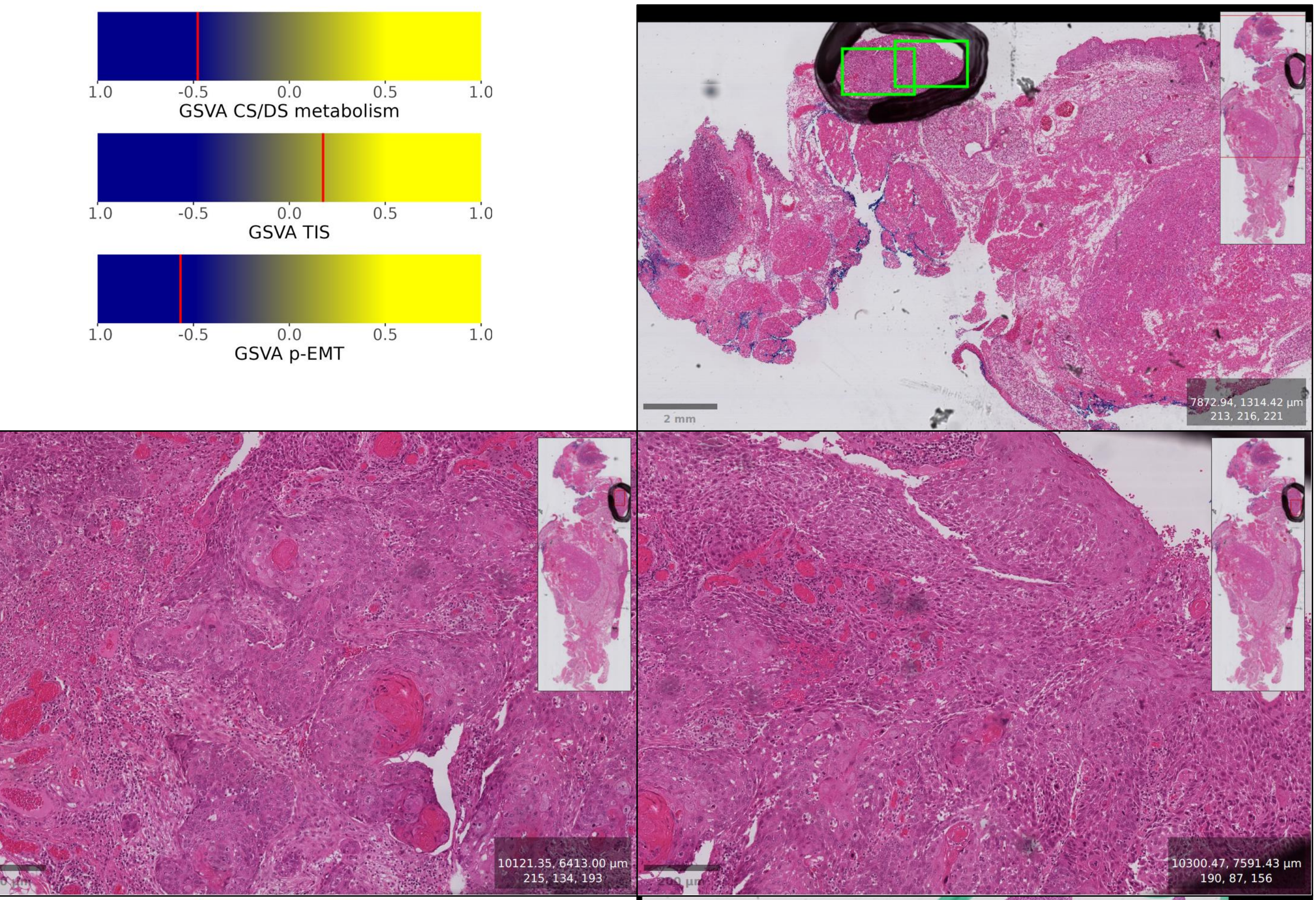
1.0 -0.5 0.0 0.5 1.0
GSVA CS/DS metabolism
1.0 -0.5 0.0 0.5 1.0
GSVA TIS
1.0 -0.5 0.0 0.5 1.0
GSVA p-EMT
2 mm
7872.94, 1314.42 µm
213, 216, 221
10121.35, 6413.00 µm
215, 134, 193
10300.47, 7591.43 µm
190, 87, 156

j

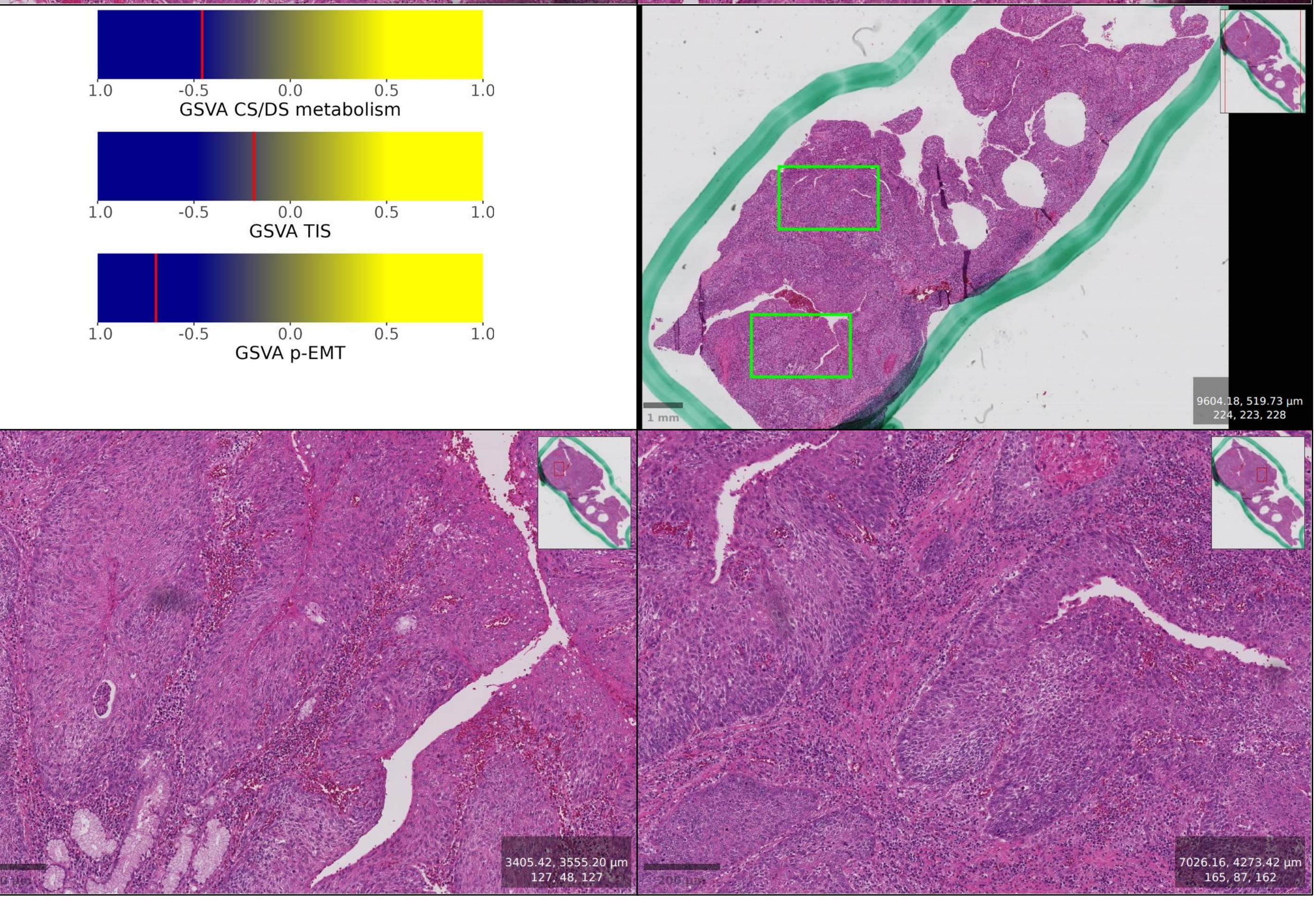
1.0 -0.5 0.0 0.5 1.0
GSVA CS/DS metabolism
1.0 -0.5 0.0 0.5 1.0
GSVA TIS
1.0 -0.5 0.0 0.5 1.0
GSVA p-EMT
1 mm
9604.18, 519.73 µm
224, 223, 228
3405.42, 3555.20 µm
127, 48, 127
7026.16, 4273.42 µm
165, 87, 162

TCGA HPV-negative MPS1 GSVA CS/DS high cases

Black arrow: malignant cells, grey arrow: CAFs

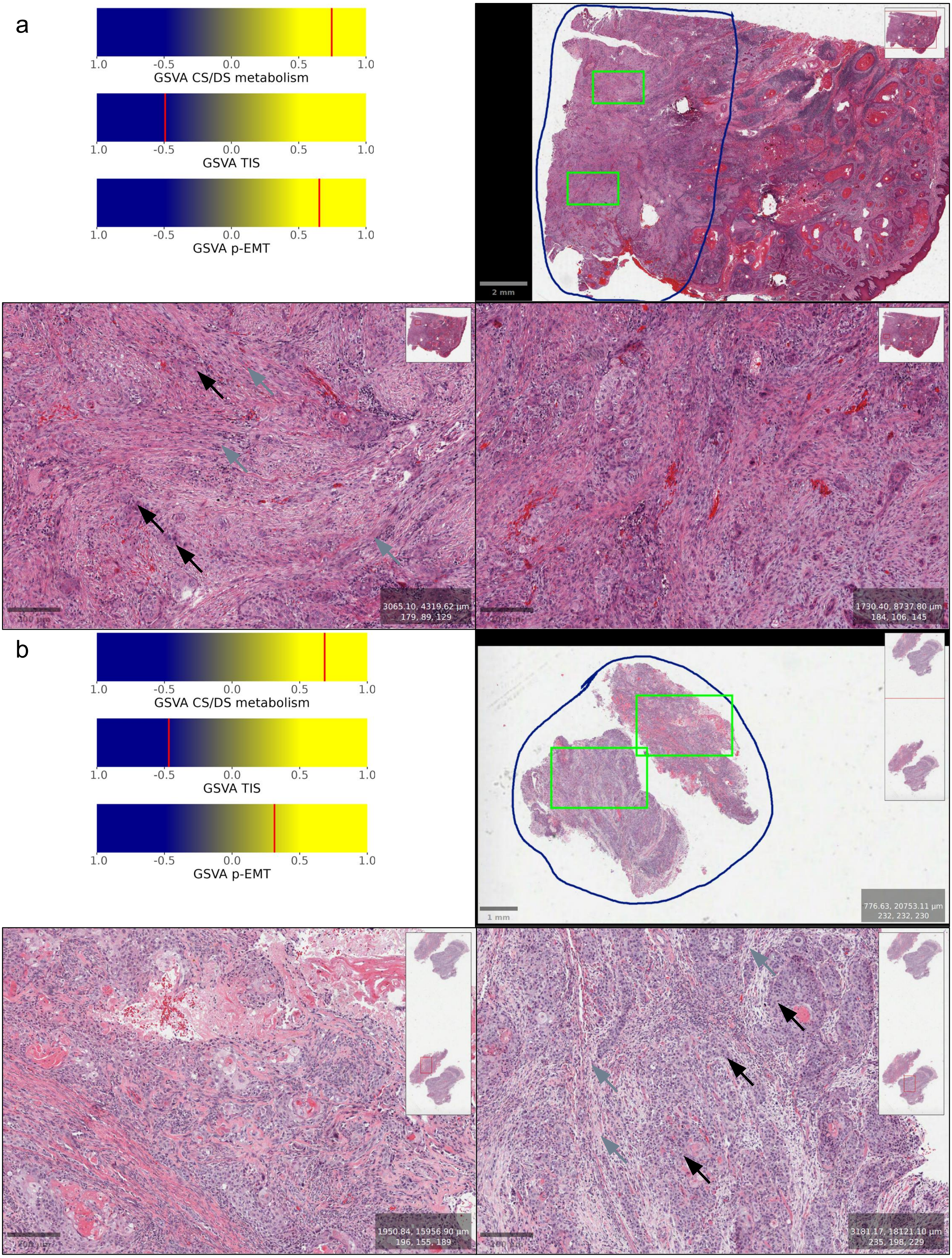

c

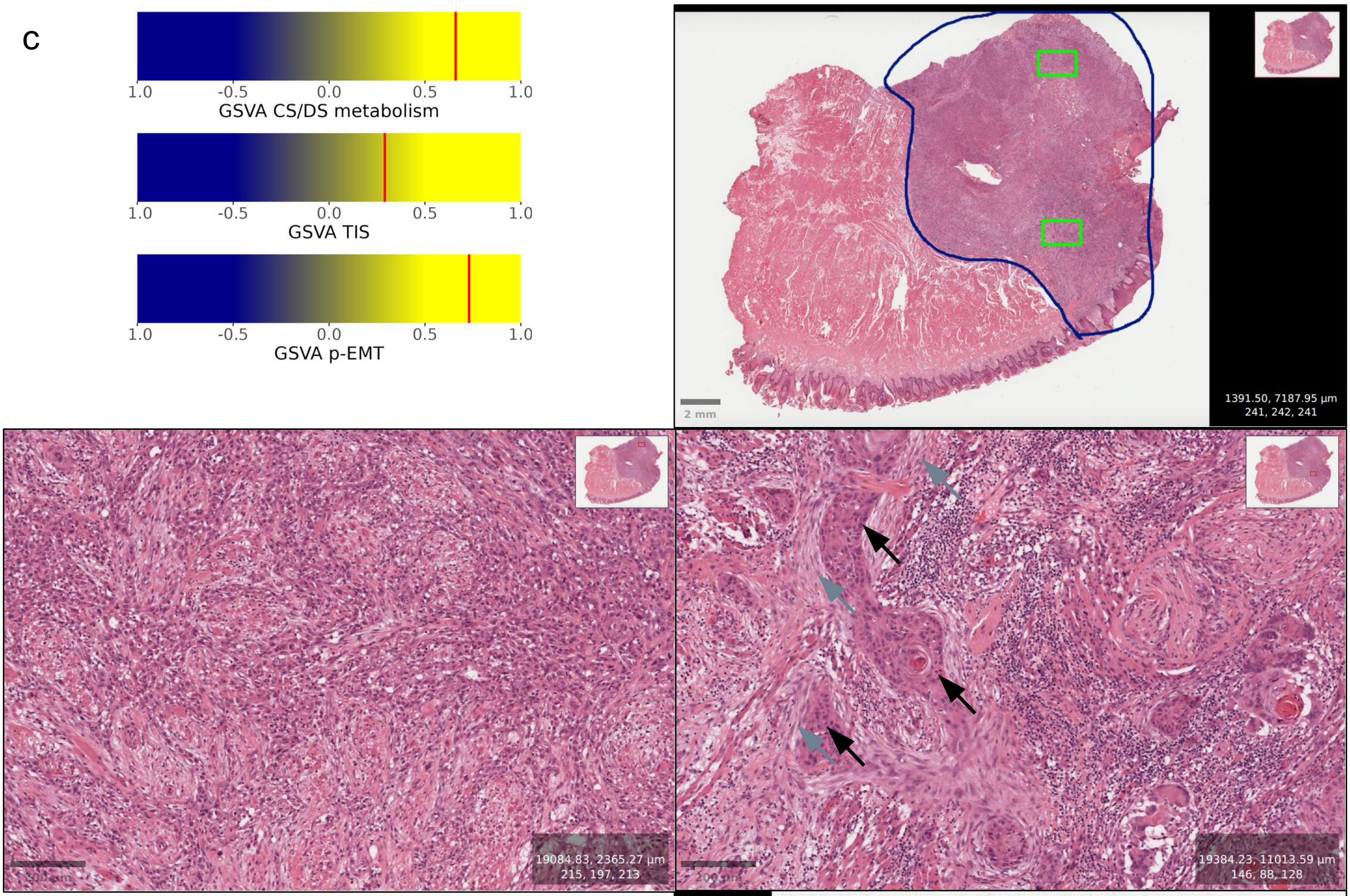

1.0 -0.5 0.0 0.5 1.0
GSVA CS/DS metabolism
1.0 -0.5 0.0 0.5 1.0
GSVA TIS
1.0 -0.5 0.0 0.5 1.0
GSVA p-EMT
2 mm
1391.50, 7187.95 µm
241, 242, 241
19084.83, 2365.27 µm
215, 197, 213
19384.23, 11013.59 µm
146, 88, 128

d

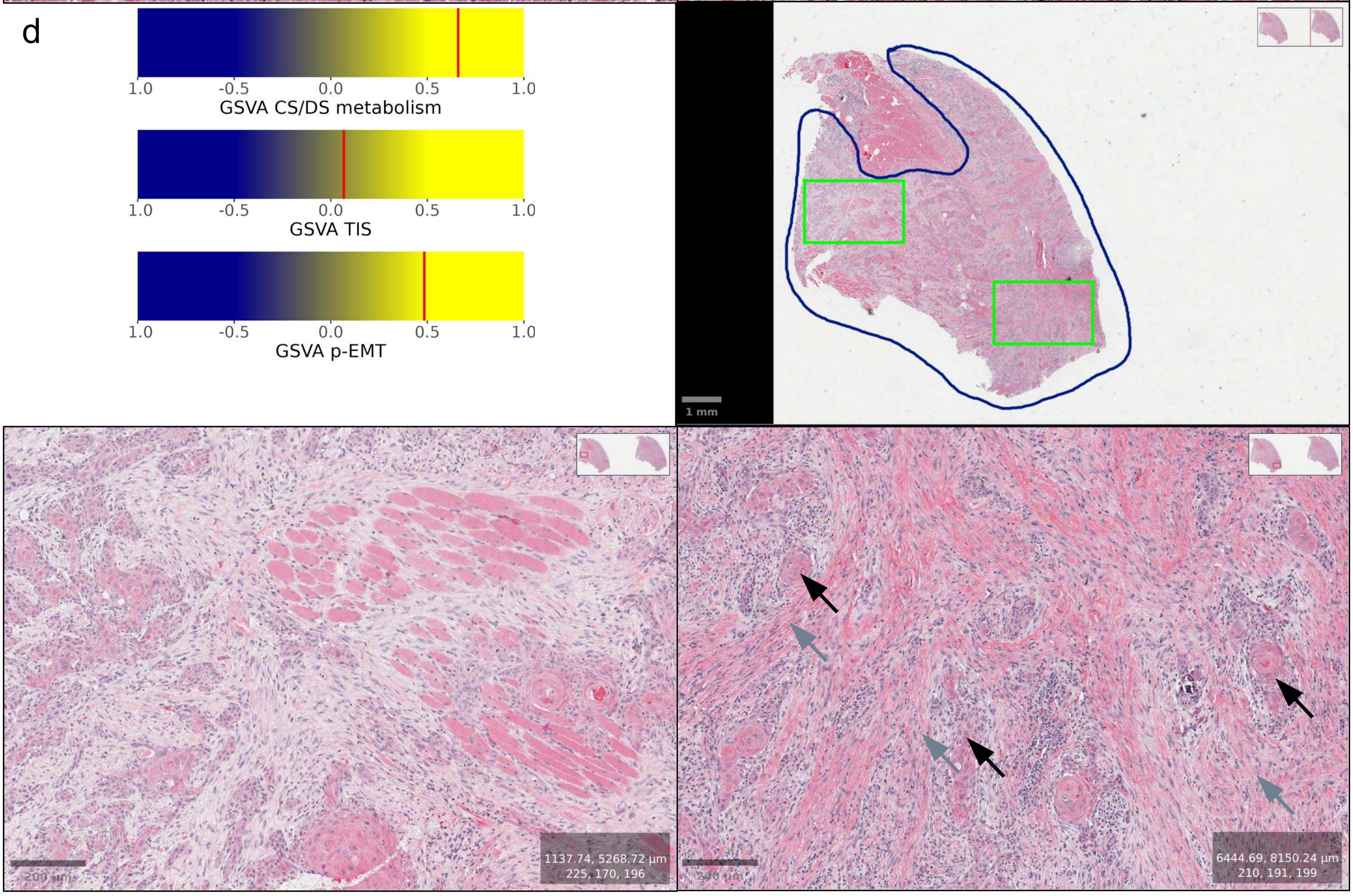

1.0 -0.5 0.0 0.5 1.0
GSVA CS/DS metabolism
1.0 -0.5 0.0 0.5 1.0
GSVA TIS
1.0 -0.5 0.0 0.5 1.0
GSVA p-EMT
1 mm
1137.74, 5268.72 µm
225, 170, 196
6444.69, 8150.24 µm
210, 191, 199

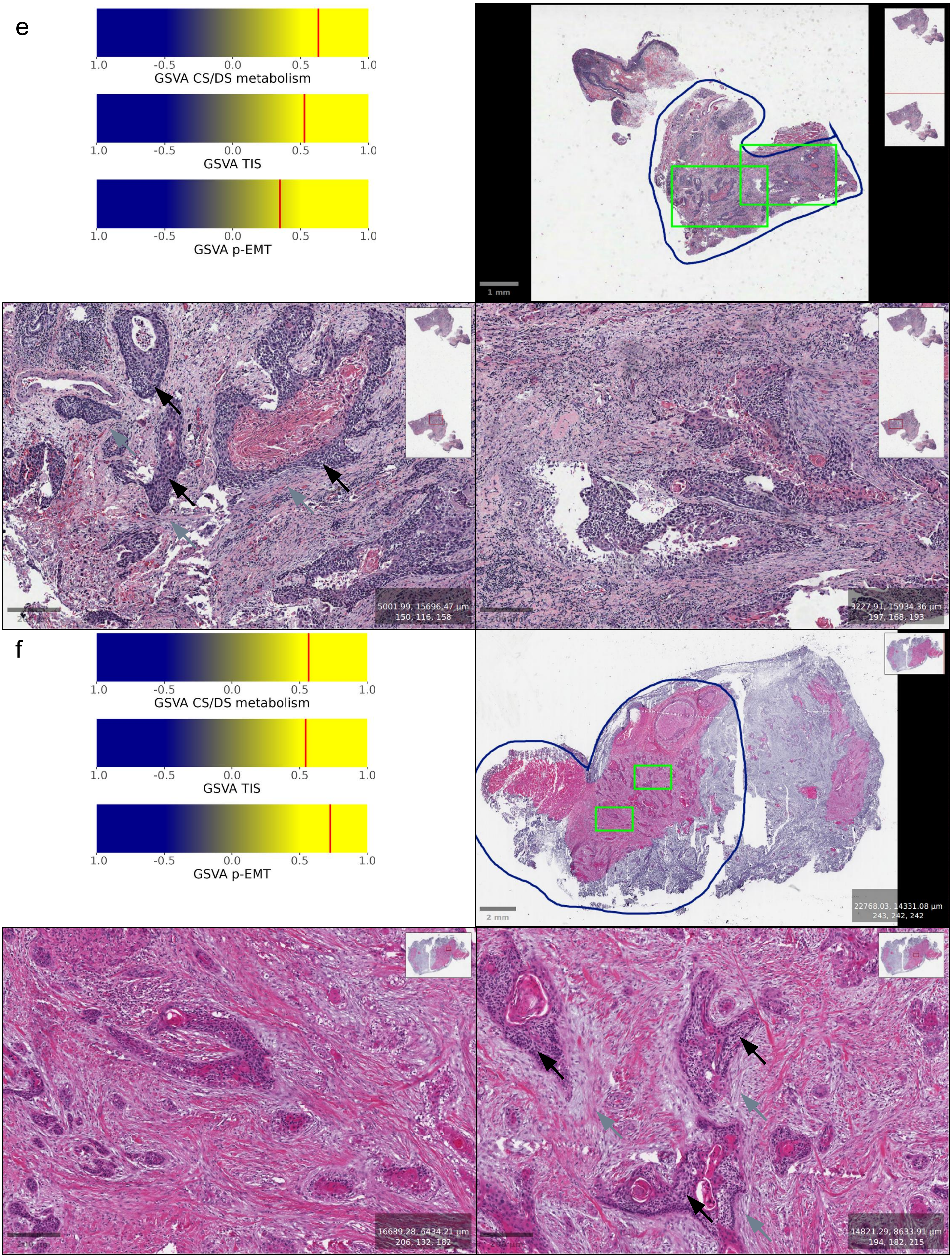

e
1.0 -0.5 0.0 0.5 1.0
GSVA CS/DS metabolism
1.0 -0.5 0.0 0.5 1.0
GSVA TIS
1.0 -0.5 0.0 0.5 1.0
GSVA p-EMT
1 mm
5001.99, 15696.47 µm
150, 116, 158
3227.91, 15934.36 µm
197, 168, 193
f
1.0 -0.5 0.0 0.5 1.0
GSVA CS/DS metabolism
1.0 -0.5 0.0 0.5 1.0
GSVA TIS
1.0 -0.5 0.0 0.5 1.0
GSVA p-EMT
2 mm
22768.03, 14331.08 µm
243, 242, 242
16689.28, 6434.21 µm
206, 132, 182
14821.29, 8633.91 µm
194, 182, 215

g

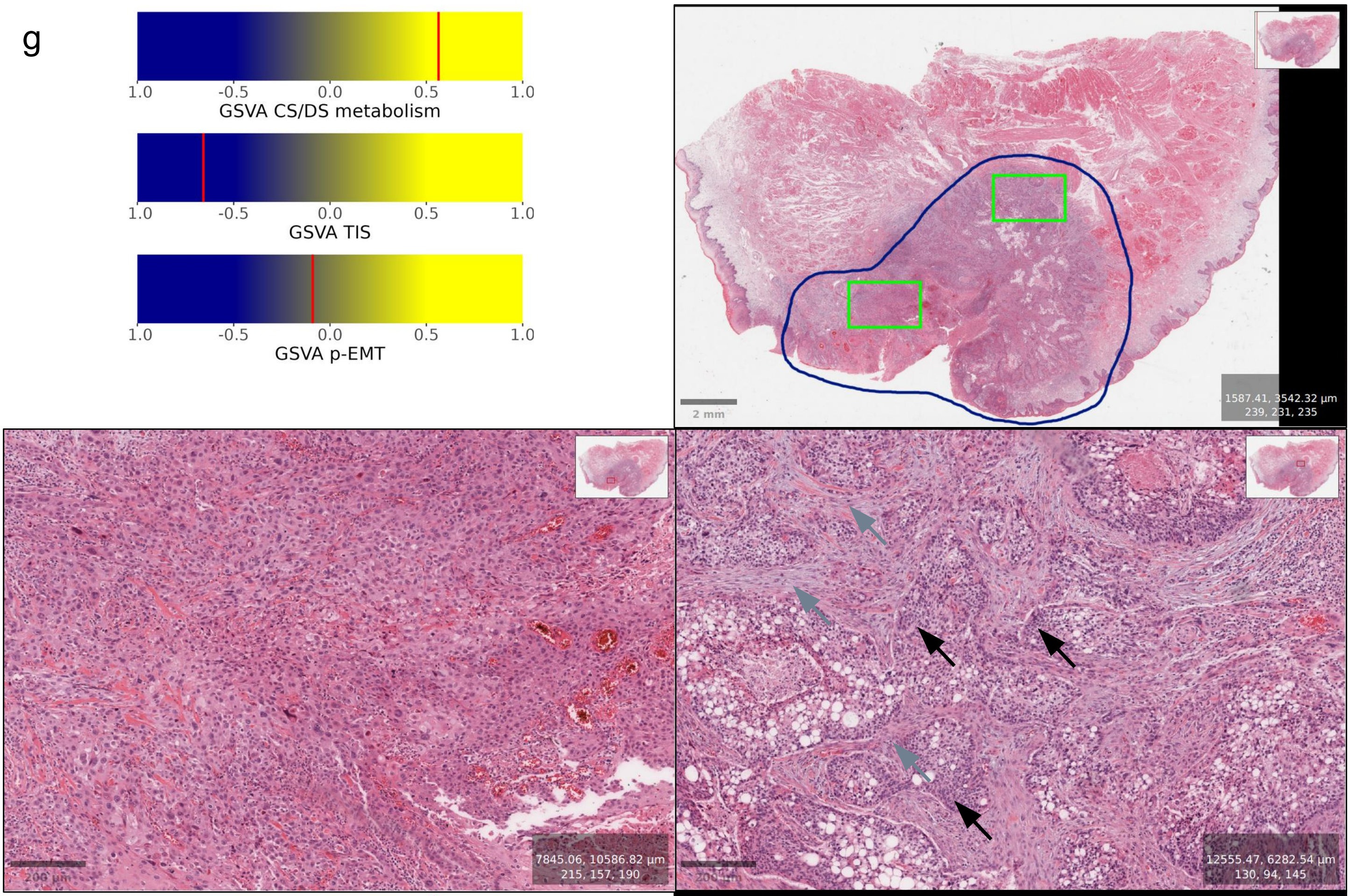
1.0 -0.5 0.0 0.5 1.0
GSVA CS/DS metabolism
1.0 -0.5 0.0 0.5 1.0
GSVA TIS
1.0 -0.5 0.0 0.5 1.0
GSVA p-EMT
2 mm
1587.41, 3542.32 µm
239, 231, 235
7845.06, 10586.82 µm
215, 157, 190
12555.47, 6282.54 µm
130, 94, 145

h

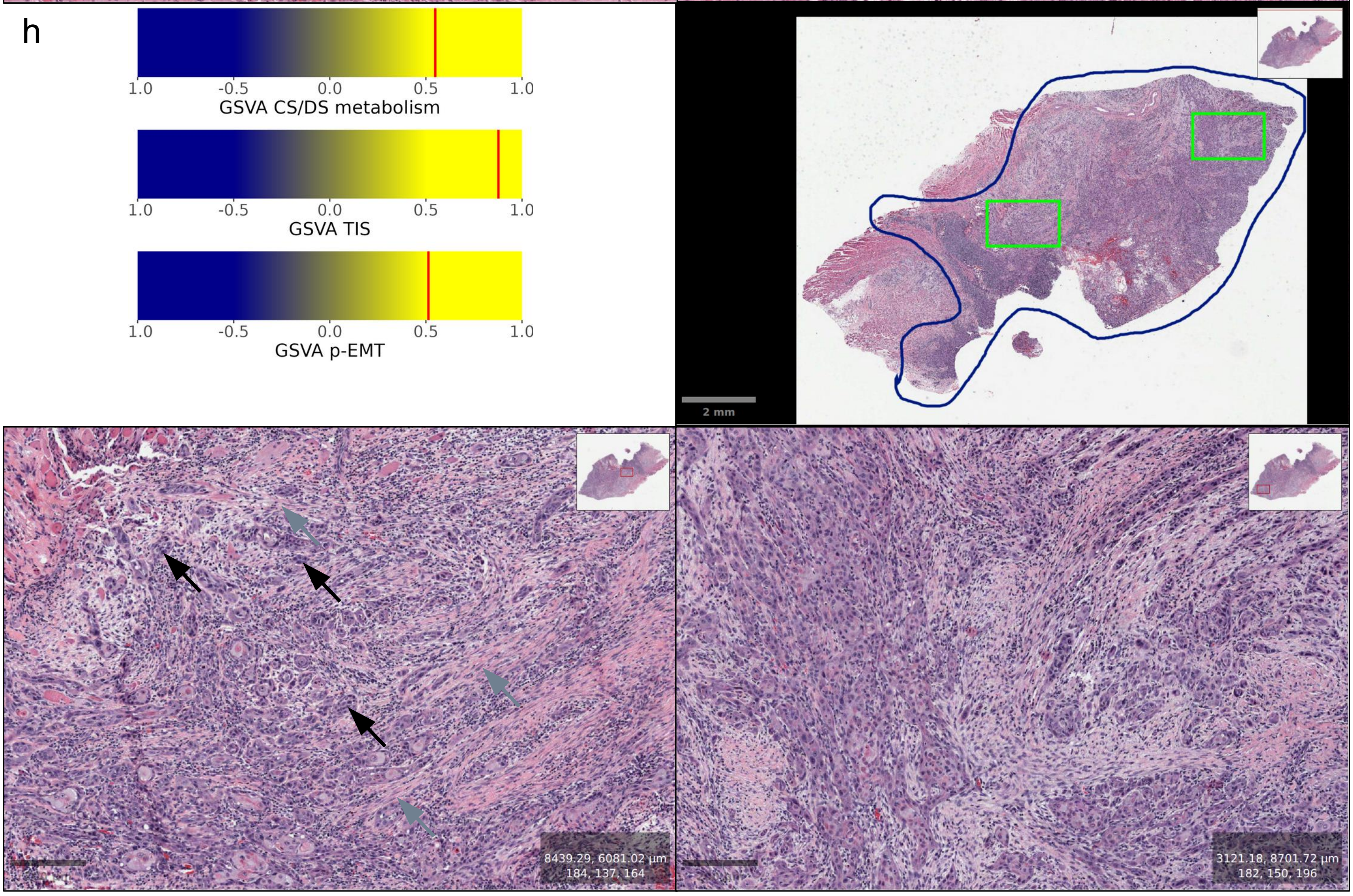
1.0 -0.5 0.0 0.5 1.0
GSVA CS/DS metabolism
1.0 -0.5 0.0 0.5 1.0
GSVA TIS
1.0 -0.5 0.0 0.5 1.0
GSVA p-EMT
2 mm
8439.29, 6081.02 µm
184, 137, 164
3121.18, 8701.72 µm
182, 150, 196

i

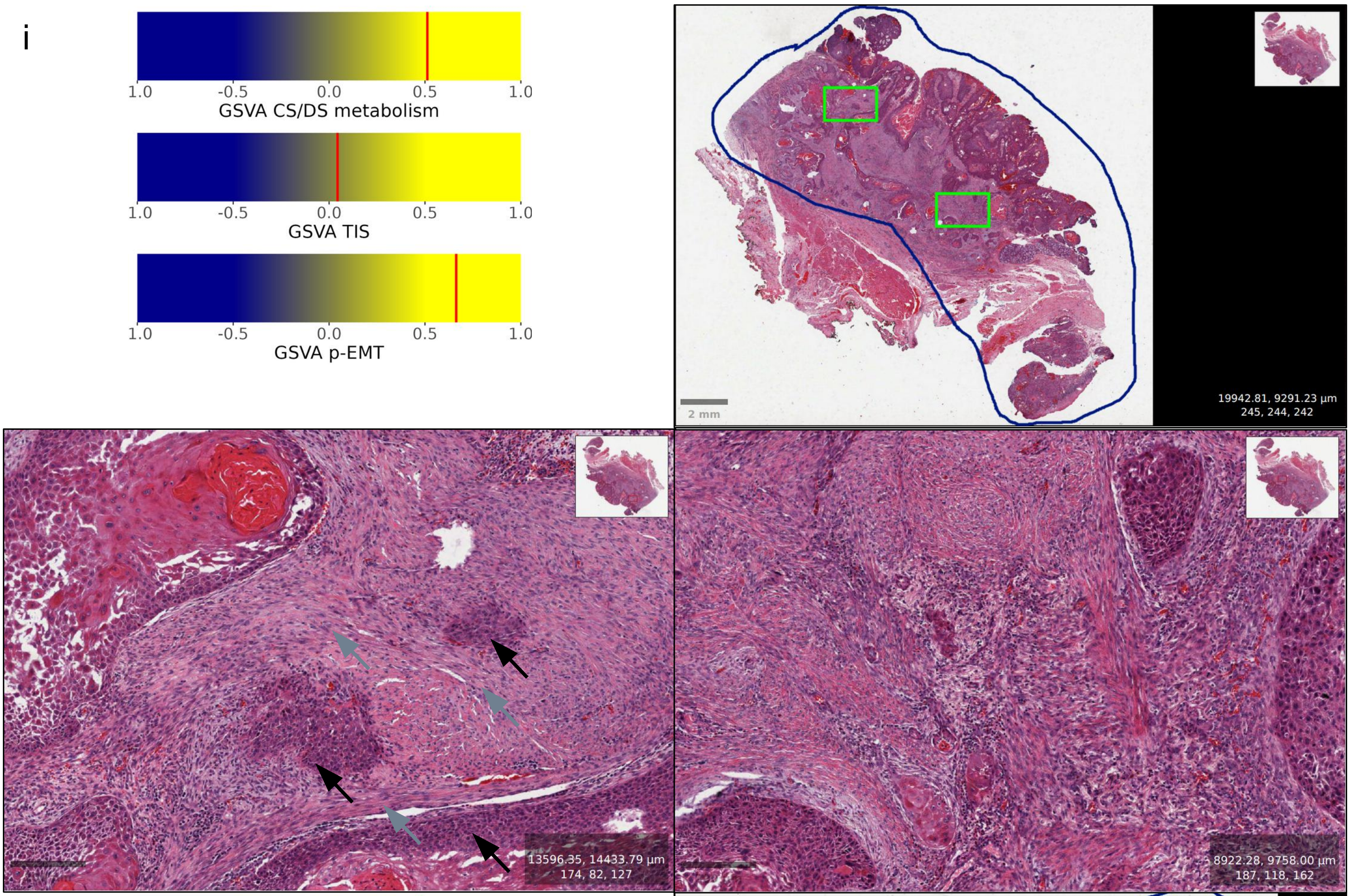
1.0 -0.5 0.0 0.5 1.0
GSVA CS/DS metabolism
1.0 -0.5 0.0 0.5 1.0
GSVA TIS
1.0 -0.5 0.0 0.5 1.0
GSVA p-EMT
2 mm
19942.81, 9291.23 µm
245, 244, 242
13596.35, 14433.79 µm
174, 82, 127
8922.28, 9758.00 µm
187, 118, 162

j

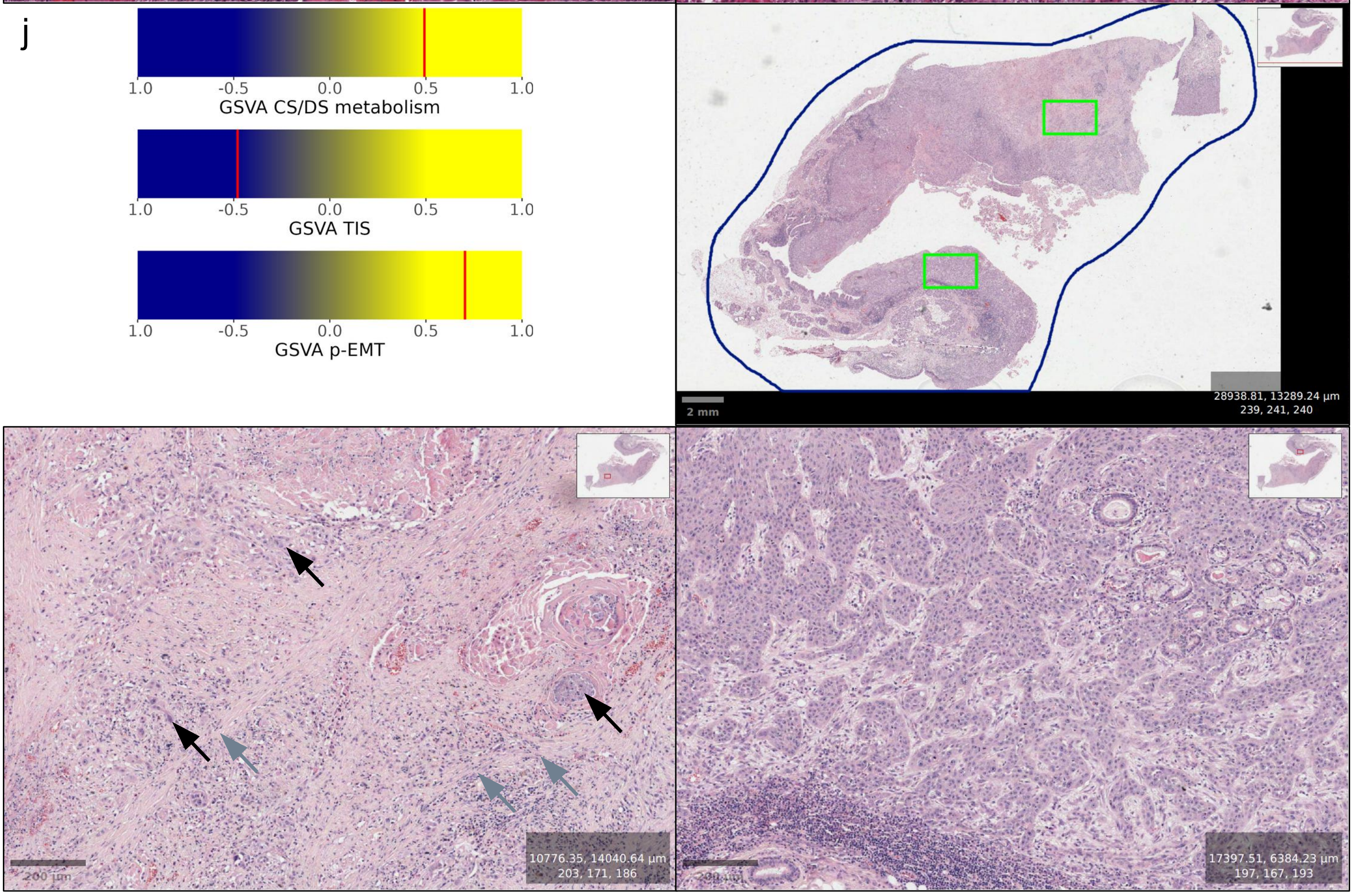
1.0 -0.5 0.0 0.5 1.0
GSVA CS/DS metabolism
1.0 -0.5 0.0 0.5 1.0
GSVA TIS
1.0 -0.5 0.0 0.5 1.0
GSVA p-EMT
2 mm
28938.81, 13289.24 µm
239, 241, 240
200 µm
10776.35, 14040.64 µm
203, 171, 186
17397.51, 6384.23 µm
197, 167, 193

TCGA HPV-negative MPS2 GSVA CS/DS metabolism low cases

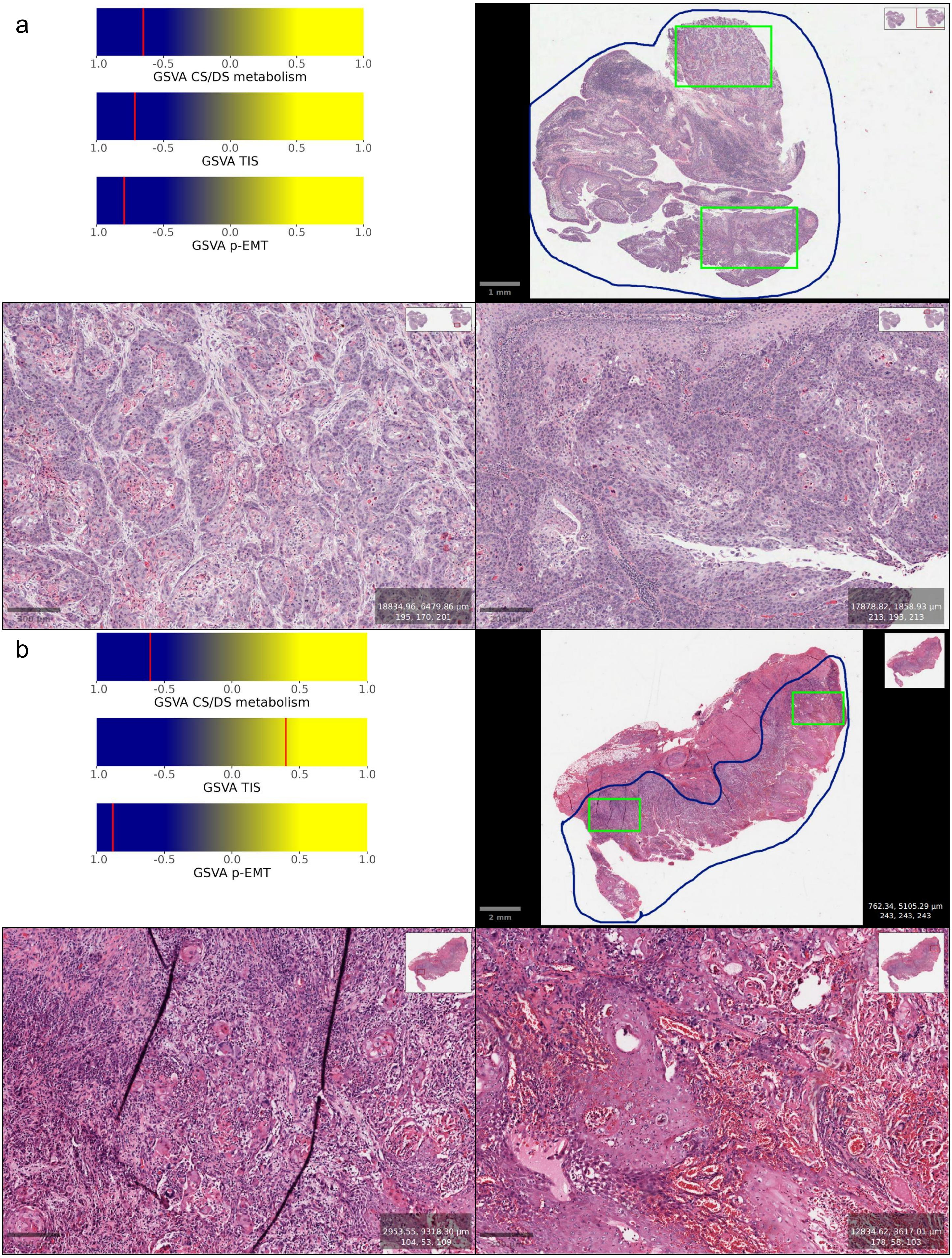

c

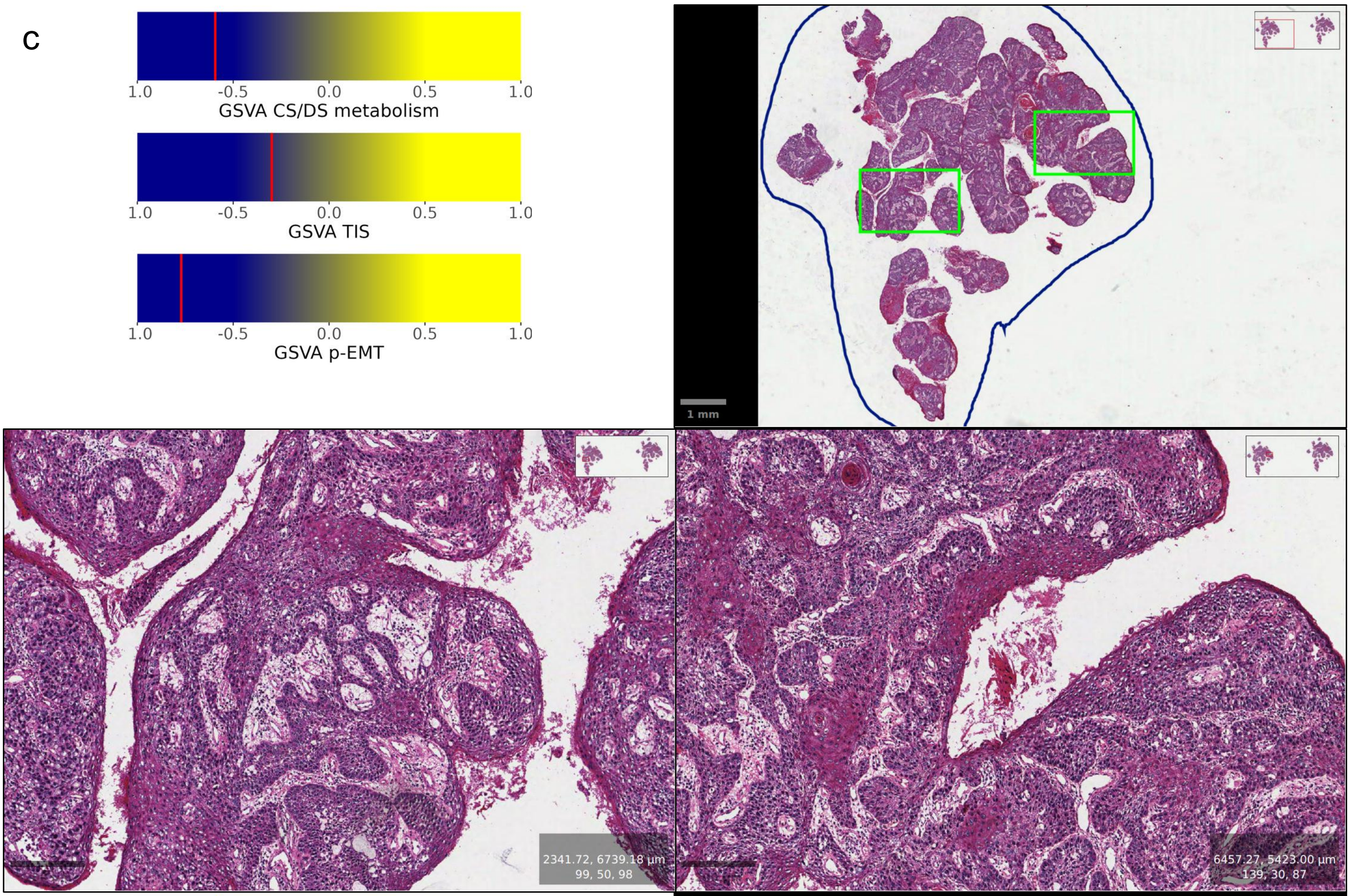
1.0 -0.5 0.0 0.5 1.0
GSVA CS/DS metabolism
1.0 -0.5 0.0 0.5 1.0
GSVA TIS
1.0 -0.5 0.0 0.5 1.0
GSVA p-EMT
1 mm
2341.72, 6739.18 µm
99, 50, 98
6457.27, 5423.00 µm
139, 30, 87

d

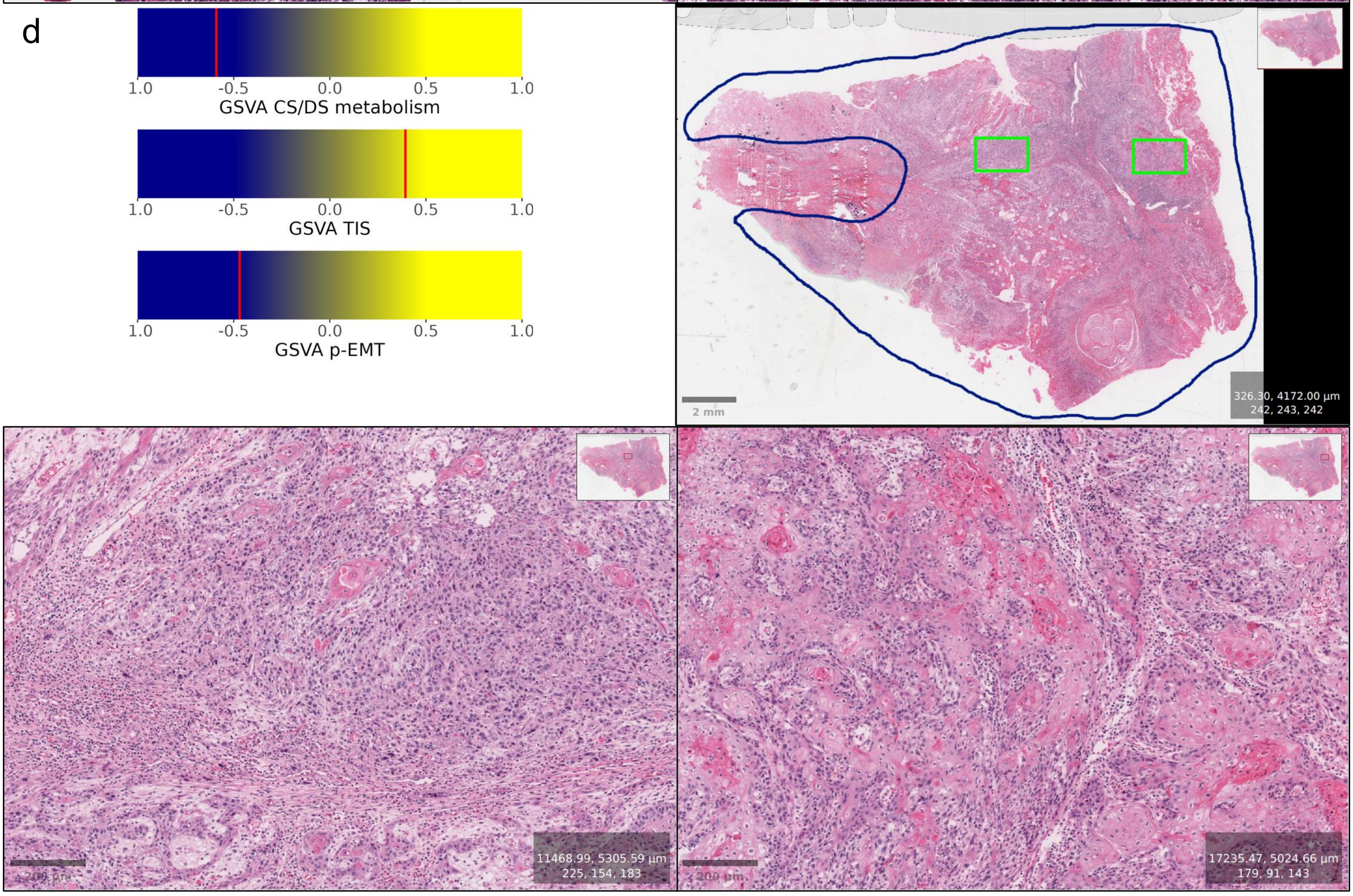
1.0 -0.5 0.0 0.5 1.0
GSVA CS/DS metabolism
1.0 -0.5 0.0 0.5 1.0
GSVA TIS
1.0 -0.5 0.0 0.5 1.0
GSVA p-EMT
2 mm
326.30, 4172.00 µm
242, 243, 242
11468.99, 5305.59 µm
225, 154, 183
17235.47, 5024.66 µm
179, 91, 143

e

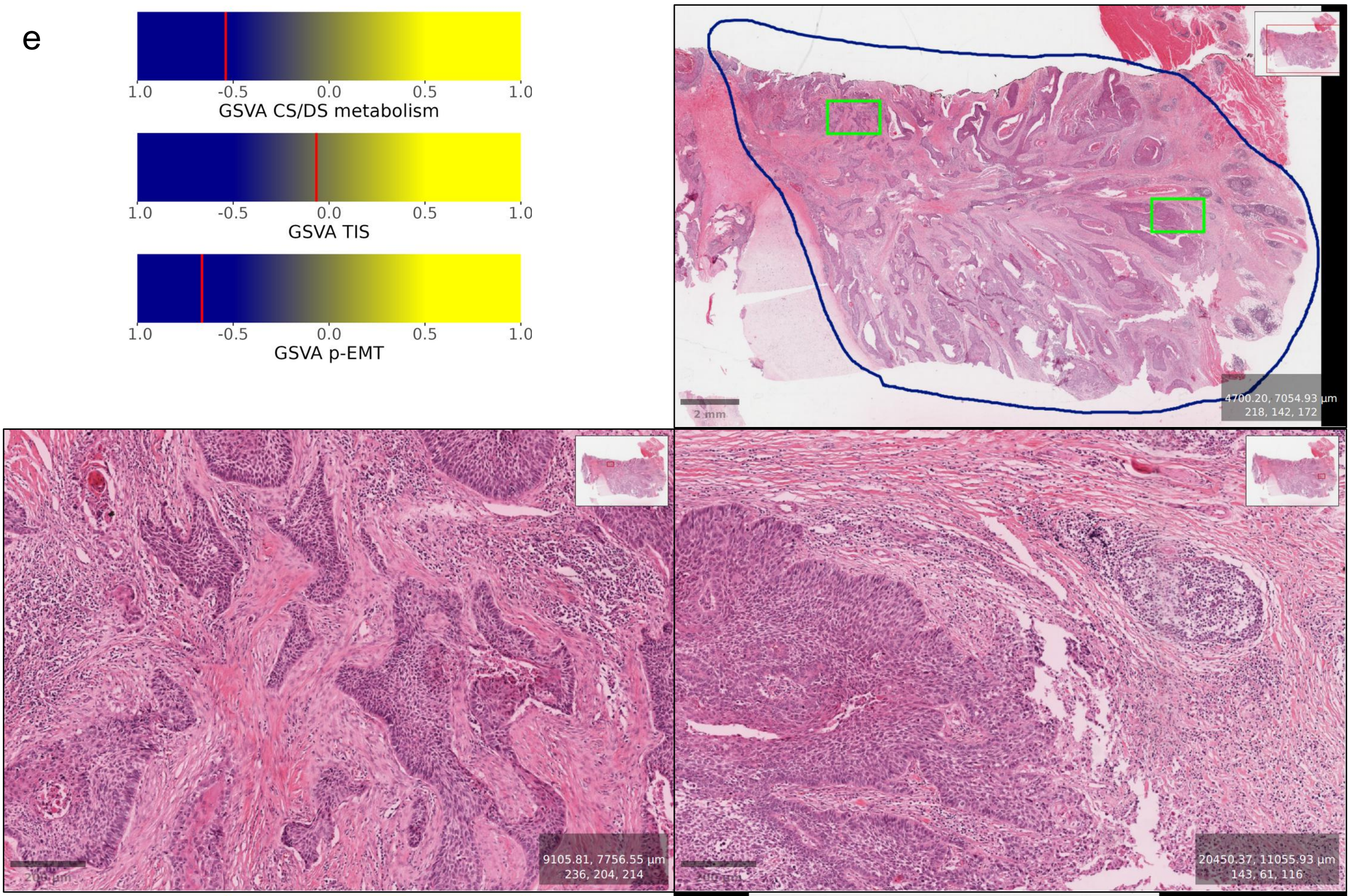
1.0 -0.5 0.0 0.5 1.0
GSVA CS/DS metabolism
1.0 -0.5 0.0 0.5 1.0
GSVA TIS
1.0 -0.5 0.0 0.5 1.0
GSVA p-EMT
2 mm
4700.20, 7054.93 µm
218, 142, 172
9105.81, 7756.55 µm
236, 204, 214
20450.37, 11055.93 µm
143, 61, 116

f

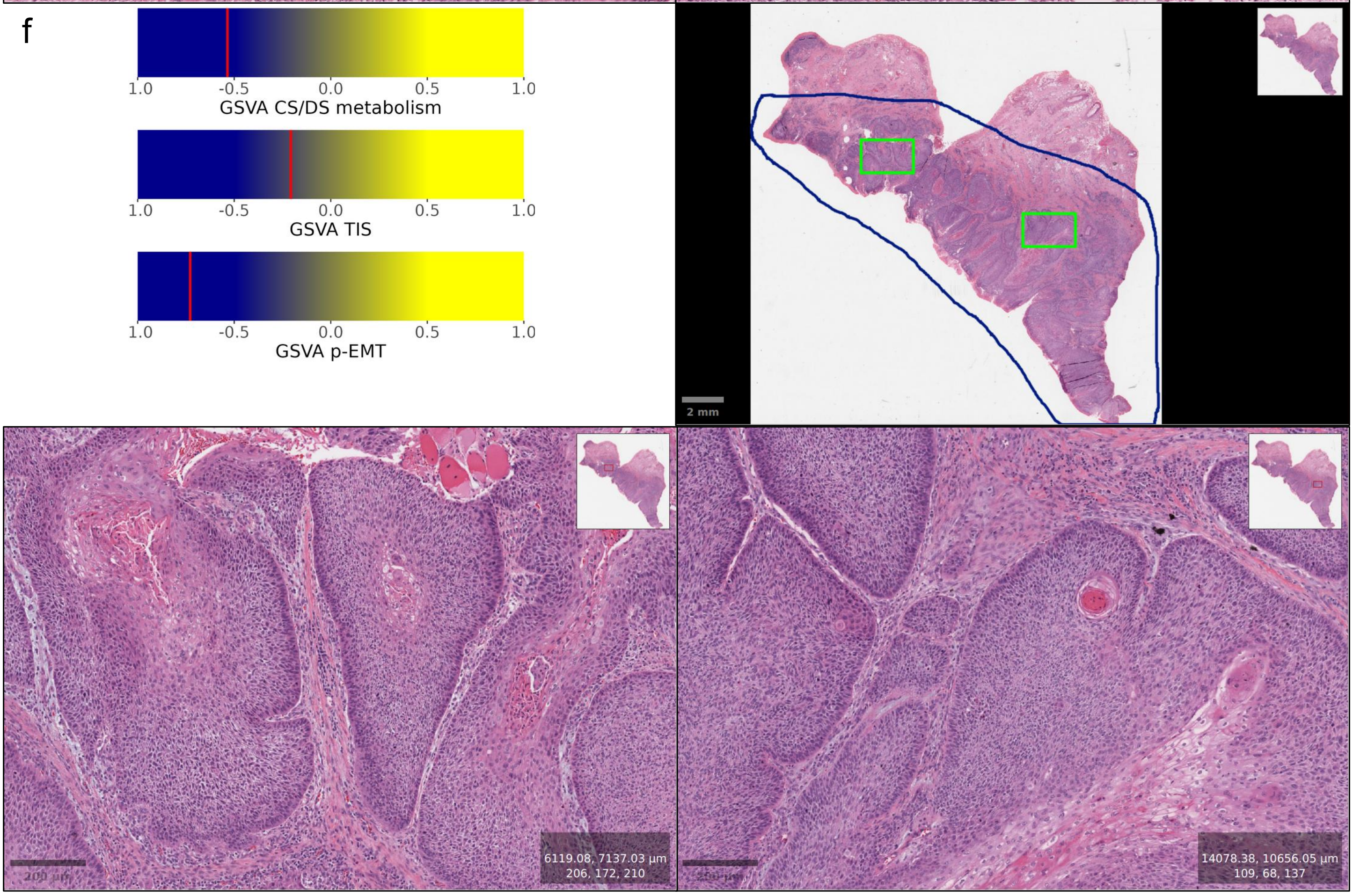
1.0 -0.5 0.0 0.5 1.0
GSVA CS/DS metabolism
1.0 -0.5 0.0 0.5 1.0
GSVA TIS
1.0 -0.5 0.0 0.5 1.0
GSVA p-EMT
2 mm
6119.08, 7137.03 µm
206, 172, 210
14078.38, 10656.05 µm
109, 68, 137

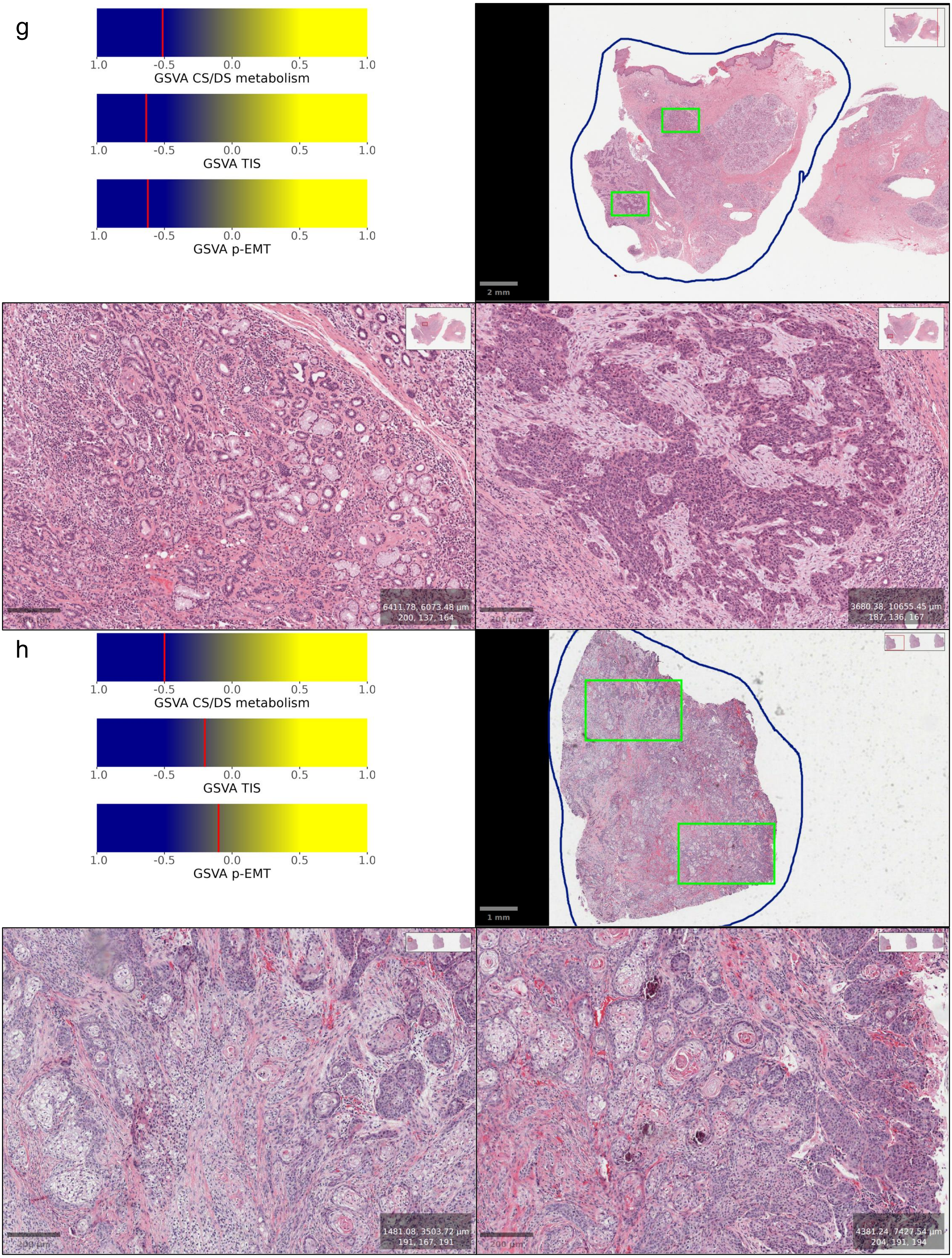
g
1.0 -0.5 0.0 0.5 1.0
GSVA CS/DS metabolism
1.0 -0.5 0.0 0.5 1.0
GSVA TIS
1.0 -0.5 0.0 0.5 1.0
GSVA p-EMT
2 mm
6411.78, 6073.48 µm
200, 137, 164
3680.38, 10655.45 µm
187, 136, 167
h
1.0 -0.5 0.0 0.5 1.0
GSVA CS/DS metabolism
1.0 -0.5 0.0 0.5 1.0
GSVA TIS
1.0 -0.5 0.0 0.5 1.0
GSVA p-EMT
1 mm
1481.08, 3503.72 µm
191, 167, 191
4381.24, 7427.54 µm
204, 191, 194

i

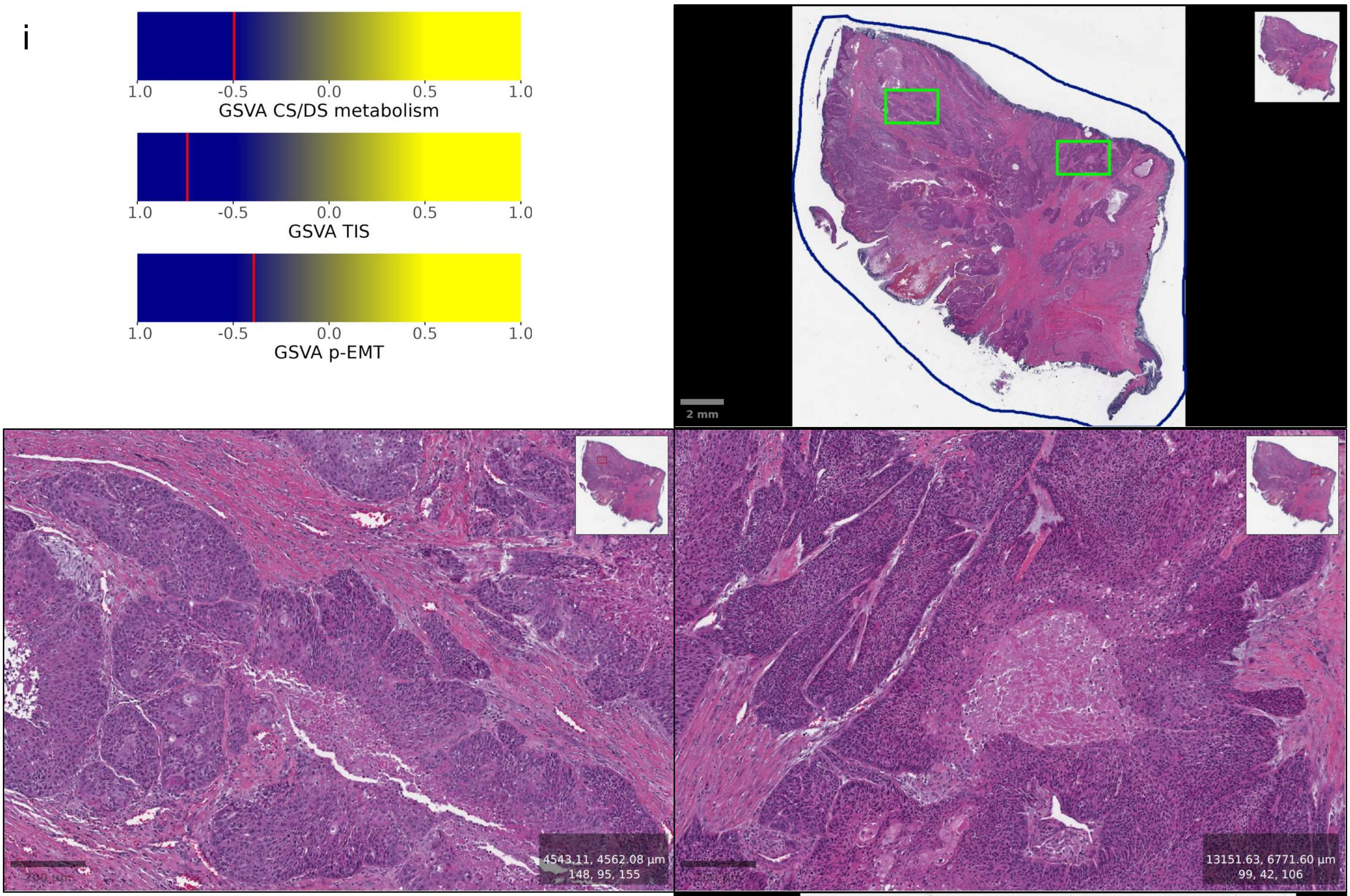
1.0 -0.5 0.0 0.5 1.0
GSVA CS/DS metabolism
1.0 -0.5 0.0 0.5 1.0
GSVA TIS
1.0 -0.5 0.0 0.5 1.0
GSVA p-EMT
2 mm
4543.11, 4562.08 µm
148, 95, 155
13151.63, 6771.60 µm
99, 42, 106

j

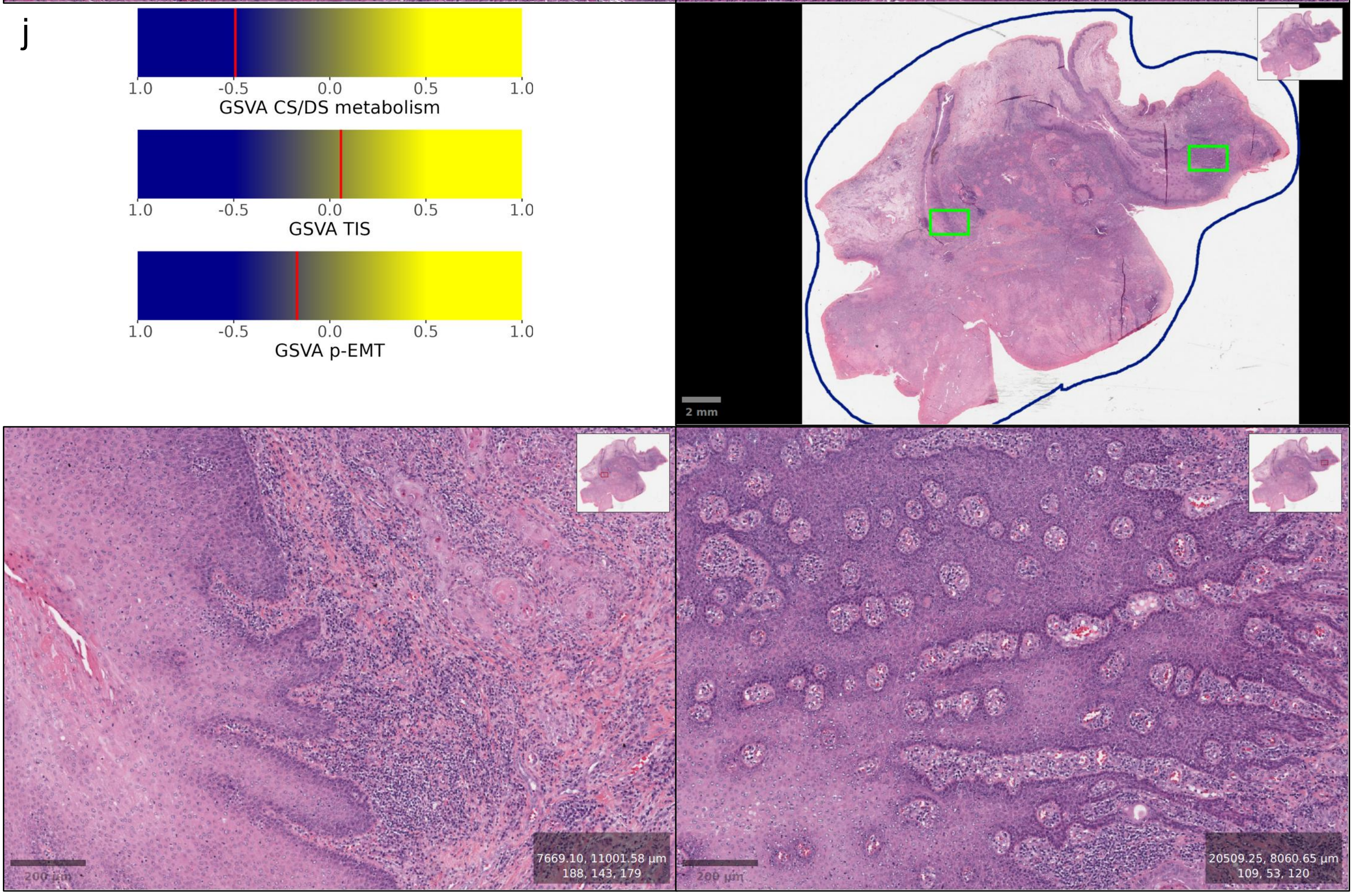
1.0 -0.5 0.0 0.5 1.0
GSVA CS/DS metabolism
1.0 -0.5 0.0 0.5 1.0
GSVA TIS
1.0 -0.5 0.0 0.5 1.0
GSVA p-EMT
2 mm
7669.10, 11001.58 µm
188, 143, 179
200 µm
20509.25, 8060.65 µm
109, 53, 120
200 µm

Supplementary Figures IHC slides

LMU HPV-negative MPS1 GSVA CS/DS high cases

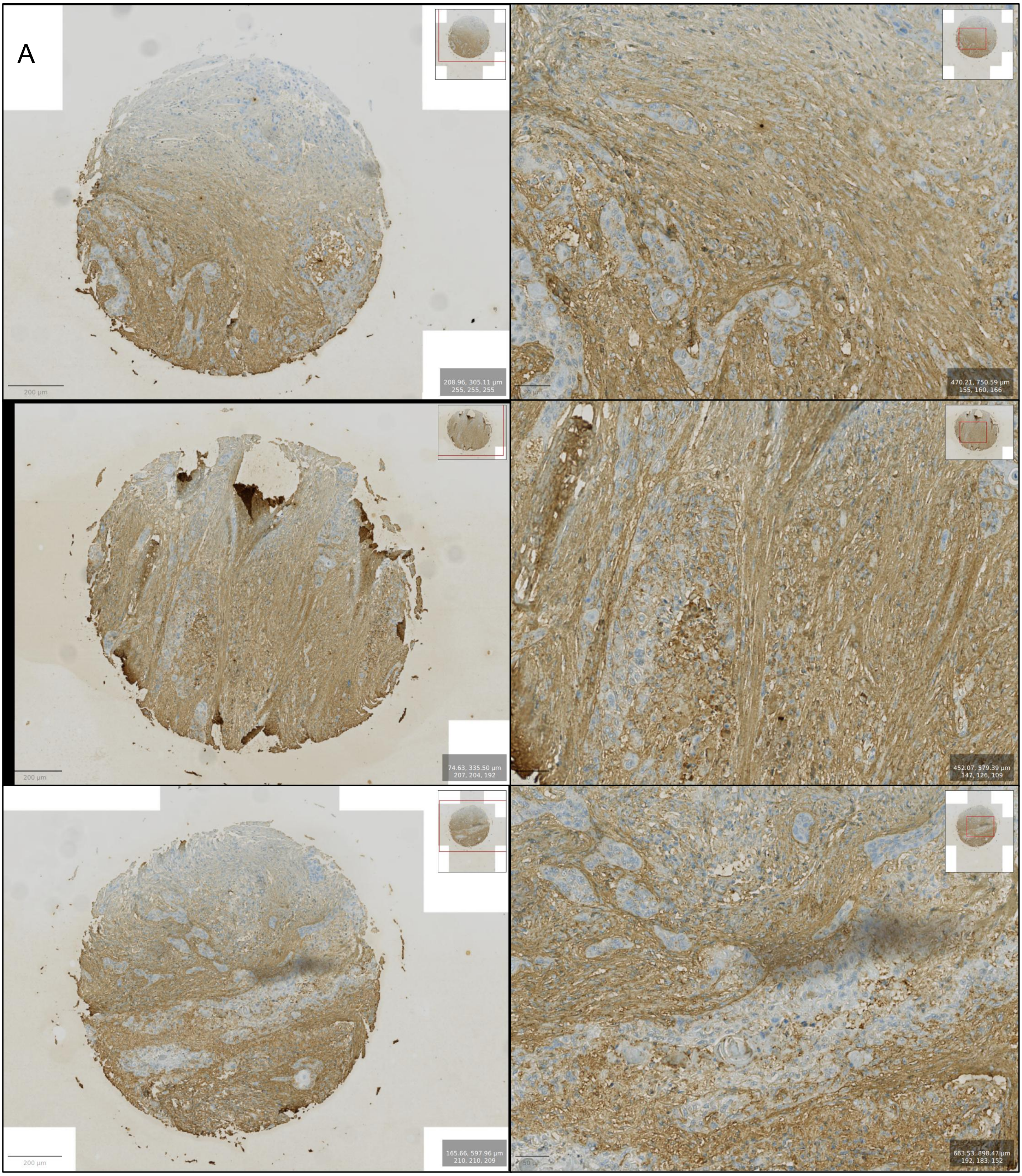

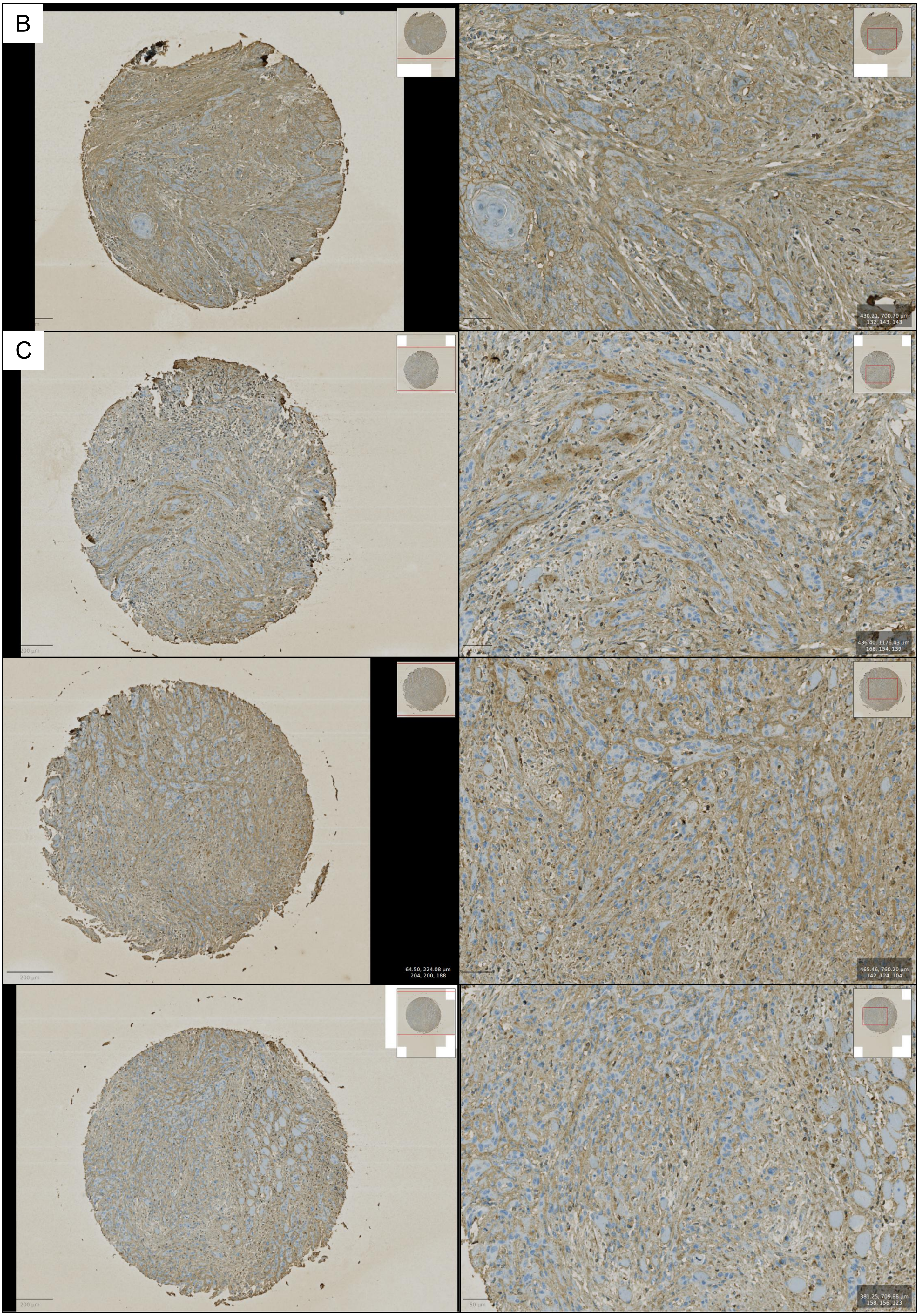
B
C
200 µm
50 µm

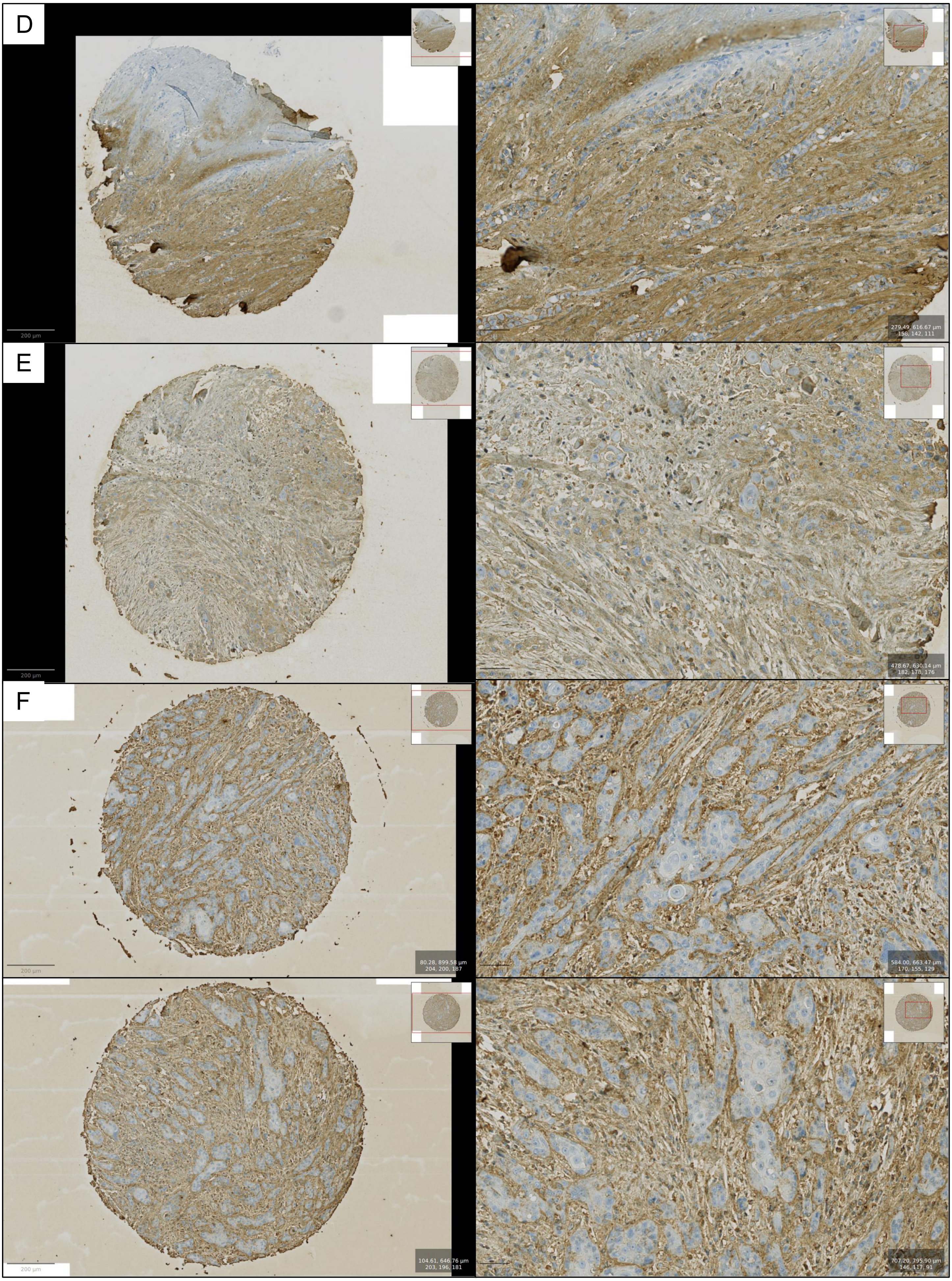
D
E
F
200 µm

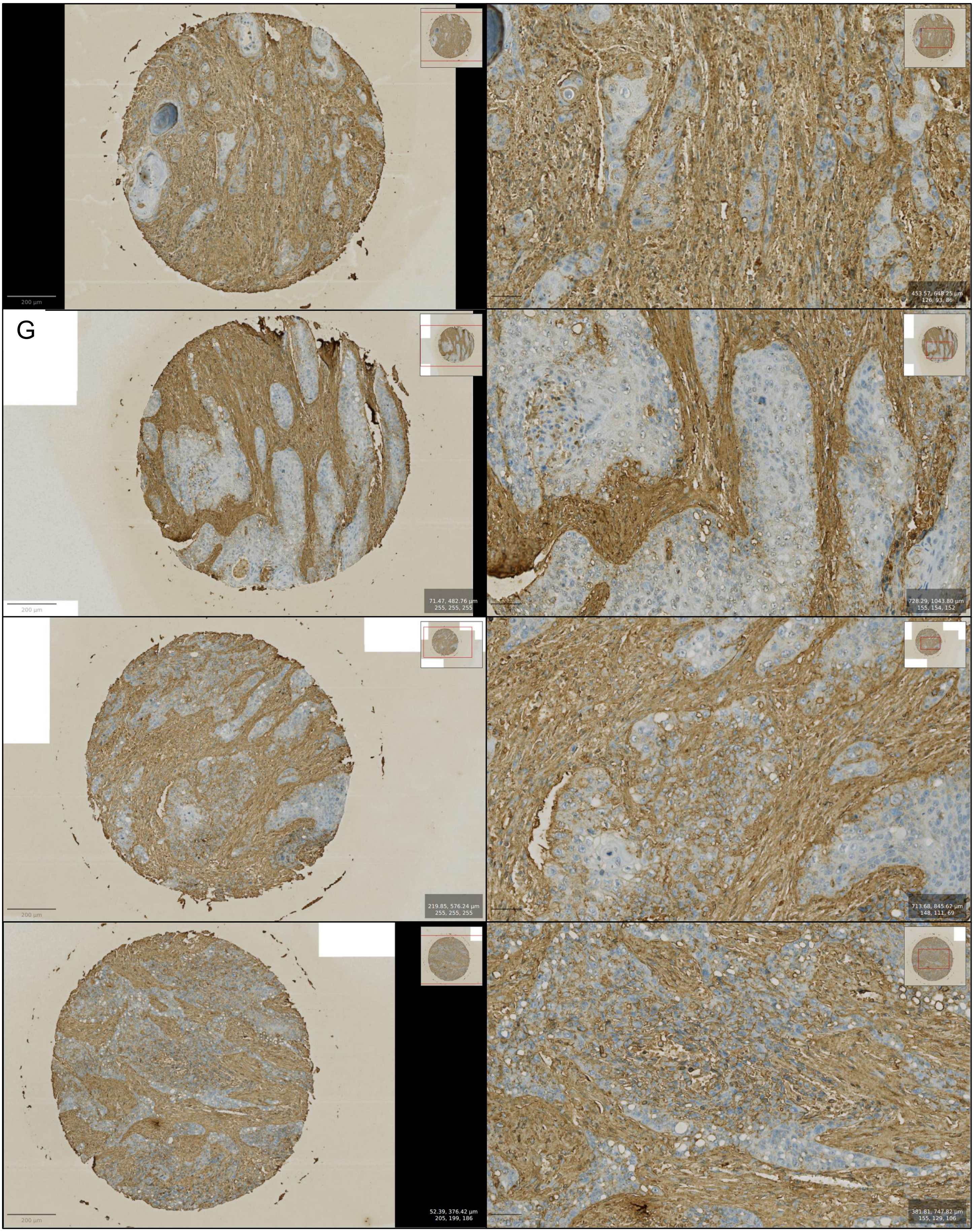

200 µm
453.57, 648.25 µm
126, 93, 86
G
71.47, 482.76 µm
255, 255, 255
200 µm
728.29, 1043.80 µm
155, 154, 152
219.85, 576.24 µm
255, 255, 255
200 µm
713.68, 845.62 µm
148, 111, 69
52.39, 376.42 µm
205, 199, 186
200 µm
381.81, 747.82 µm
155, 129, 106

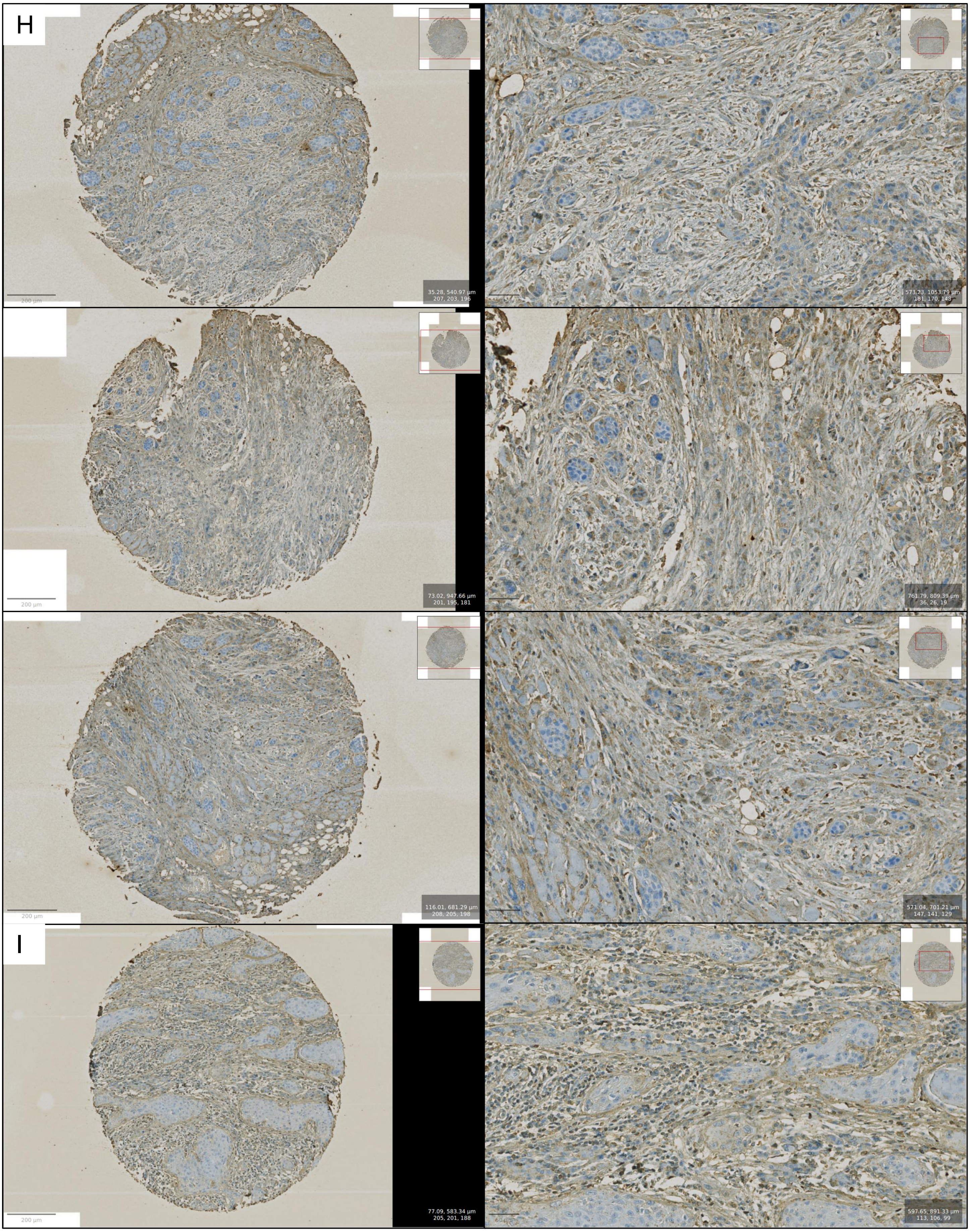
H
I

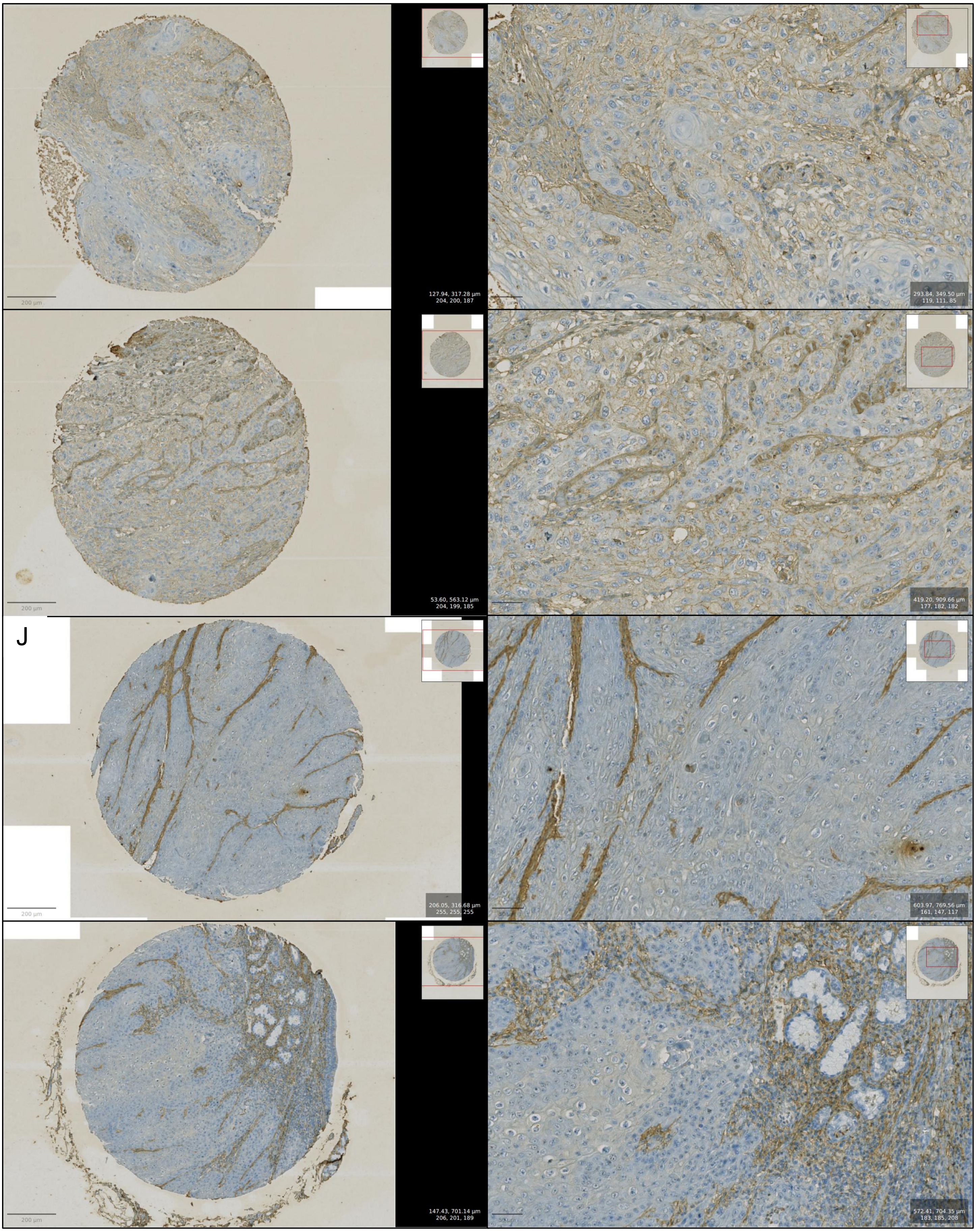

200 µm
127.94, 317.28 µm
204, 200, 187
293.84, 349.50 µm
119, 111, 85
200 µm
53.60, 563.12 µm
204, 199, 185
419.20, 909.66 µm
177, 182, 182
J
200 µm
206.05, 316.68 µm
255, 255, 255
603.97, 769.56 µm
161, 147, 117
200 µm
147.43, 701.14 µm
206, 201, 189
572.41, 704.35 µm
183, 185, 208

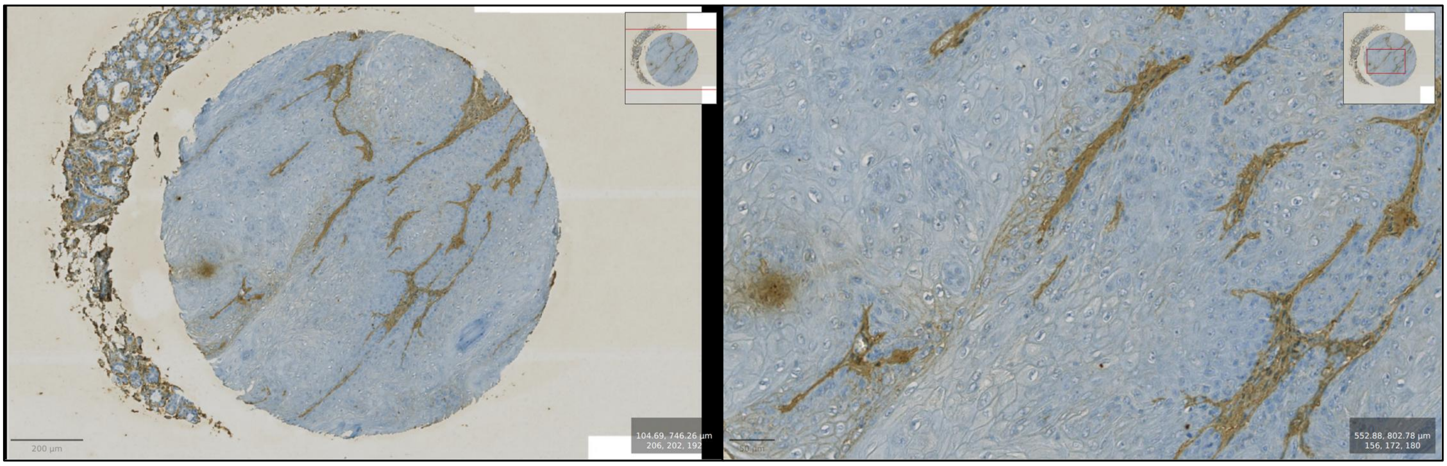

LMU HPV-negative MPS2 GSVA CS/DS low cases

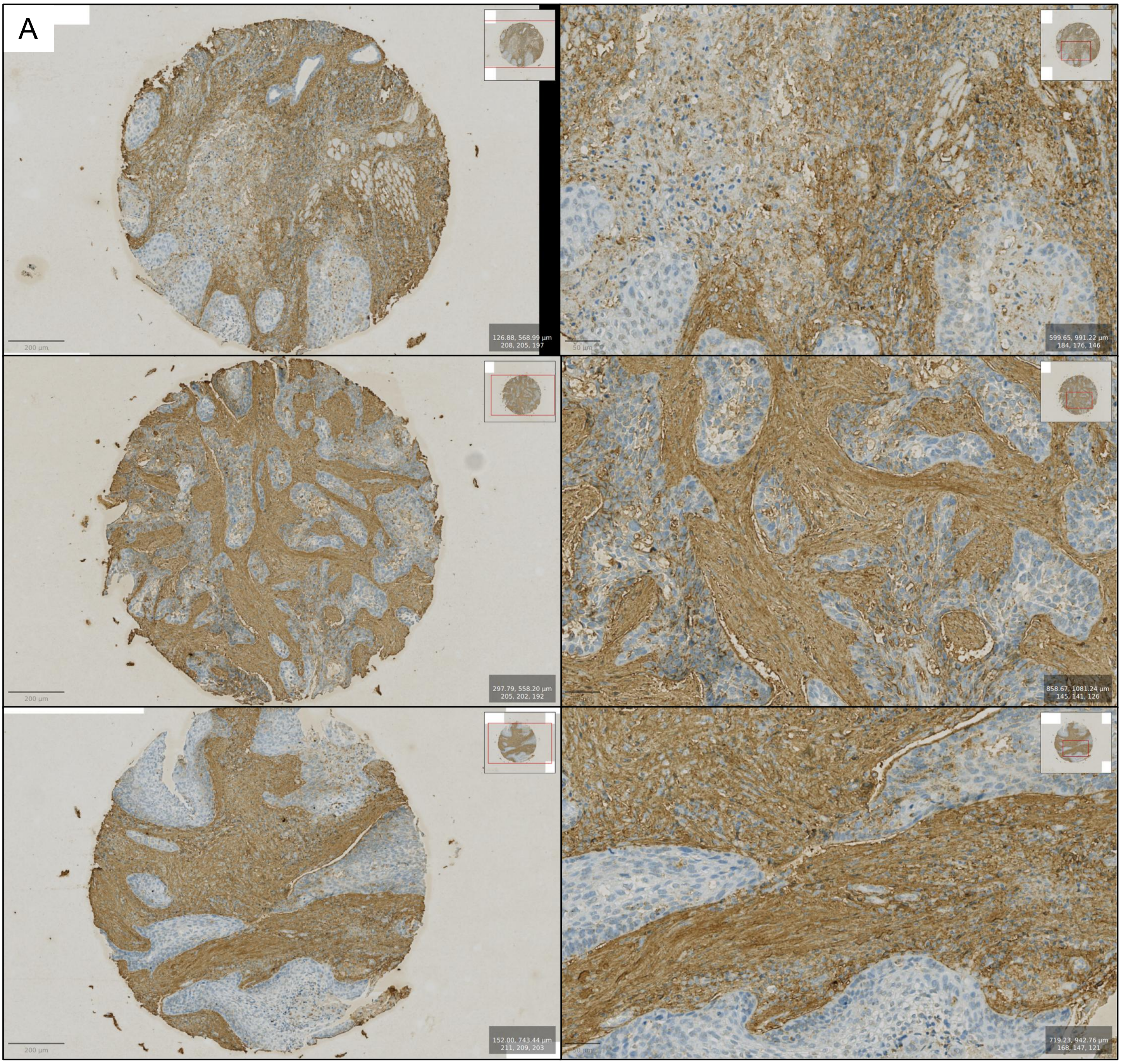

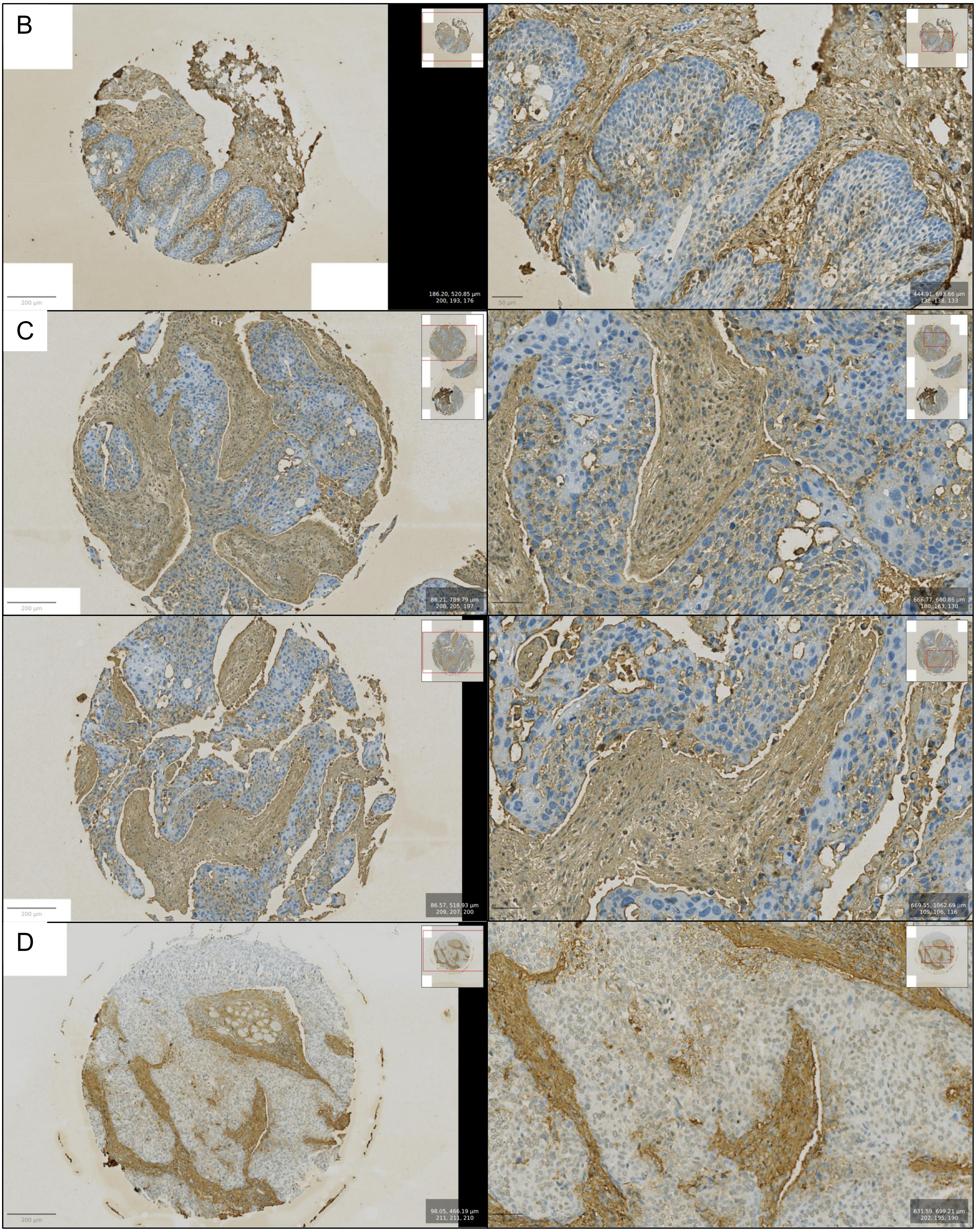
B
200 µm
186.20, 520.85 µm
200, 193, 176
50 µm
444.91, 693.66 µm
138, 138, 133
C
200 µm
88.21, 789.79 µm
208, 205, 197
666.77, 680.86 µm
180, 163, 130
200 µm
86.57, 518.93 µm
209, 207, 200
669.55, 1062.69 µm
109, 106, 116
D
200 µm
98.05, 466.19 µm
211, 211, 210
631.59, 699.21 µm
202, 195, 190

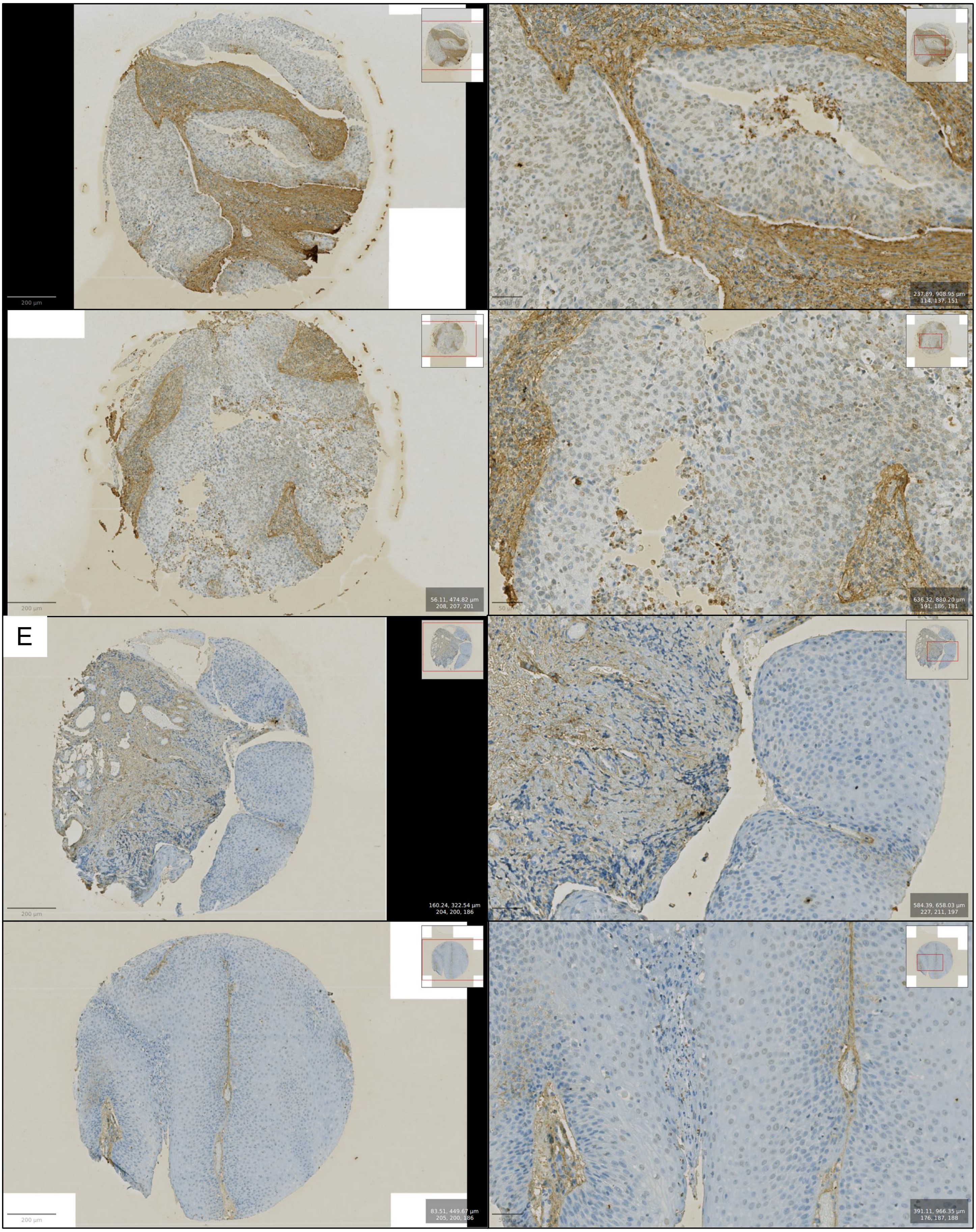
E
200 µm
237.89, 908.95 µm
114, 137, 151
56.11, 474.82 µm
208, 207, 201
636.32, 880.20 µm
191, 186, 181
160.24, 322.54 µm
204, 200, 186
584.39, 658.03 µm
227, 211, 197
83.51, 449.67 µm
205, 200, 186
391.11, 966.35 µm
176, 187, 188

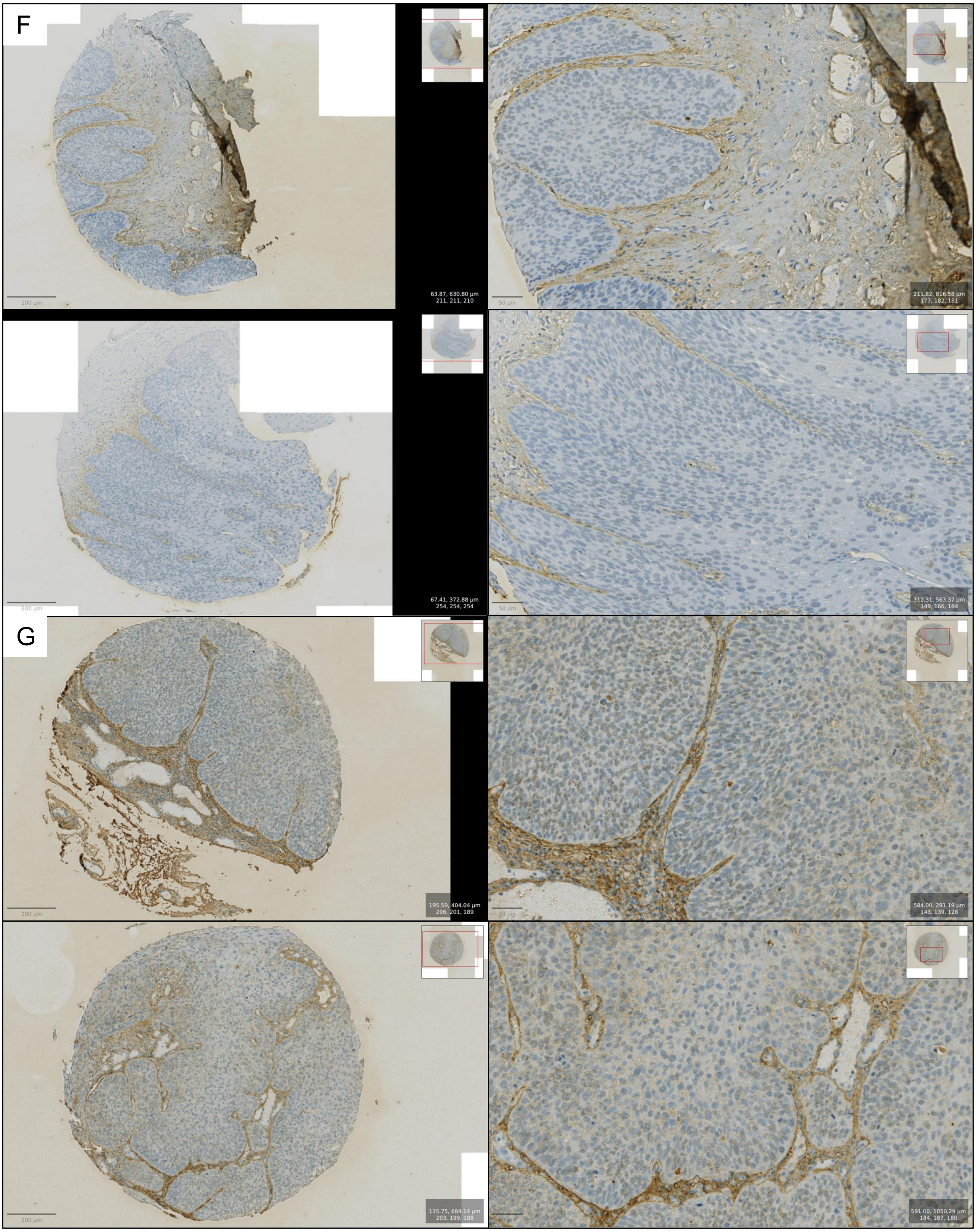
F
G
200 µm
50 µm

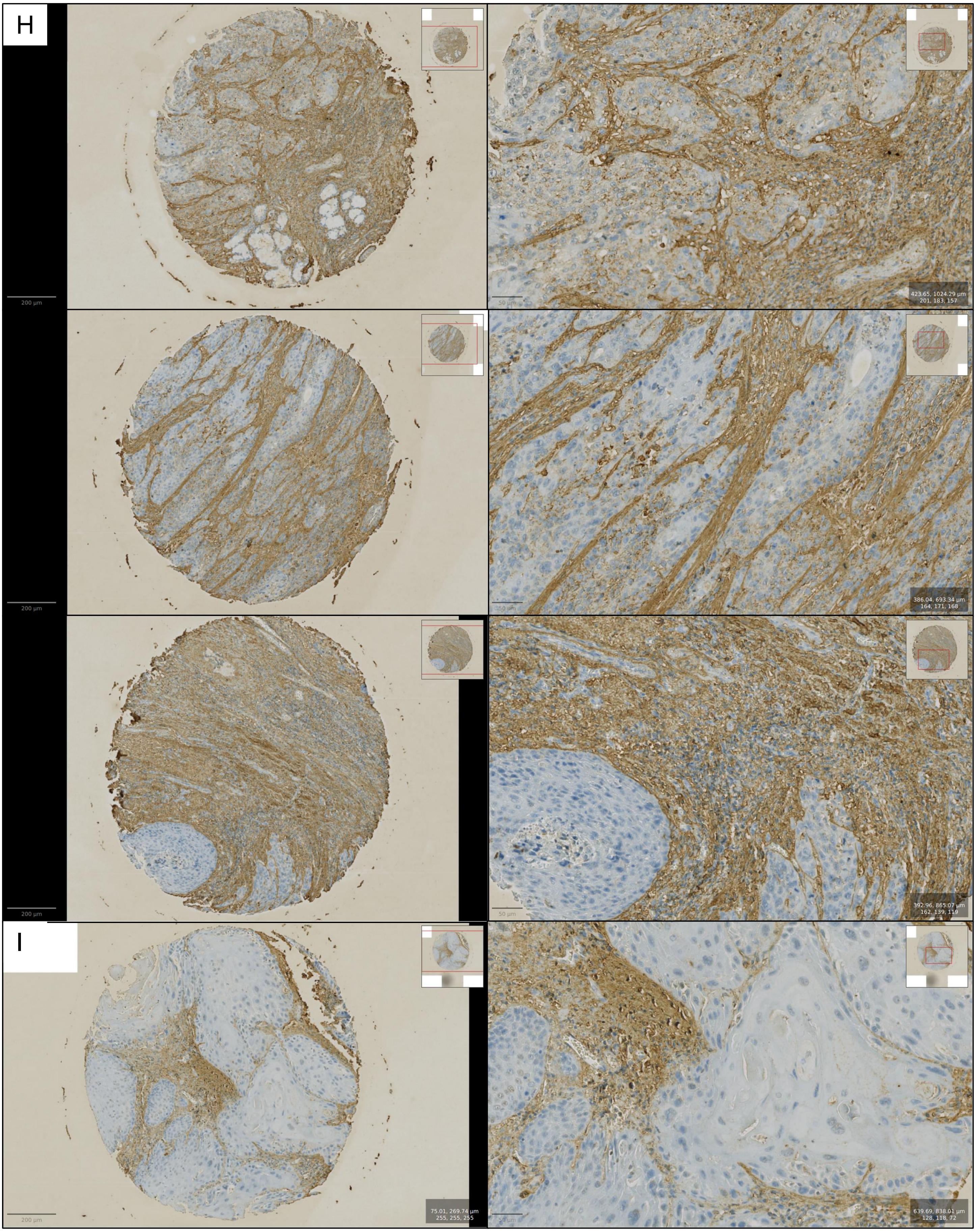
H
I
200 µm
50 µm

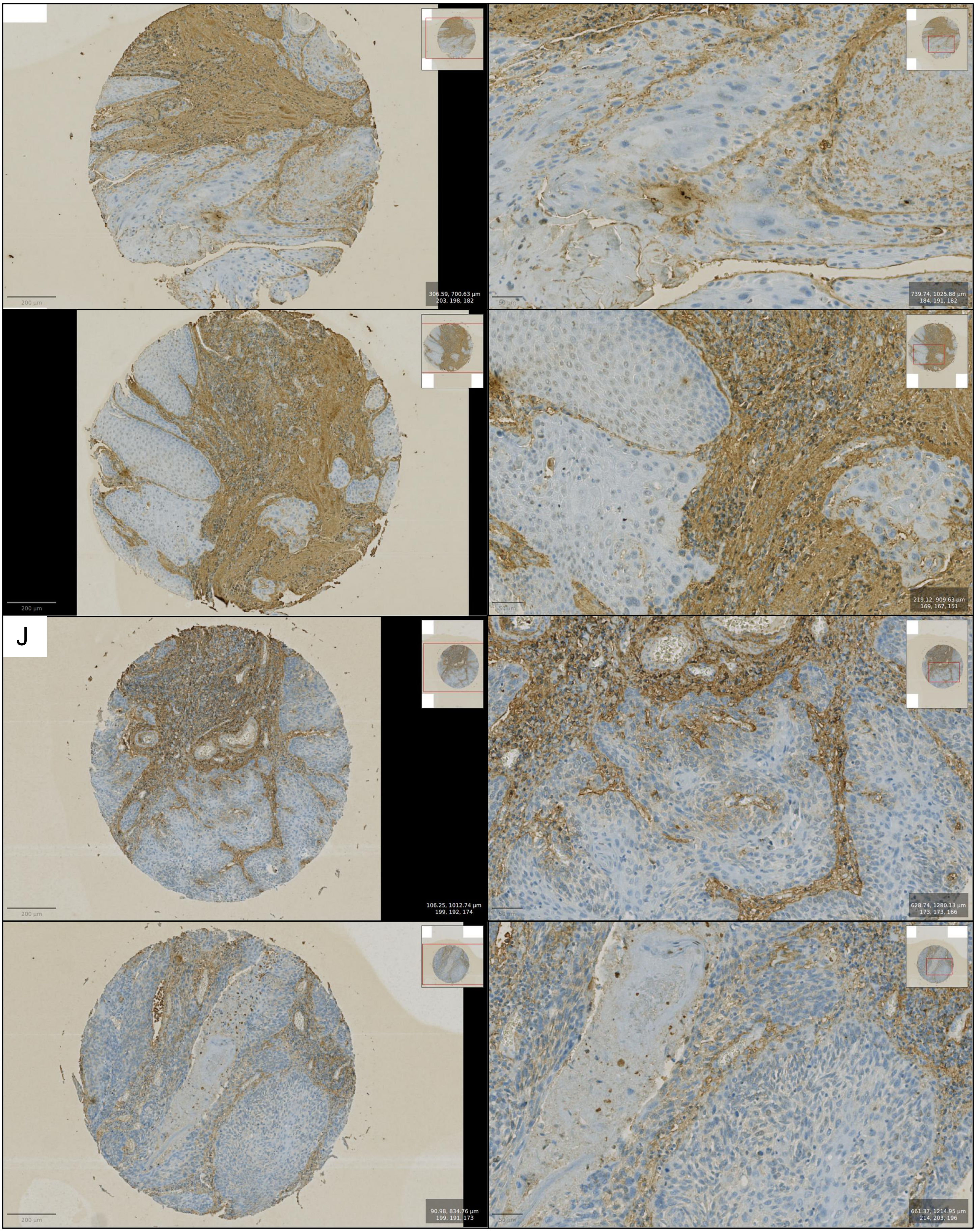
J
200 µm
306.59, 700.63 µm
203, 198, 182
739.74, 1025.88 µm
184, 191, 182
200 µm
219.12, 909.63 µm
169, 167, 151
200 µm
106.25, 1012.74 µm
199, 192, 174
628.74, 1280.13 µm
173, 173, 166
200 µm
90.98, 834.76 µm
199, 191, 173
661.37, 1214.95 µm
214, 203, 196

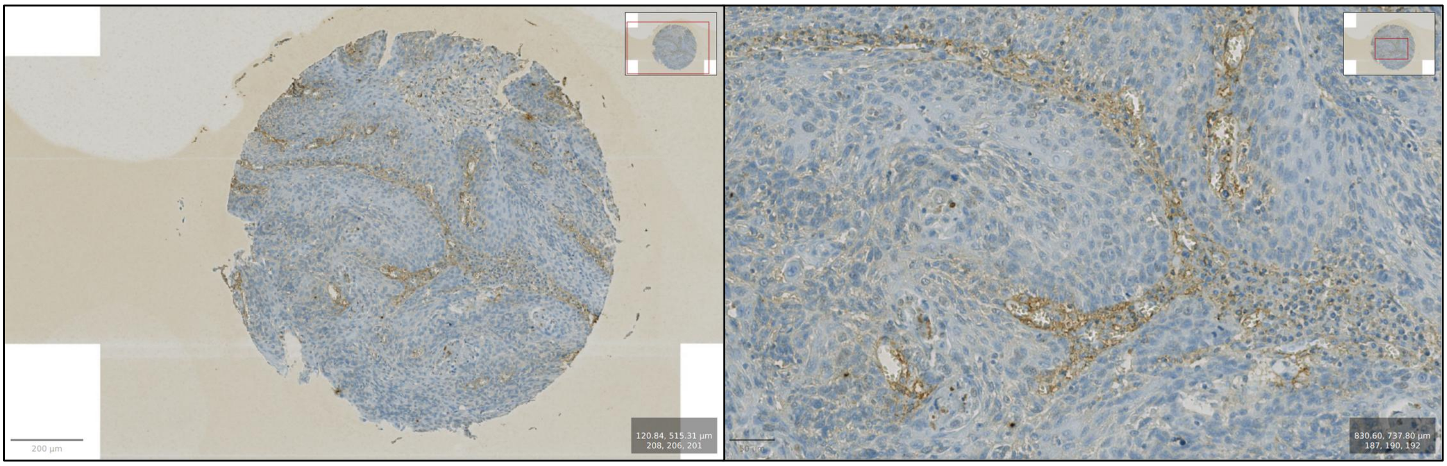
200 µm
120.84, 515.31 µm
208, 206, 201
830.60, 737.80 µm
187, 190, 192

Supplementary Figure MALDI-MSI HE

MALDI-MSI images of one exemplary MPS1 (left) and one MPS2 (right) patient tumor, respectively. Elevated chondroitin sulfate (CS) abundance (m/z=458.0605) in MPS1 compared to MPS2 malignant cells (only malignant cell tumor areas are visualized, **a**). **HE staining** of consecutive sections of the respective tumors in three magnifications (**b**)**.** MPS1 tumor with strongly intermixed malignant and TME cell populations (fibroblasts/CAFs), indicating their active interaction. MPS2 tumor with separated malignant and TME cell areas. Both MPS phenotypes exhibit the characteristic morphologies as described in detail in the main manuscript and validate the malignant cell-specific CS overexpression in MPS1 compared to MPS2 tumors (RNAseq and IHC data).

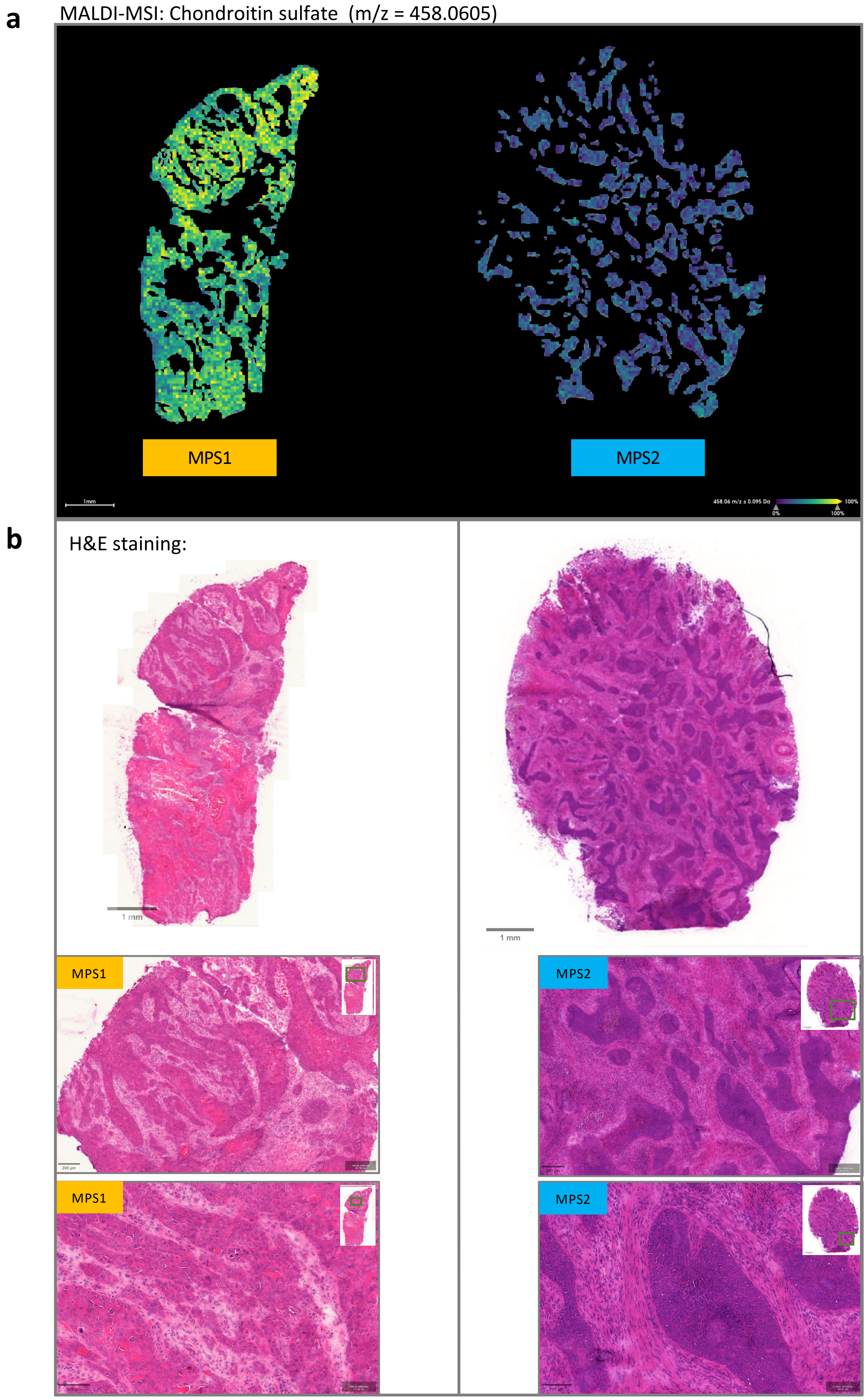

MALDI-MSI images of one exemplary MPS1 (left) and one MPS2 (right) patient tumor, respectively. Exemplary metabolites form the Energy metabolism, Amino acid metabolism, and Lipid metabolism category are visualized (malignant cells only). All metabolites indicate an elevated abundance in the MPS2 tumor (right) and confirm the findings derived from the transcipt level data, where MPS2 was associated with elevated metabolic pathway activities from multipe metabolic categories compared to MPS1 tumors.

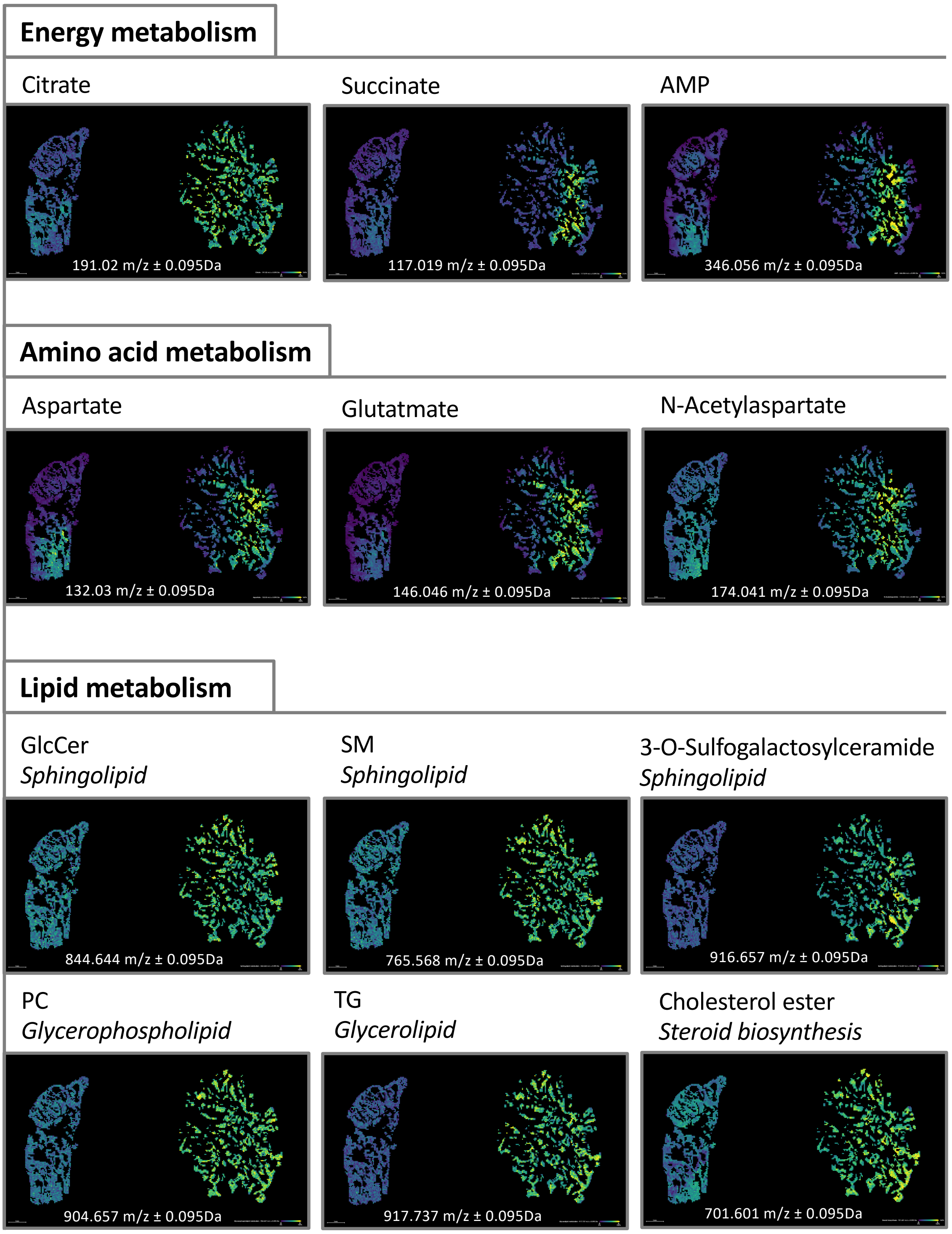

Supplementary Figure Xenograft HE

MPS orthotopic xenograft model - HE sections and CT scans: MPS1 models with UPCI-SCC 131 cells (left panel) and MPS2 models with Cal33 cells (right panel). Two replicates/mice for each MPS phenotype. The respective MPS tumors are dominated by the characteristic morphologies as described in the clinical patient tumors in the manuscript in detail. Along with the xenograft RNAseq data analysis results, the herein presented tumor morphologies support the interpretation of the differential TME formation as a consequence of the malignant cell MPS phenotype observed in MPS1 tumors.

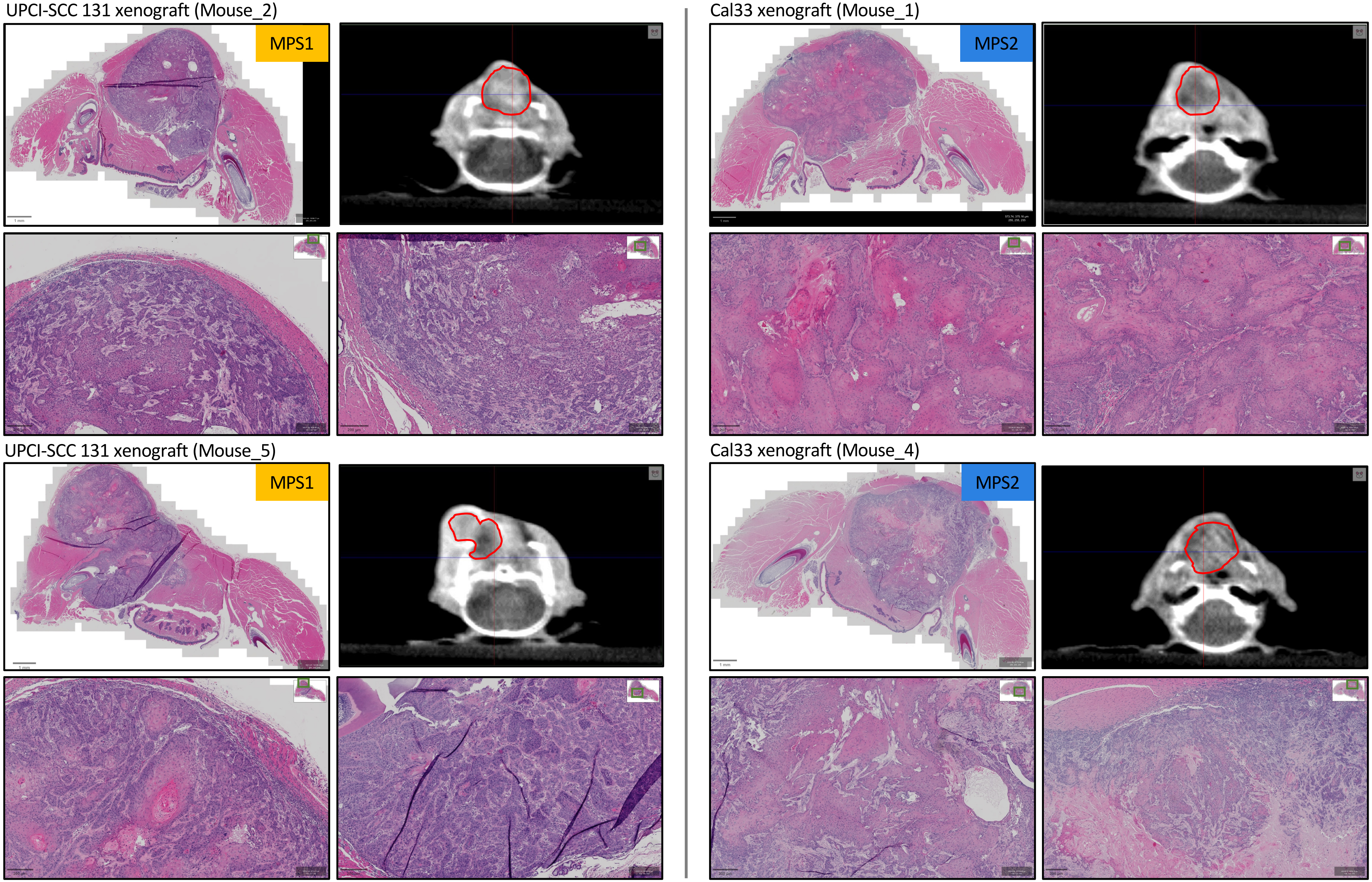

Supplementary Figure Fuel Flex Test:
The capacity and dependency to oxidize glucose, glutamine or fatty acids on the mitochondria was measured in UPCI-SCC131 (MPS1) and Cal33 (MPS2) cell lines using a Seahorse Bioanalyzer and the fuel flex assay. A decreased capacity for glucose oxidation as well as decreased dependency to oxidize glucose and glutamine were determined in UPCI-SCC131 compared to Cal33 cells.

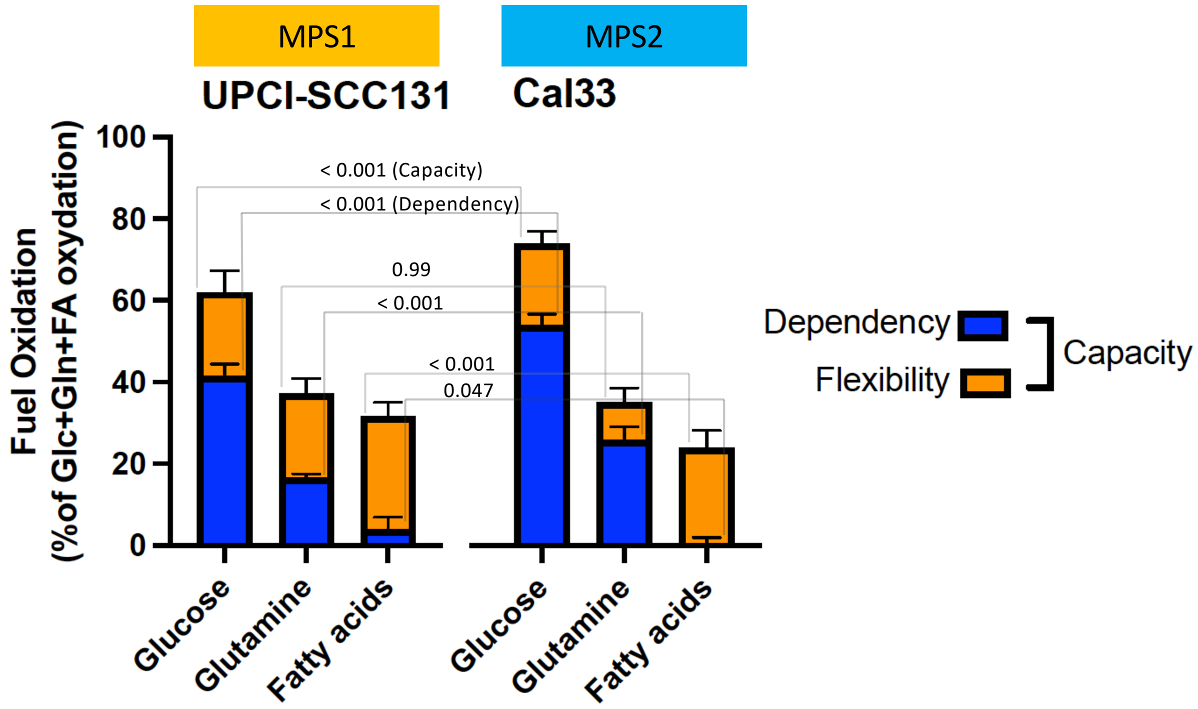

Cal33 and UPCI-SCC131 cells were plated at 20.000 cells/well in XF96 microplates (Seahorse Bioscience, Billerica, MA, USA) in DMEM high Glucose Medium with 10% FCS and 1% Pen/Strep according to manufacturer's recommendations 24 h prior to the assay. Medium was exchanged to XF DMEM medium (Seahorse Bioscience, Billerica, MA, USA) with 2 mM glutamine, 10 mM glucose, 1 mM pyruvate, and 5 mM HEPES and incubated at 37°C without CO_2 1h on the day of assay. Fuel Flex test kit applies the inhibitor of glutaminase (GLS1) BPTES (3 µM), the inhibitor of carnitine palmitoyl-transferase 1 A (CPT1A) etomoxir (4 µM) and the inhibitor of the mitochondrial pyruvate carrier (MPC) UK5099 (2 µM) in different combinations according to manufacturer’s protocol to determine cellular metabolic capacity flexibility and dependency as previously described. The flexibility to oxidize other fuel when the main pathway was inhibited, was assessed according to the manufacturers protocol. Percentage of total mitochondrial respiration for each fuel pathway in Cal33 and SCC131 cell lines. Mean values and standard deviation (SD) were plotted. 2-way Anova and post-hoc Tukey test pairwise p-values indicated for “capacity” and “dependency” between UPCI-SCC131 and Cal33.
